# Supplementary material for: A Curriculum for Teaching Clinical Efficiency Focusing on Specific Communication Skills While Maximizing the Electronic Health Record
Source: MedEdPORTAL. 2020 Oct 29;16:10989. doi: 10.15766/mep_2374-8265.10989 (PMC7597939; doi:10.15766/mep_2374-8265.10989)
Supplement: Supplementary file 1 — Efficiency Preworkshop Needs Assessment Survey.docxWorkshop 1 - Setting up the Template and Working in EHR.pptxSample Clinic Note and AVS Template.docxWorkshop 2 - Preclinic Preparation and Rapport Building.pptxEfficiency ATTEND Practice Card.docxWorkshop 3 - Agenda Setting and Relationship Maintenance.pptxEfficiency Agenda Setting Practice.docxWorkshop 4 - Visit Closure.pptxEfficiency Closure Card and Cases.docxEfficiency Postworkshop Evaluation.docx [file mep_2374-8265.10989-s001.zip › A. Efficiency Preworkshop Needs Assessment Survey.docx]

**Appendix A: Preworkshop Needs Assessment Questions**

1: don’t agree, 5: strongly agree

**Pre-clinic preparation**

-- I feel prepared for every clinic session

 1 2 3 4 5

-- I have my own agenda established for each patient visit prior to the visit

 1 2 3 4 5

-- I have reviewed and updated the problem list prior to every patient visit

1 2 3 4 5

**Rapport building**

--I have good understanding regarding how to build rapport and maintain relationships with patients during clinic visits

1 2 3 4 5

--I am comfortable in practicing behaviors to build rapport and maintain relationships with patients during clinic visits

1 2 3 4 5

-- I am concerned that I unintentionally use nonverbal cues in clinical encounters that may be misinterpreted by my patients.

1 2 3 4 5

**Opening discuss and information gathering**

--I often feel frustrated when trying to get all patient concerns during a patient visit.

1 2 3 4 5

--I always try to allow my patient to complete his/her opening statement

1 2 3 4 5

**Collaborative agenda setting and reaching agreement**

--I am confident about setting an agenda with the patient

1 2 3 4 5

--I have a consistent pattern I use to set the agenda with the patient.

1 2 3 4 5

--I always make sure that the patient and I are agree about our plan through the visit

1 2 3 4 5

**Closure**

--I am frustrated when my patients bring up concerns at the end of a visit.

1 2 3 4 5

--I feel rushed at the end of visits with patients.

1 2 3 4 5

--I have a consistent pattern I use when closing a visit with a patient.

1 2 3 4 5

**Overall,**

--This Workshop will be helpful in improving my clinic efficiency

1 2 3 4 5

--I will likely to continue using some of the strategies in my future practice

1 2 3 4 5

--Do you think we should continue to provide this workshop in the future?

1 2 3 4 5
