# Supplementary material for: A Curriculum for Teaching Clinical Efficiency Focusing on Specific Communication Skills While Maximizing the Electronic Health Record
Source: MedEdPORTAL. 2020 Oct 29;16:10989. doi: 10.15766/mep_2374-8265.10989 (PMC7597939; doi:10.15766/mep_2374-8265.10989)
Supplement: Supplementary file 1 — Efficiency Preworkshop Needs Assessment Survey.docxWorkshop 1 - Setting up the Template and Working in EHR.pptxSample Clinic Note and AVS Template.docxWorkshop 2 - Preclinic Preparation and Rapport Building.pptxEfficiency ATTEND Practice Card.docxWorkshop 3 - Agenda Setting and Relationship Maintenance.pptxEfficiency Agenda Setting Practice.docxWorkshop 4 - Visit Closure.pptxEfficiency Closure Card and Cases.docxEfficiency Postworkshop Evaluation.docx [file mep_2374-8265.10989-s001.zip › B. Workshop 1 - Setting up the Template and Working in EHR.pptx]

## Slide 1
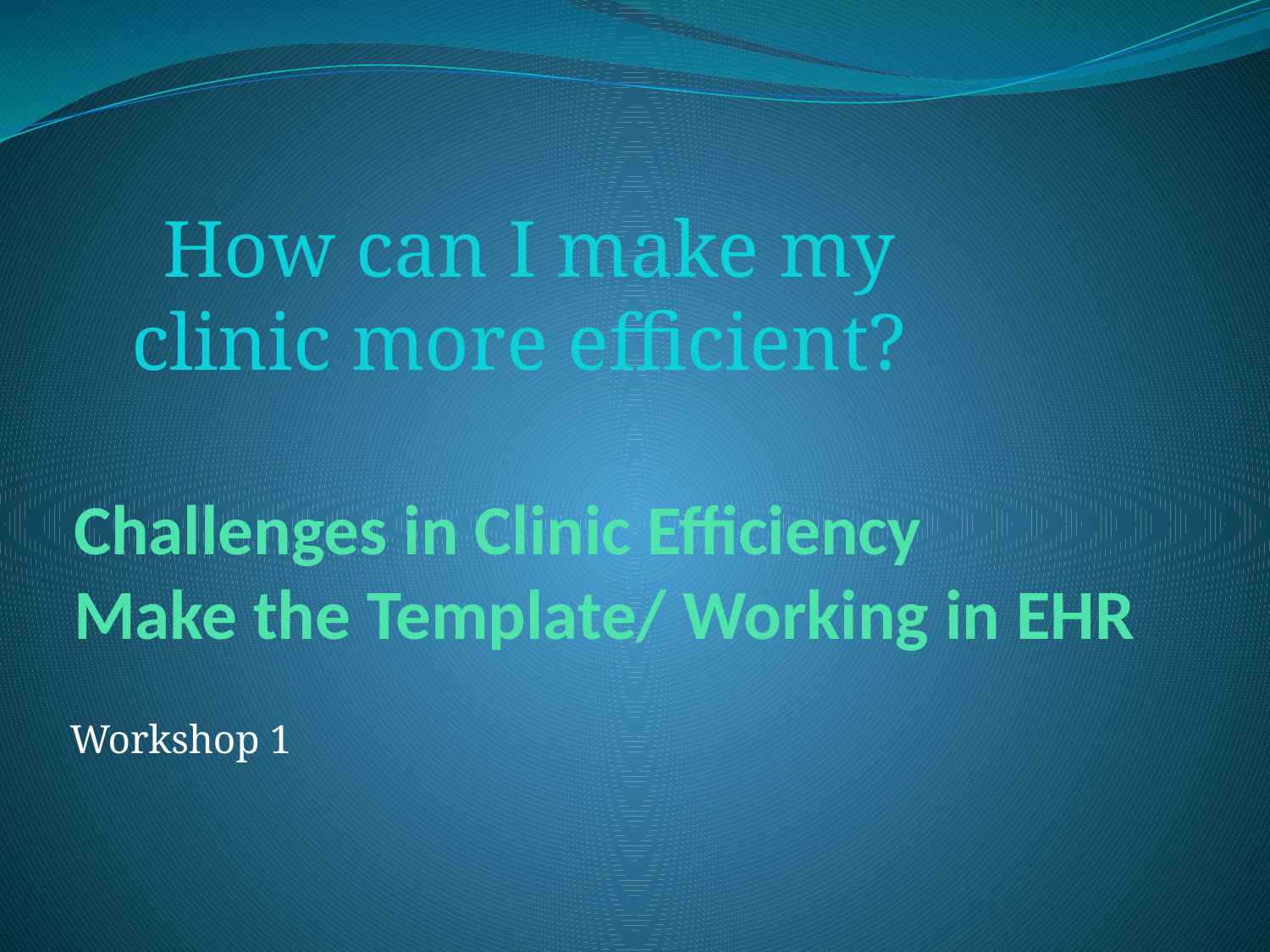

How can I make my clinic more efficient?
# Challenges in Clinic Efficiency Make the Template/ Working in EHR
Workshop 1

## Slide 2
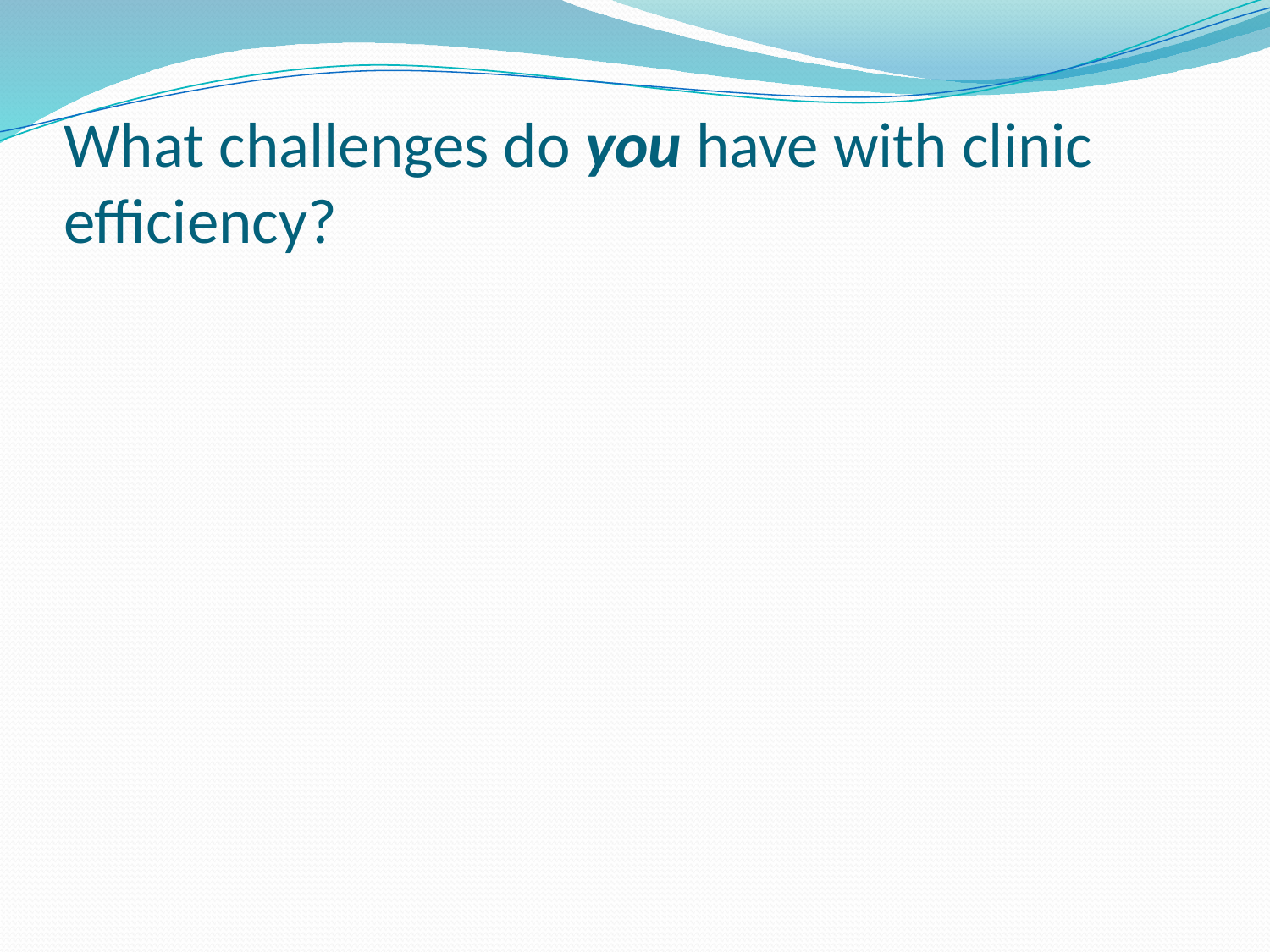

# What challenges do you have with clinic efficiency?

## Slide 3
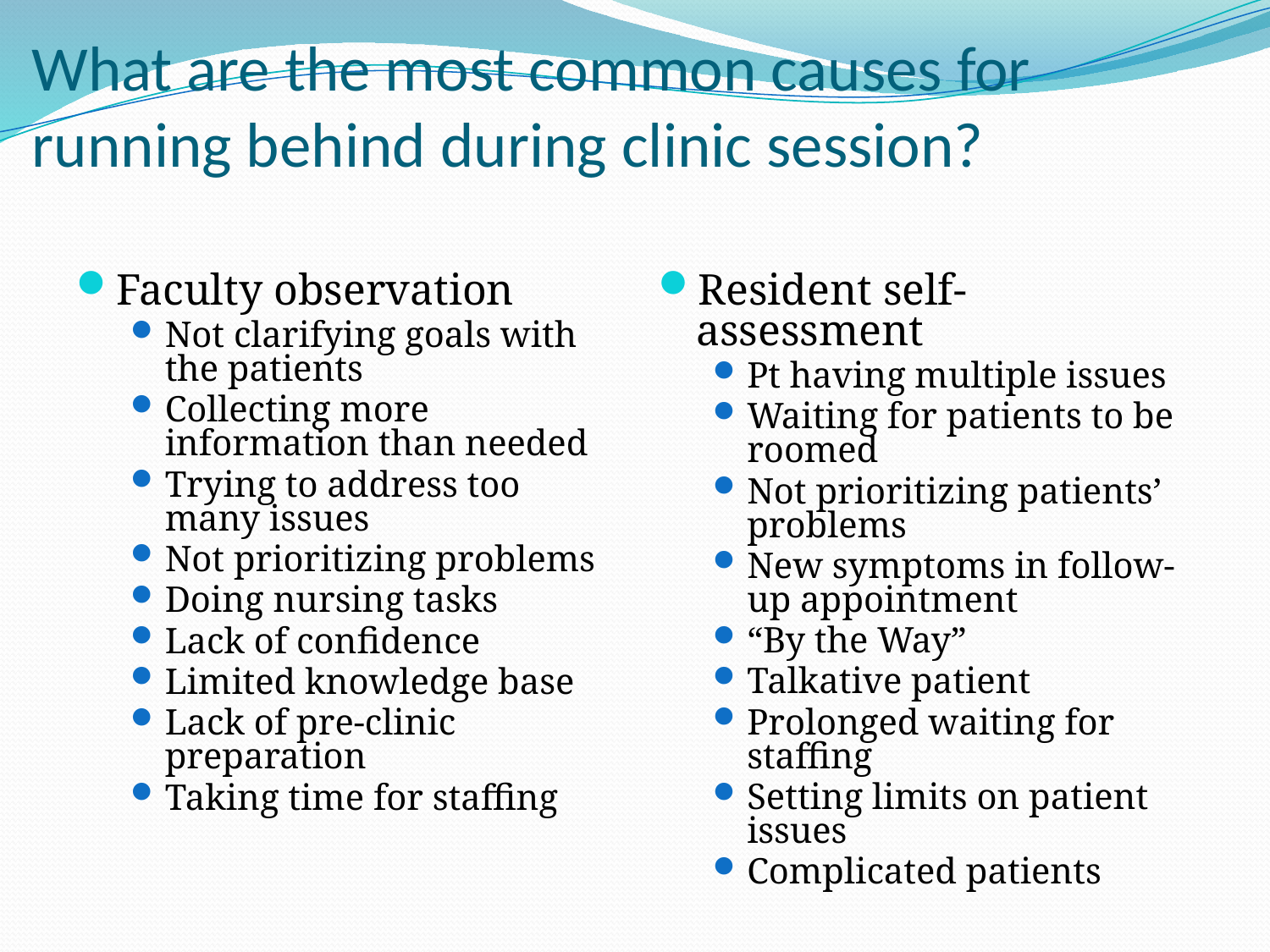

# What are the most common causes for running behind during clinic session?
Faculty observation
Not clarifying goals with the patients
Collecting more information than needed
Trying to address too many issues
Not prioritizing problems
Doing nursing tasks
Lack of confidence
Limited knowledge base
Lack of pre-clinic preparation
Taking time for staffing
Resident self-assessment
Pt having multiple issues
Waiting for patients to be roomed
Not prioritizing patients’ problems
New symptoms in follow-up appointment
“By the Way”
Talkative patient
Prolonged waiting for staffing
Setting limits on patient issues
Complicated patients

## Slide 4
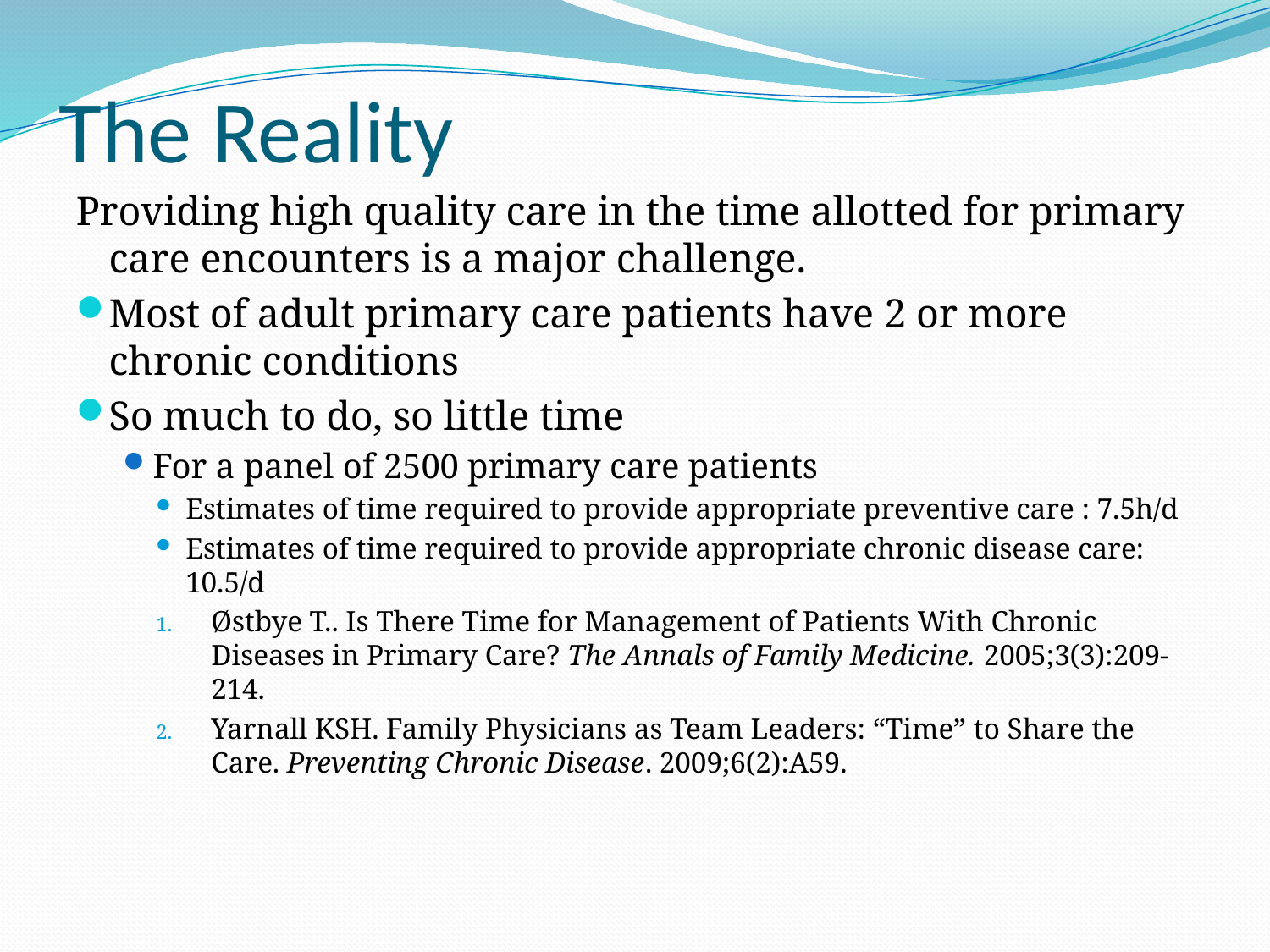

# The Reality
Providing high quality care in the time allotted for primary care encounters is a major challenge.
Most of adult primary care patients have 2 or more chronic conditions
So much to do, so little time
For a panel of 2500 primary care patients
Estimates of time required to provide appropriate preventive care : 7.5h/d
Estimates of time required to provide appropriate chronic disease care: 10.5/d
Østbye T.. Is There Time for Management of Patients With Chronic Diseases in Primary Care? The Annals of Family Medicine. 2005;3(3):209-214.
Yarnall KSH. Family Physicians as Team Leaders: “Time” to Share the Care. Preventing Chronic Disease. 2009;6(2):A59.

## Slide 5
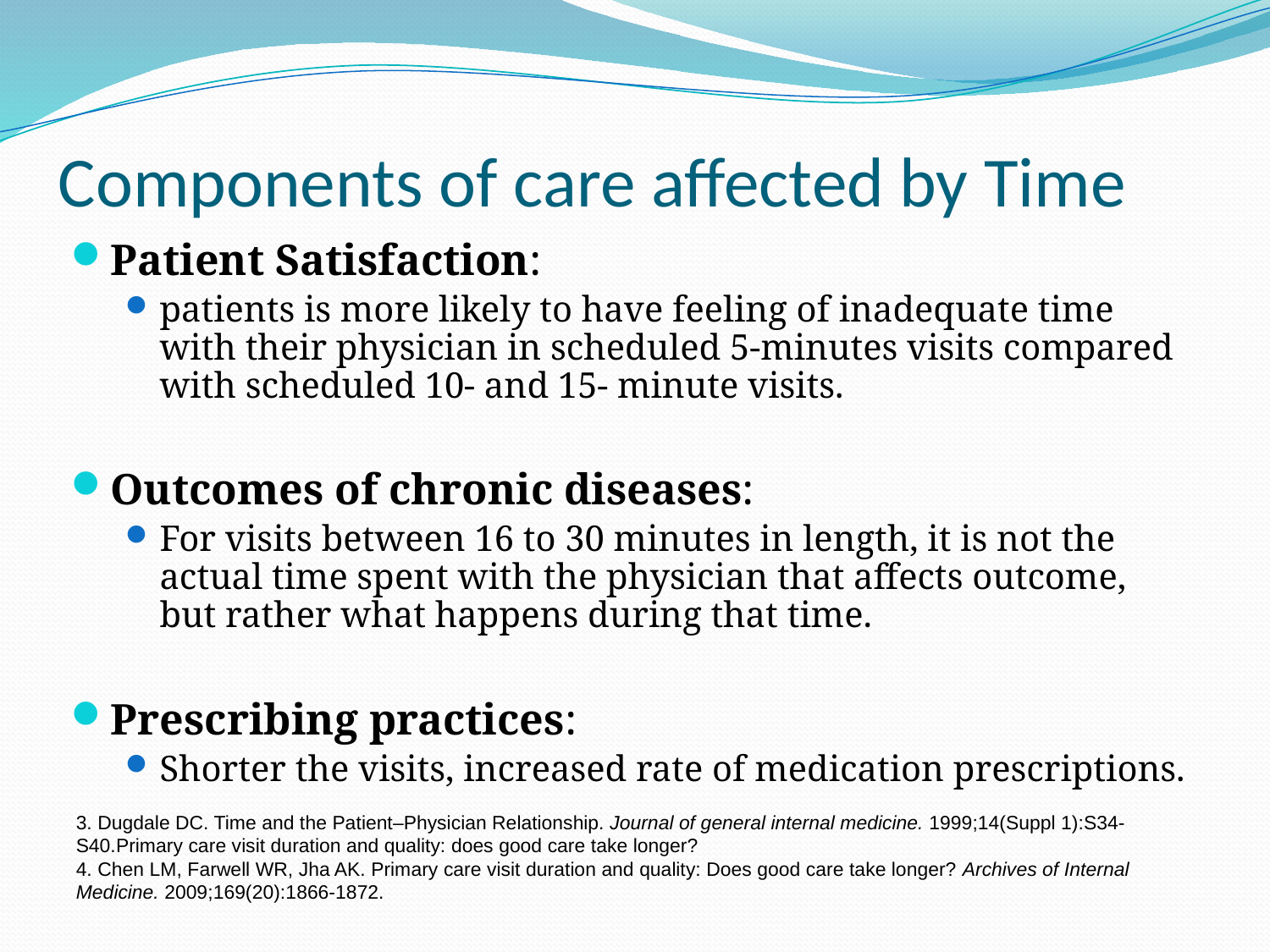

# Components of care affected by Time
Patient Satisfaction:
patients is more likely to have feeling of inadequate time with their physician in scheduled 5-minutes visits compared with scheduled 10- and 15- minute visits.
Outcomes of chronic diseases:
For visits between 16 to 30 minutes in length, it is not the actual time spent with the physician that affects outcome, but rather what happens during that time.
Prescribing practices:
Shorter the visits, increased rate of medication prescriptions.
3. Dugdale DC. Time and the Patient–Physician Relationship. Journal of general internal medicine. 1999;14(Suppl 1):S34-S40.Primary care visit duration and quality: does good care take longer?
4. Chen LM, Farwell WR, Jha AK. Primary care visit duration and quality: Does good care take longer? Archives of Internal Medicine. 2009;169(20):1866-1872.

## Slide 6
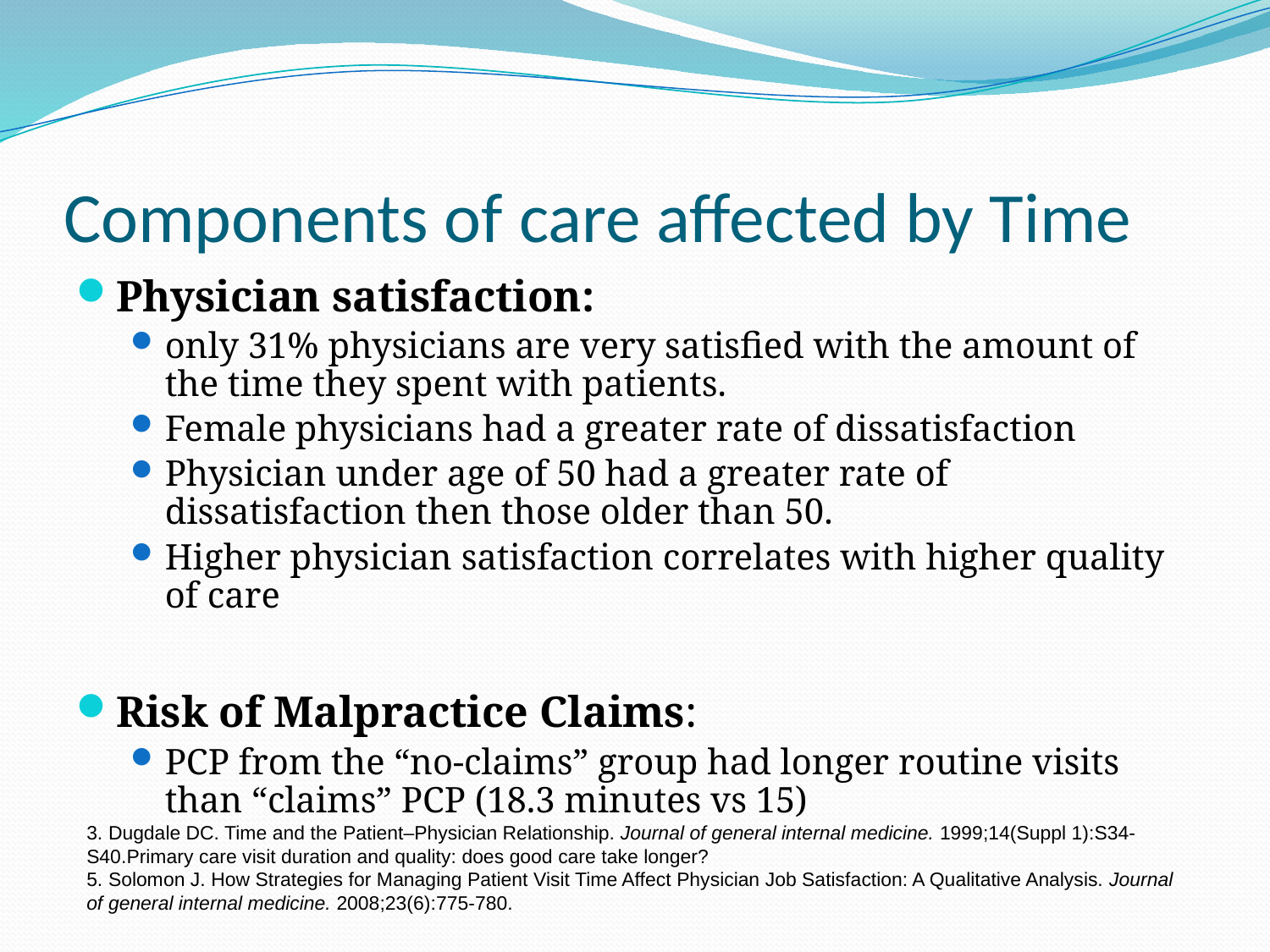

# Components of care affected by Time
Physician satisfaction:
only 31% physicians are very satisfied with the amount of the time they spent with patients.
Female physicians had a greater rate of dissatisfaction
Physician under age of 50 had a greater rate of dissatisfaction then those older than 50.
Higher physician satisfaction correlates with higher quality of care
Risk of Malpractice Claims:
PCP from the “no-claims” group had longer routine visits than “claims” PCP (18.3 minutes vs 15)
3. Dugdale DC. Time and the Patient–Physician Relationship. Journal of general internal medicine. 1999;14(Suppl 1):S34-S40.Primary care visit duration and quality: does good care take longer?
5. Solomon J. How Strategies for Managing Patient Visit Time Affect Physician Job Satisfaction: A Qualitative Analysis. Journal of general internal medicine. 2008;23(6):775-780.

## Slide 7
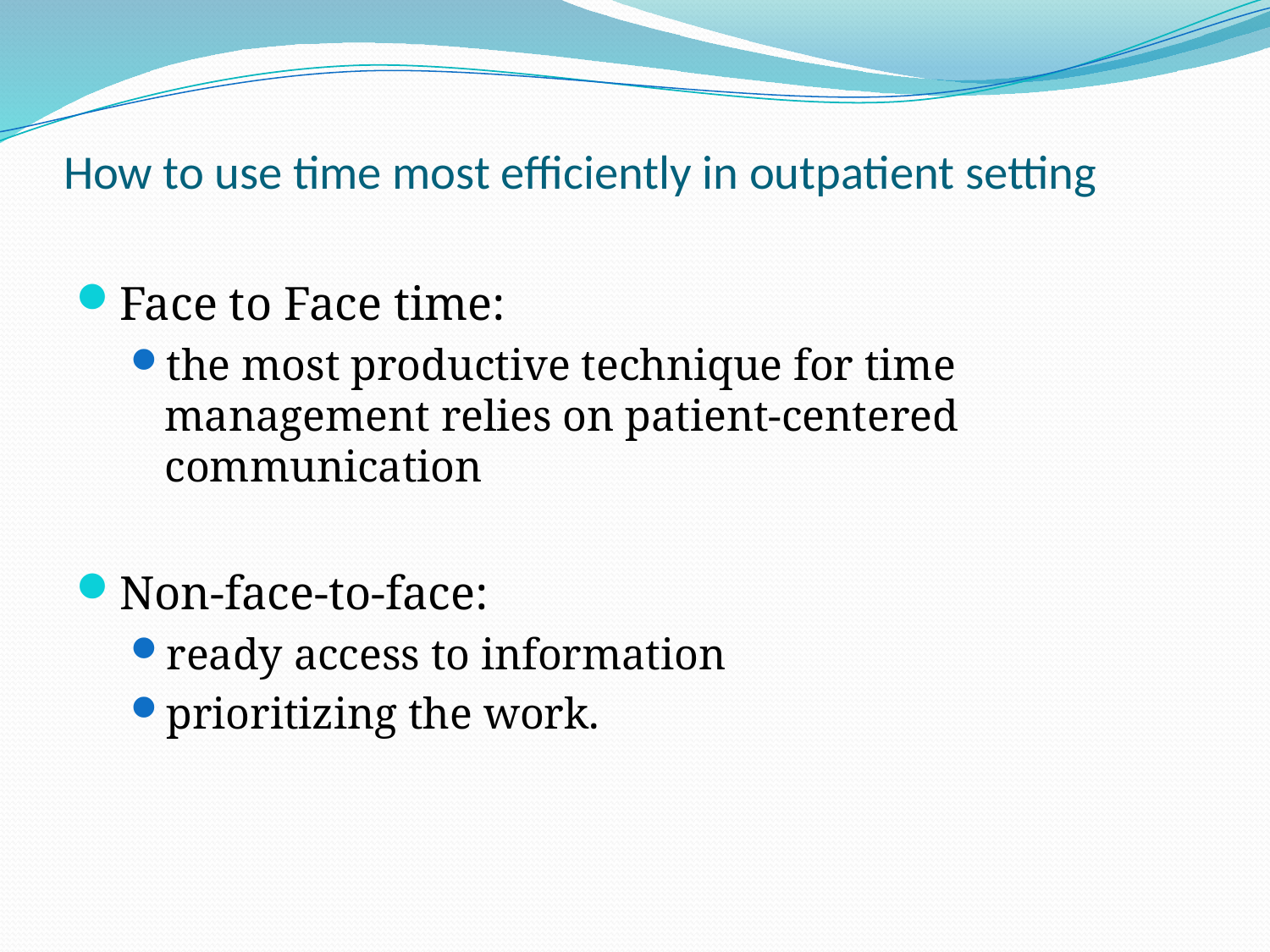

# How to use time most efficiently in outpatient setting
Face to Face time:
the most productive technique for time management relies on patient-centered communication
Non-face-to-face:
ready access to information
prioritizing the work.

## Slide 8
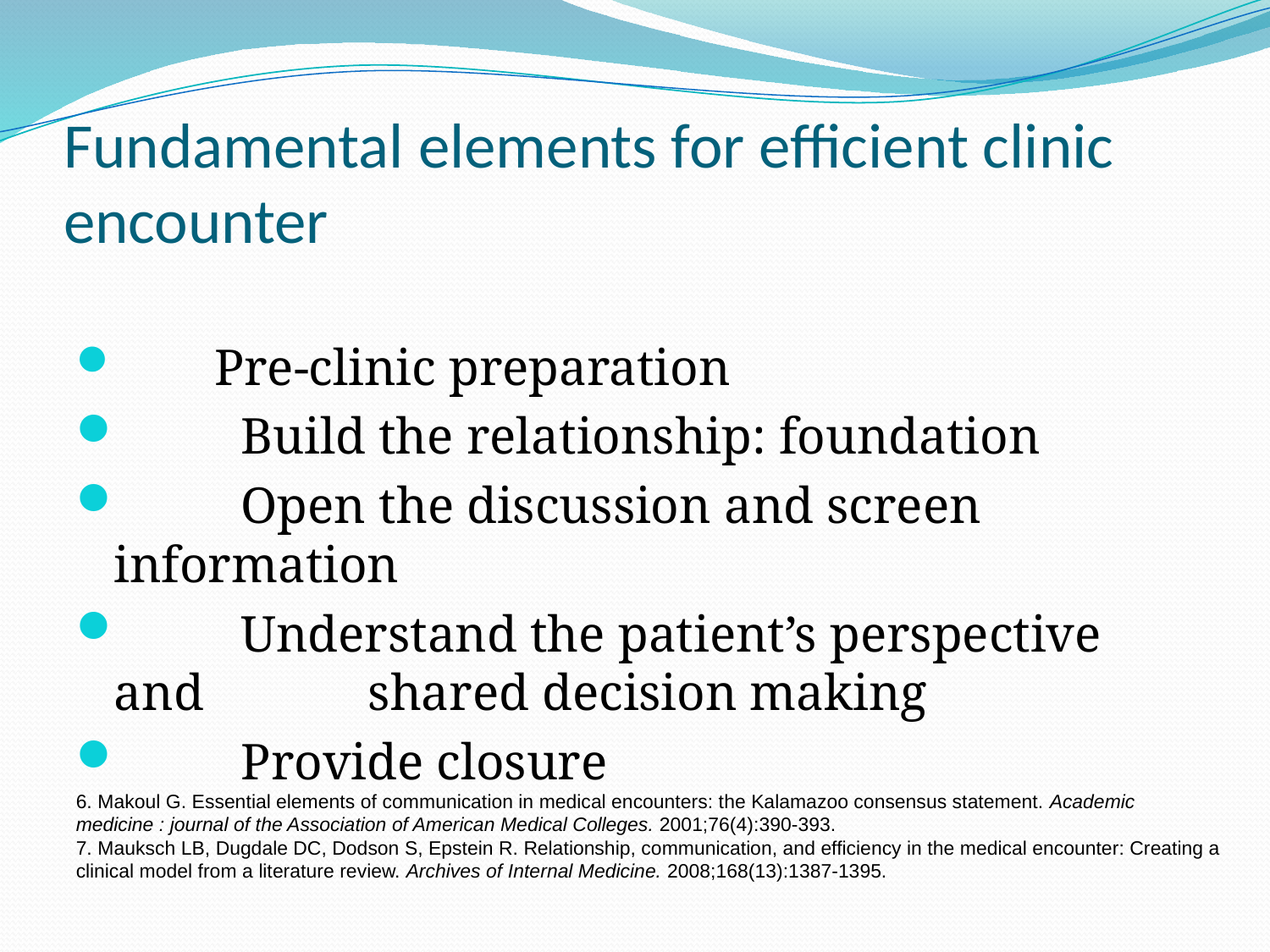

# Fundamental elements for efficient clinic encounter
 Pre-clinic preparation
	Build the relationship: foundation
	Open the discussion and screen information
	Understand the patient’s perspective and 	shared decision making
	Provide closure
6. Makoul G. Essential elements of communication in medical encounters: the Kalamazoo consensus statement. Academic medicine : journal of the Association of American Medical Colleges. 2001;76(4):390-393.
7. Mauksch LB, Dugdale DC, Dodson S, Epstein R. Relationship, communication, and efficiency in the medical encounter: Creating a clinical model from a literature review. Archives of Internal Medicine. 2008;168(13):1387-1395.

## Slide 9
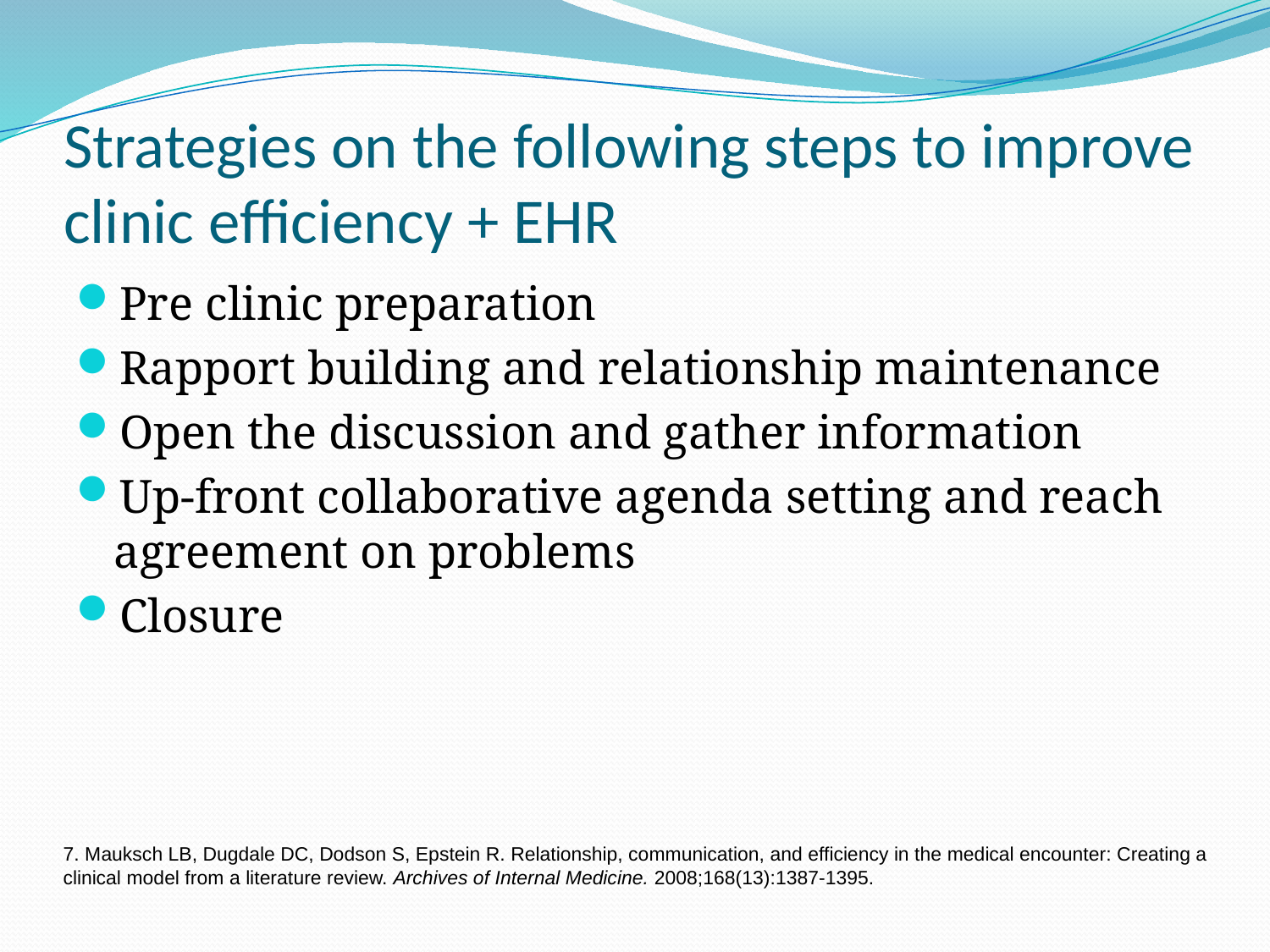

# Strategies on the following steps to improve clinic efficiency + EHR
Pre clinic preparation
Rapport building and relationship maintenance
Open the discussion and gather information
Up-front collaborative agenda setting and reach agreement on problems
Closure
7. Mauksch LB, Dugdale DC, Dodson S, Epstein R. Relationship, communication, and efficiency in the medical encounter: Creating a clinical model from a literature review. Archives of Internal Medicine. 2008;168(13):1387-1395.

## Slide 10
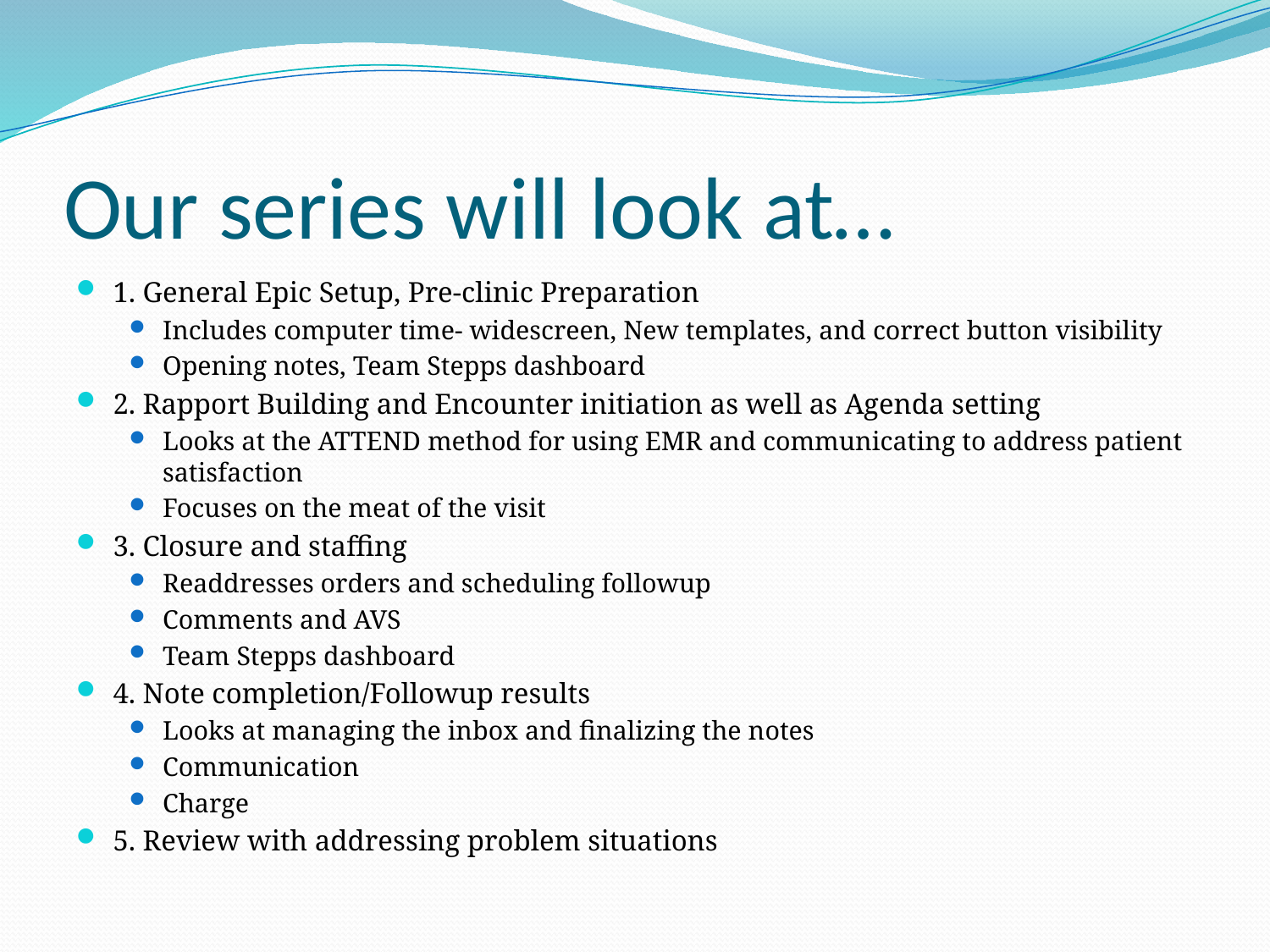

# Our series will look at…
1. General Epic Setup, Pre-clinic Preparation
Includes computer time- widescreen, New templates, and correct button visibility
Opening notes, Team Stepps dashboard
2. Rapport Building and Encounter initiation as well as Agenda setting
Looks at the ATTEND method for using EMR and communicating to address patient satisfaction
Focuses on the meat of the visit
3. Closure and staffing
Readdresses orders and scheduling followup
Comments and AVS
Team Stepps dashboard
4. Note completion/Followup results
Looks at managing the inbox and finalizing the notes
Communication
Charge
5. Review with addressing problem situations

## Slide 11
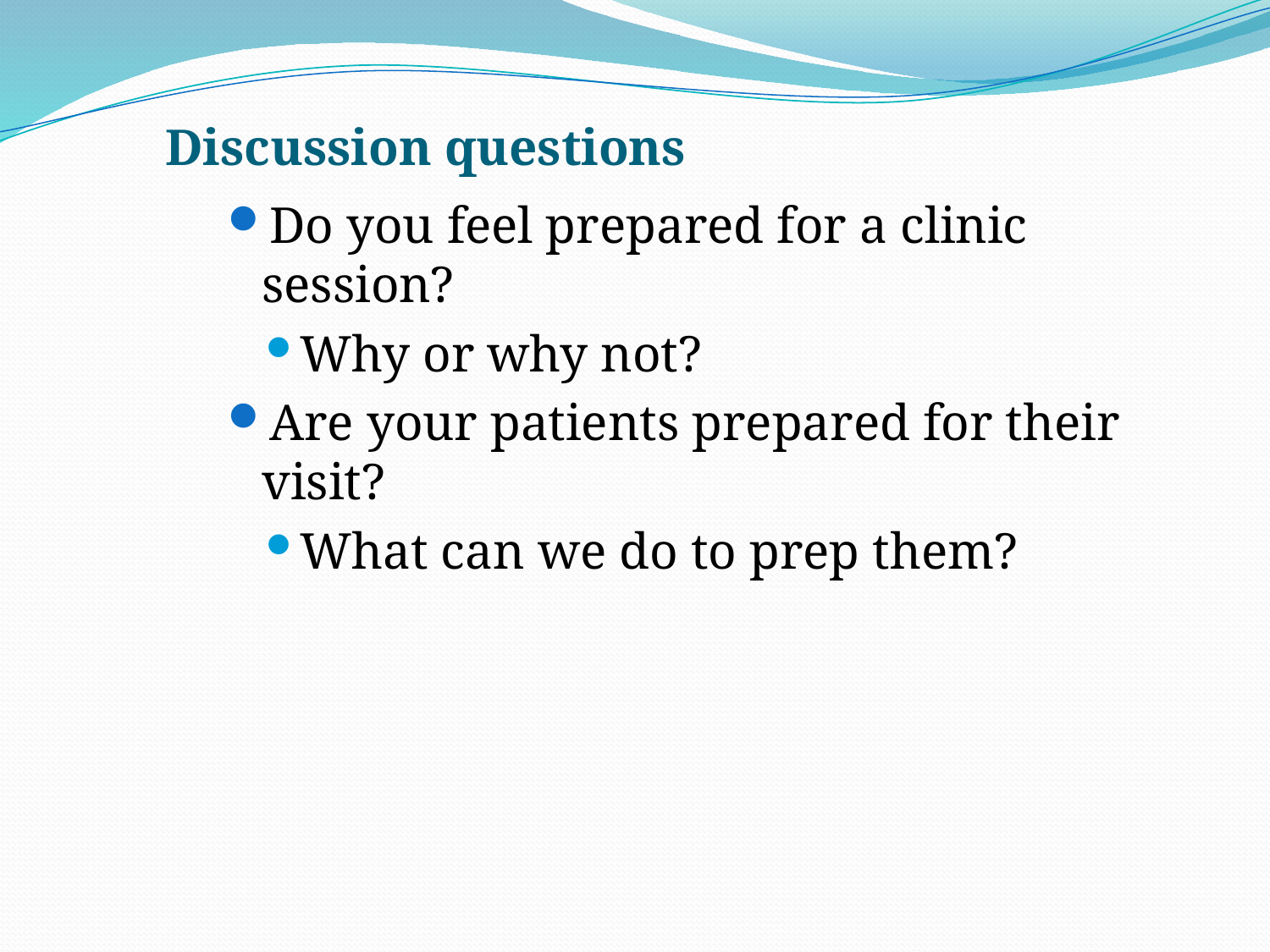

Discussion questions
Do you feel prepared for a clinic session?
Why or why not?
Are your patients prepared for their visit?
What can we do to prep them?

## Slide 12
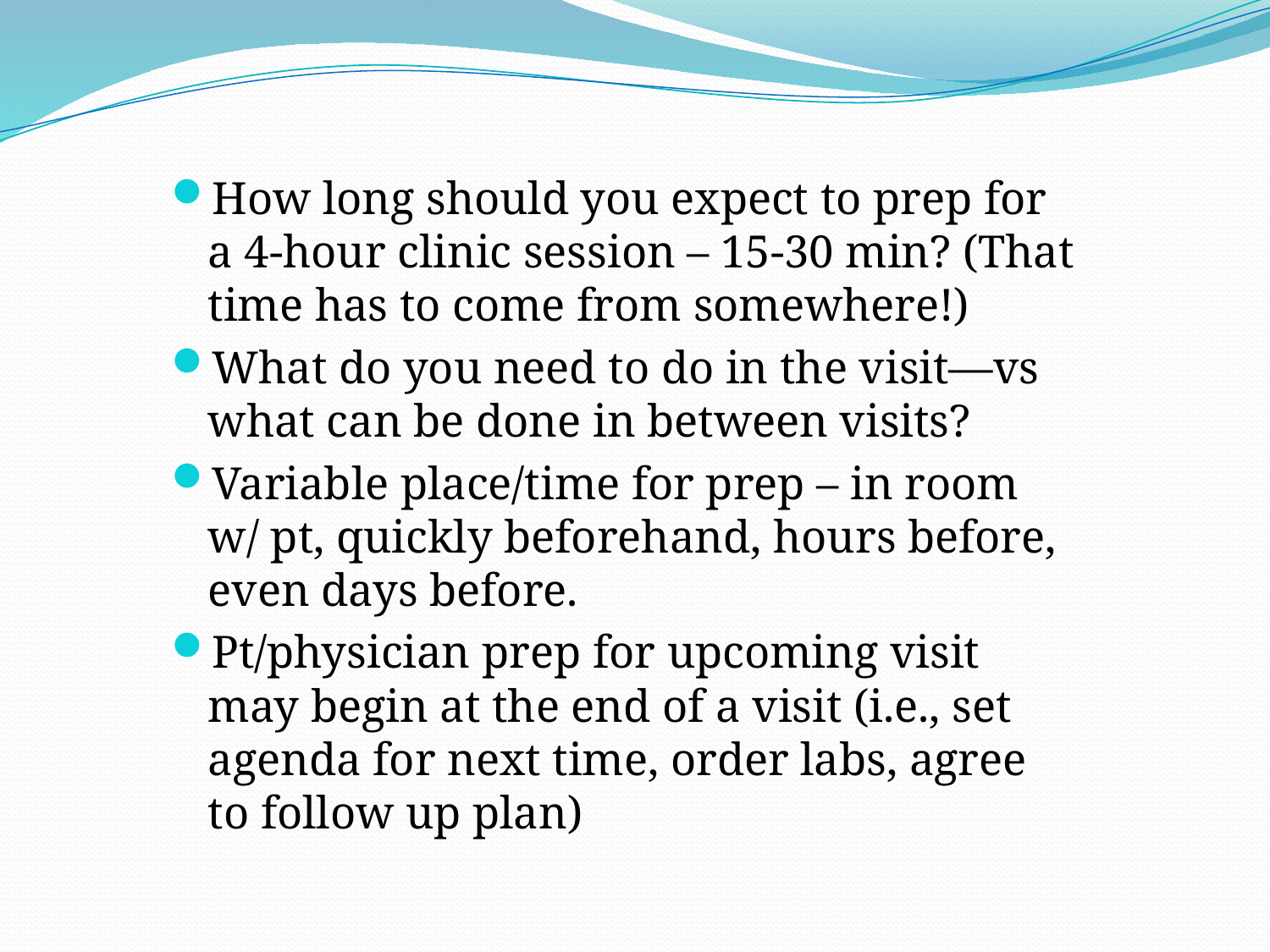

How long should you expect to prep for a 4-hour clinic session – 15-30 min? (That time has to come from somewhere!)
What do you need to do in the visit—vs what can be done in between visits?
Variable place/time for prep – in room w/ pt, quickly beforehand, hours before, even days before.
Pt/physician prep for upcoming visit may begin at the end of a visit (i.e., set agenda for next time, order labs, agree to follow up plan)

## Slide 13
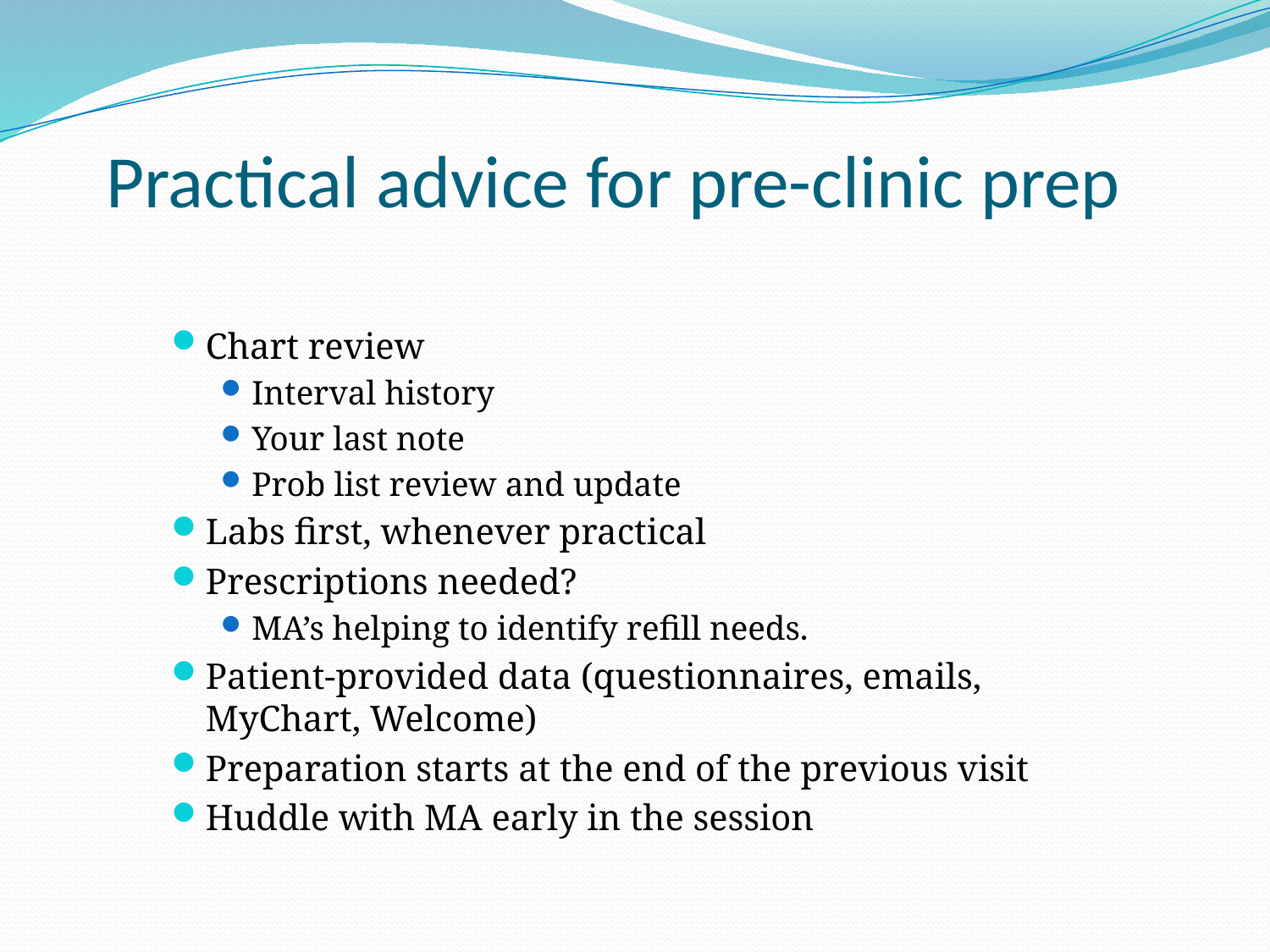

# Practical advice for pre-clinic prep
Chart review
Interval history
Your last note
Prob list review and update
Labs first, whenever practical
Prescriptions needed?
MA’s helping to identify refill needs.
Patient-provided data (questionnaires, emails, MyChart, Welcome)
Preparation starts at the end of the previous visit
Huddle with MA early in the session

## Slide 14
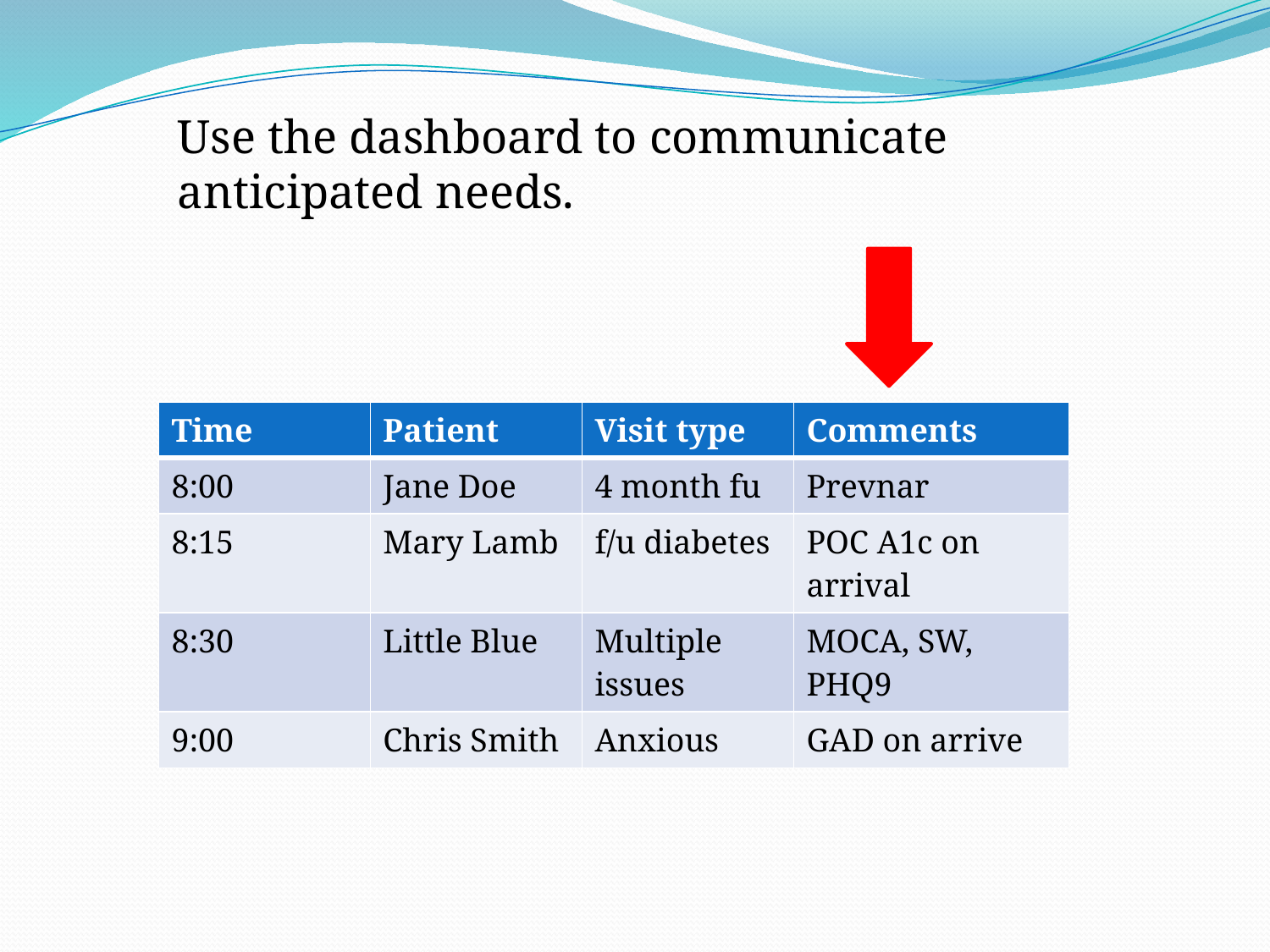

Use the dashboard to communicate anticipated needs.
| Time | Patient | Visit type | Comments |
| --- | --- | --- | --- |
| 8:00 | Jane Doe | 4 month fu | Prevnar |
| 8:15 | Mary Lamb | f/u diabetes | POC A1c on arrival |
| 8:30 | Little Blue | Multiple issues | MOCA, SW, PHQ9 |
| 9:00 | Chris Smith | Anxious | GAD on arrive |

## Slide 15
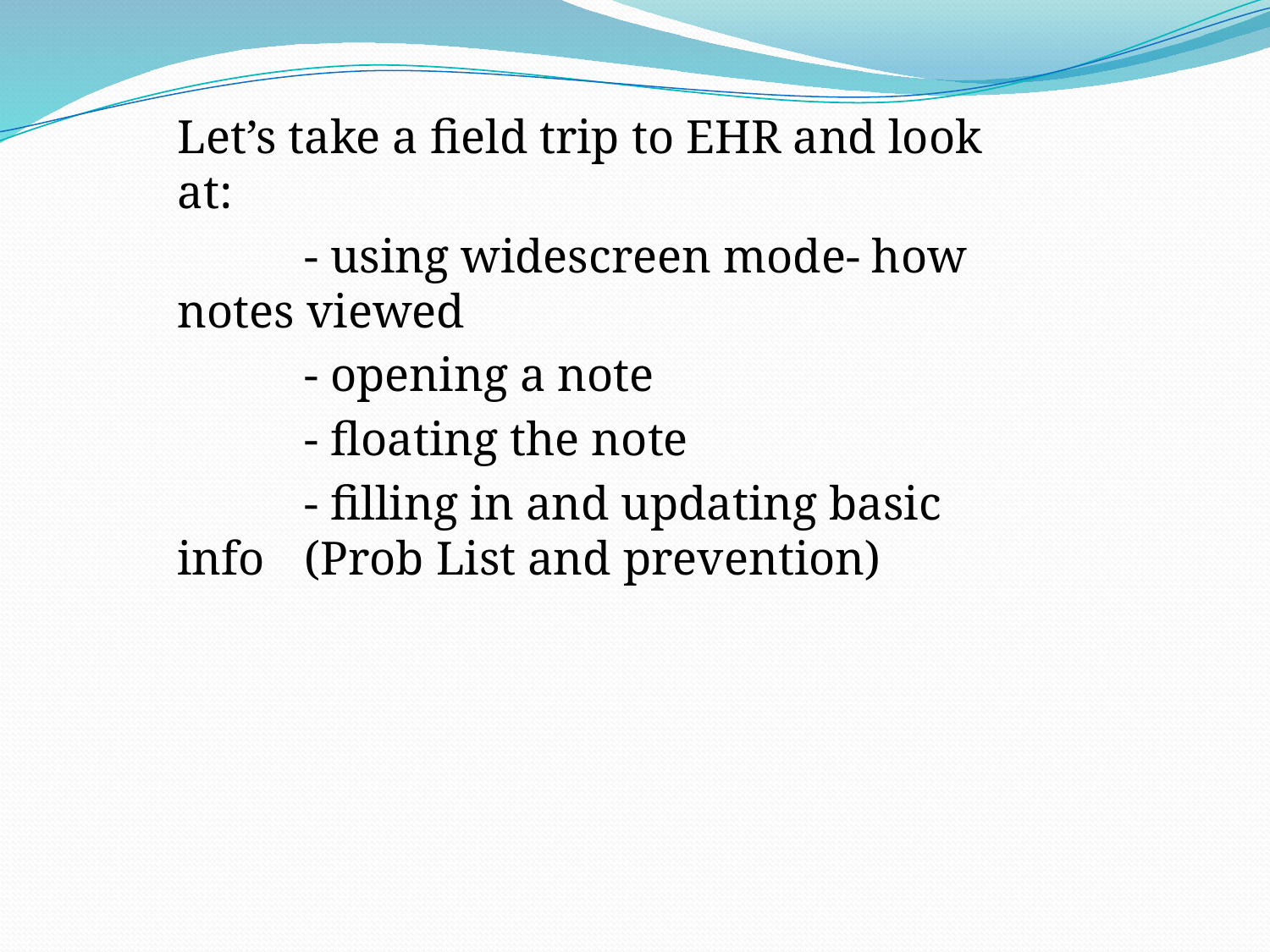

Let’s take a field trip to EHR and look at:
	- using widescreen mode- how notes viewed
	- opening a note
	- floating the note
	- filling in and updating basic info 	(Prob List and prevention)

## Slide 16
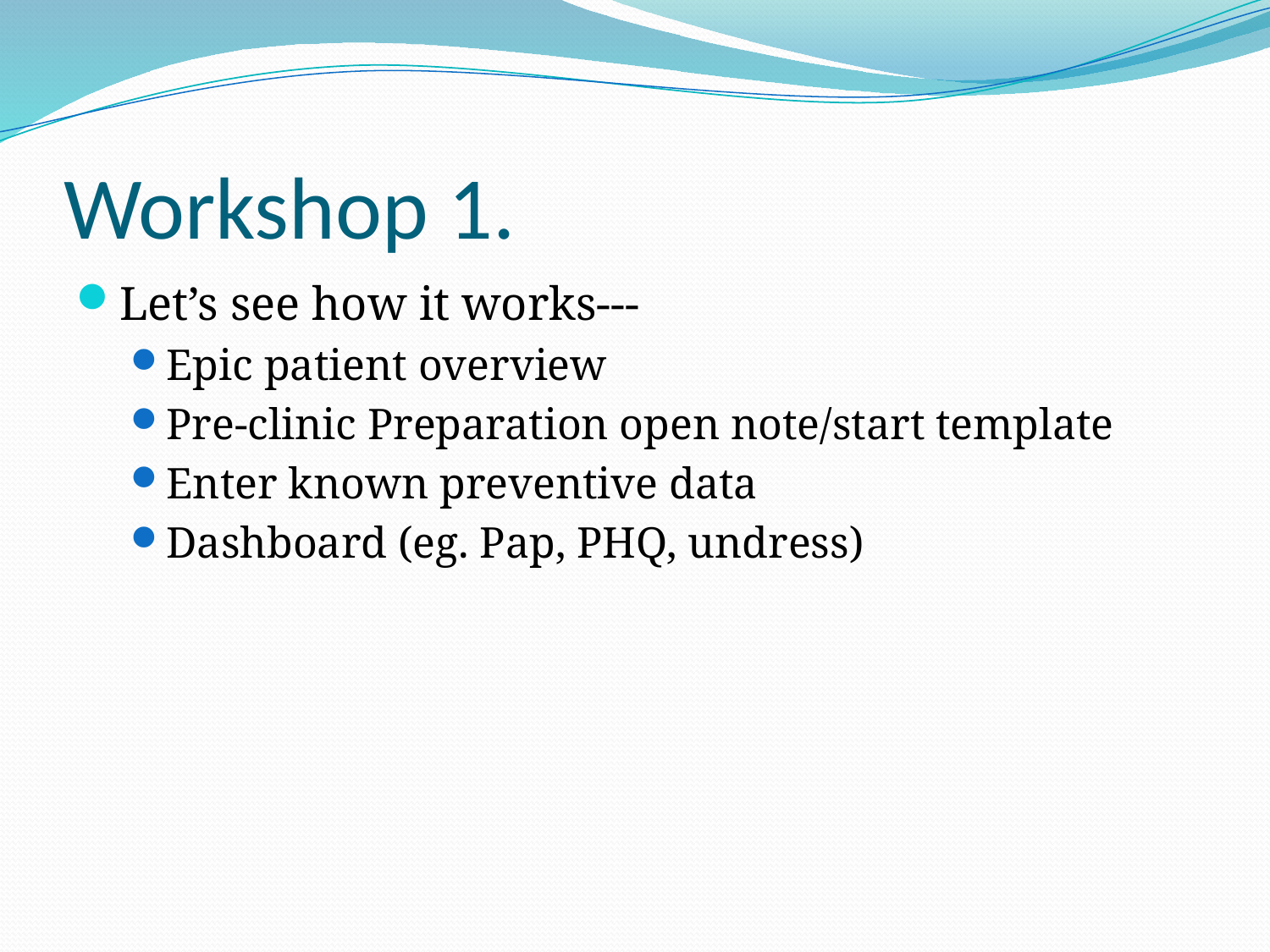

# Workshop 1.
Let’s see how it works---
Epic patient overview
Pre-clinic Preparation open note/start template
Enter known preventive data
Dashboard (eg. Pap, PHQ, undress)

## Slide 17
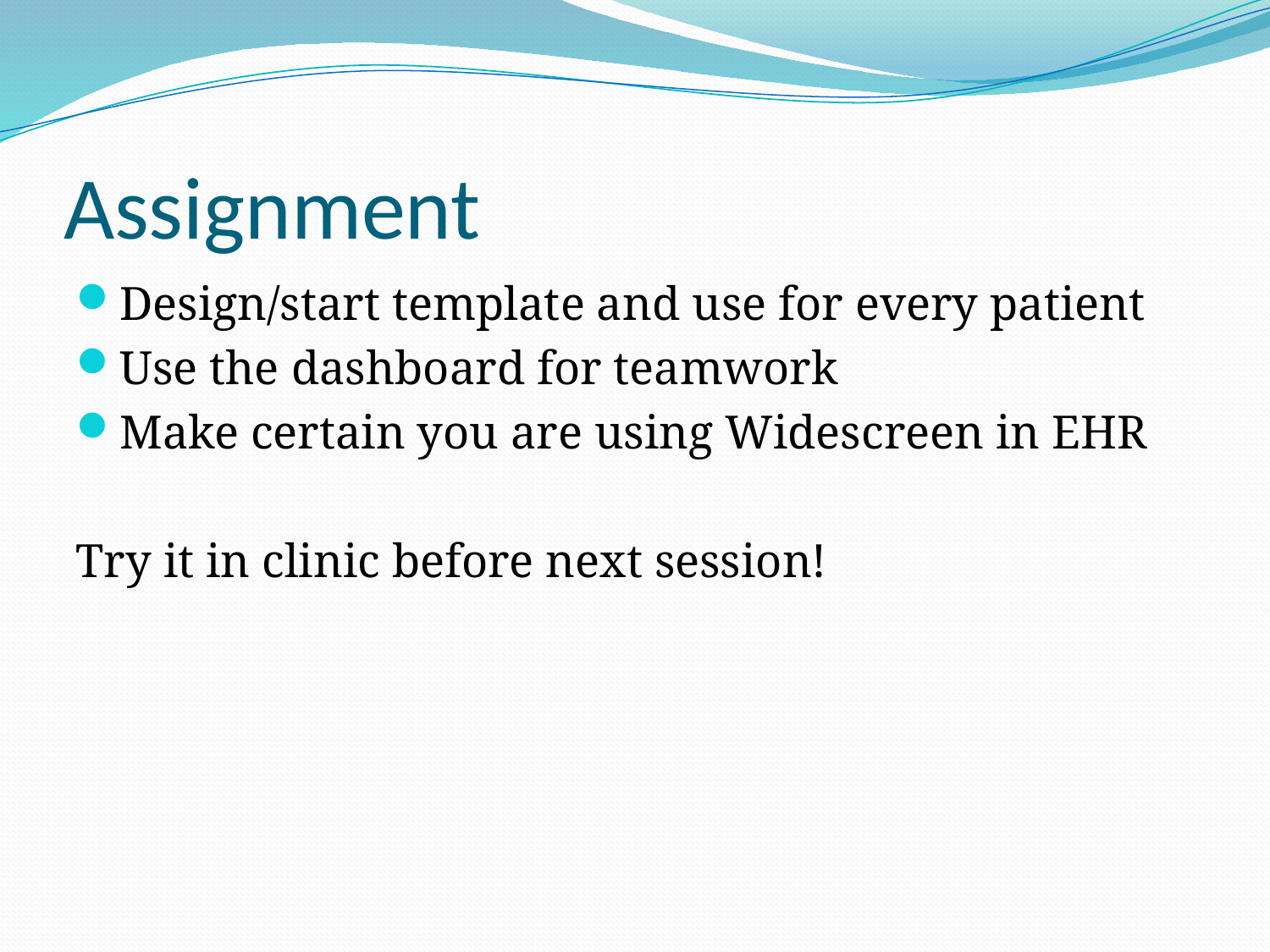

# Assignment
Design/start template and use for every patient
Use the dashboard for teamwork
Make certain you are using Widescreen in EHR
Try it in clinic before next session!

## Slide 18
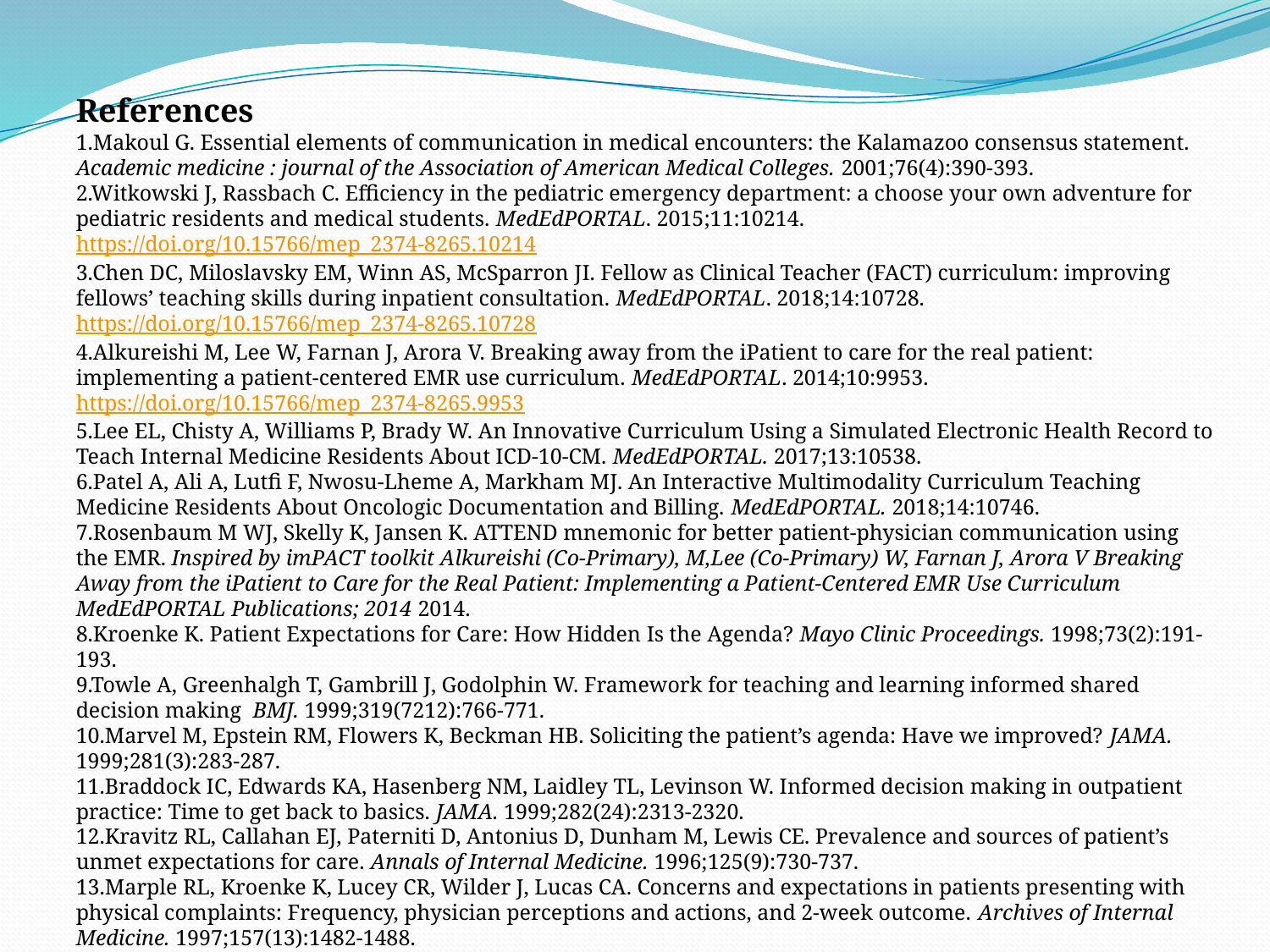

References
1.Makoul G. Essential elements of communication in medical encounters: the Kalamazoo consensus statement. Academic medicine : journal of the Association of American Medical Colleges. 2001;76(4):390-393.
2.Witkowski J, Rassbach C. Efficiency in the pediatric emergency department: a choose your own adventure for pediatric residents and medical students. MedEdPORTAL. 2015;11:10214. https://doi.org/10.15766/mep_2374-8265.10214
3.Chen DC, Miloslavsky EM, Winn AS, McSparron JI. Fellow as Clinical Teacher (FACT) curriculum: improving fellows’ teaching skills during inpatient consultation. MedEdPORTAL. 2018;14:10728. https://doi.org/10.15766/mep_2374-8265.10728
4.Alkureishi M, Lee W, Farnan J, Arora V. Breaking away from the iPatient to care for the real patient: implementing a patient-centered EMR use curriculum. MedEdPORTAL. 2014;10:9953. https://doi.org/10.15766/mep_2374-8265.9953
5.Lee EL, Chisty A, Williams P, Brady W. An Innovative Curriculum Using a Simulated Electronic Health Record to Teach Internal Medicine Residents About ICD-10-CM. MedEdPORTAL. 2017;13:10538.
6.Patel A, Ali A, Lutfi F, Nwosu-Lheme A, Markham MJ. An Interactive Multimodality Curriculum Teaching Medicine Residents About Oncologic Documentation and Billing. MedEdPORTAL. 2018;14:10746.
7.Rosenbaum M WJ, Skelly K, Jansen K. ATTEND mnemonic for better patient-physician communication using the EMR. Inspired by imPACT toolkit Alkureishi (Co-Primary), M,Lee (Co-Primary) W, Farnan J, Arora V Breaking Away from the iPatient to Care for the Real Patient: Implementing a Patient-Centered EMR Use Curriculum MedEdPORTAL Publications; 2014 2014.
8.Kroenke K. Patient Expectations for Care: How Hidden Is the Agenda? Mayo Clinic Proceedings. 1998;73(2):191-193.
9.Towle A, Greenhalgh T, Gambrill J, Godolphin W. Framework for teaching and learning informed shared decision making BMJ. 1999;319(7212):766-771.
10.Marvel M, Epstein RM, Flowers K, Beckman HB. Soliciting the patient’s agenda: Have we improved? JAMA. 1999;281(3):283-287.
11.Braddock IC, Edwards KA, Hasenberg NM, Laidley TL, Levinson W. Informed decision making in outpatient practice: Time to get back to basics. JAMA. 1999;282(24):2313-2320.
12.Kravitz RL, Callahan EJ, Paterniti D, Antonius D, Dunham M, Lewis CE. Prevalence and sources of patient’s unmet expectations for care. Annals of Internal Medicine. 1996;125(9):730-737.
13.Marple RL, Kroenke K, Lucey CR, Wilder J, Lucas CA. Concerns and expectations in patients presenting with physical complaints: Frequency, physician perceptions and actions, and 2-week outcome. Archives of Internal Medicine. 1997;157(13):1482-1488.
14.Mauksch L, Hillenburg L, Robins L. The Establishing Focus protocol: Training for collaborative agenda setting and time management in the medical interview. Vol 192001.
15.Østbye T, Yarnall KSH, Krause KM, Pollak KI, Gradison M, Michener JL. Is There Time for Management of Patients With Chronic Diseases in Primary Care? The Annals of Family Medicine. 2005;3(3):209-214.

## Slide 19
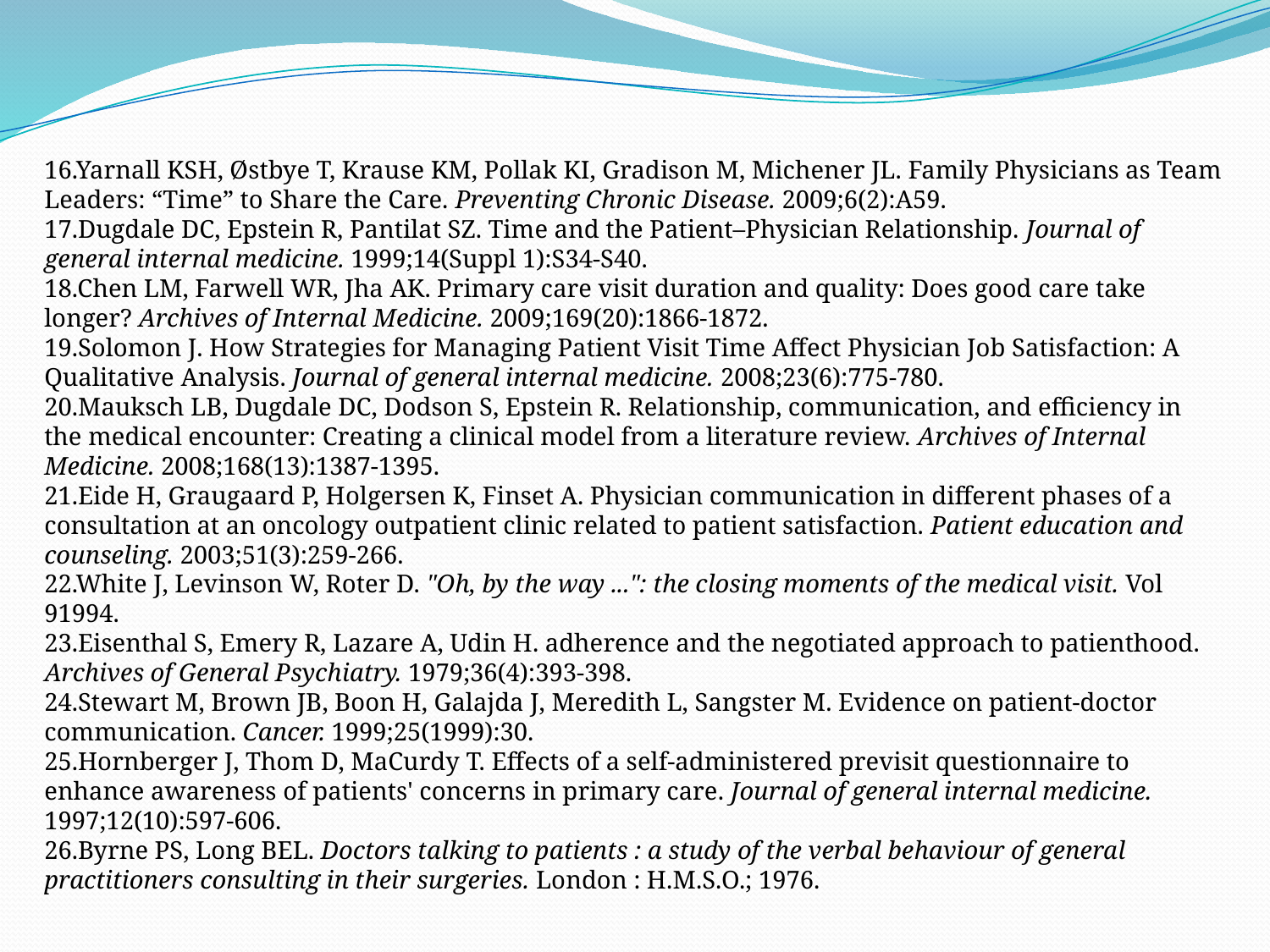

16.Yarnall KSH, Østbye T, Krause KM, Pollak KI, Gradison M, Michener JL. Family Physicians as Team Leaders: “Time” to Share the Care. Preventing Chronic Disease. 2009;6(2):A59.
17.Dugdale DC, Epstein R, Pantilat SZ. Time and the Patient–Physician Relationship. Journal of general internal medicine. 1999;14(Suppl 1):S34-S40.
18.Chen LM, Farwell WR, Jha AK. Primary care visit duration and quality: Does good care take longer? Archives of Internal Medicine. 2009;169(20):1866-1872.
19.Solomon J. How Strategies for Managing Patient Visit Time Affect Physician Job Satisfaction: A Qualitative Analysis. Journal of general internal medicine. 2008;23(6):775-780.
20.Mauksch LB, Dugdale DC, Dodson S, Epstein R. Relationship, communication, and efficiency in the medical encounter: Creating a clinical model from a literature review. Archives of Internal Medicine. 2008;168(13):1387-1395.
21.Eide H, Graugaard P, Holgersen K, Finset A. Physician communication in different phases of a consultation at an oncology outpatient clinic related to patient satisfaction. Patient education and counseling. 2003;51(3):259-266.
22.White J, Levinson W, Roter D. "Oh, by the way ...": the closing moments of the medical visit. Vol 91994.
23.Eisenthal S, Emery R, Lazare A, Udin H. adherence and the negotiated approach to patienthood. Archives of General Psychiatry. 1979;36(4):393-398.
24.Stewart M, Brown JB, Boon H, Galajda J, Meredith L, Sangster M. Evidence on patient-doctor communication. Cancer. 1999;25(1999):30.
25.Hornberger J, Thom D, MaCurdy T. Effects of a self-administered previsit questionnaire to enhance awareness of patients' concerns in primary care. Journal of general internal medicine. 1997;12(10):597-606.
26.Byrne PS, Long BEL. Doctors talking to patients : a study of the verbal behaviour of general practitioners consulting in their surgeries. London : H.M.S.O.; 1976.
