# Supplementary material for: A Curriculum for Teaching Clinical Efficiency Focusing on Specific Communication Skills While Maximizing the Electronic Health Record
Source: MedEdPORTAL. 2020 Oct 29;16:10989. doi: 10.15766/mep_2374-8265.10989 (PMC7597939; doi:10.15766/mep_2374-8265.10989)
Supplement: Supplementary file 1 — Efficiency Preworkshop Needs Assessment Survey.docxWorkshop 1 - Setting up the Template and Working in EHR.pptxSample Clinic Note and AVS Template.docxWorkshop 2 - Preclinic Preparation and Rapport Building.pptxEfficiency ATTEND Practice Card.docxWorkshop 3 - Agenda Setting and Relationship Maintenance.pptxEfficiency Agenda Setting Practice.docxWorkshop 4 - Visit Closure.pptxEfficiency Closure Card and Cases.docxEfficiency Postworkshop Evaluation.docx [file mep_2374-8265.10989-s001.zip › C. Sample Clinic Note and AVS Template.docx]

**Appendix C: Clinic Note and AVS Templates**

**Clinic Note**

**Encounter Date: @ENCOUNTERDATE@**

**@APPTNOTE@**

| **Subjective:** |
| --- |

@CC@

**History of Present Illness:**

@NAME@ is a @AGE@ @SEX@

| **Objective:** |
| --- |

@VS@

General: Alert in NAD. Vitals as documented.

Skin: without lesions.

HEENT: conjunctiva clear, TMs pearly grey with sharp light reflex, clear EACs, oropharynx is pink and moist without erythema or exudate

Neck: supple without lymphadenopathy or thyromegaly

Heart: regular rate and ryhthm without murmur

Lungs: BCTA

Abdomen: soft, nontender, without masses

Musculoskeletal: Full ROM

Psych: alert and oriented x 3, affect full, no suicidal thought or ideation

@ENCRESULTS@

| **Assessment and Plan:** |
| --- |

@AGE@ @SEX@

@DIAGNOSISWITHCOMMENTS@

@PATINSTROP@

@A743@

**AVS Template:**

You saw @ME@ in clinic today. Thanks for coming!

@DIAGNOSISWITHCOMMENTS@

@FOLLOWUPORDER@

-seatbelts 100% of the time.

1. As always, if you need you may call the *University of Iowa Hospitals and Clinics Family Medicine Clinic* at any time at 319-384-7222 --you can leave a message with the nurses and they will get it to me. Or even better, you can send a note to me via mychart and I'll get it right away!
2. For a *prescription refill*, you may call 319-384-7222. Please allow 3 days to process prescriptions.
3. If we didn't get to all your concerns today, we will again start with that list at your next visit and continue to make sure we continue to work on addressing all your concerns.
