# Supplementary material for: A Curriculum for Teaching Clinical Efficiency Focusing on Specific Communication Skills While Maximizing the Electronic Health Record
Source: MedEdPORTAL. 2020 Oct 29;16:10989. doi: 10.15766/mep_2374-8265.10989 (PMC7597939; doi:10.15766/mep_2374-8265.10989)
Supplement: Supplementary file 1 — Efficiency Preworkshop Needs Assessment Survey.docxWorkshop 1 - Setting up the Template and Working in EHR.pptxSample Clinic Note and AVS Template.docxWorkshop 2 - Preclinic Preparation and Rapport Building.pptxEfficiency ATTEND Practice Card.docxWorkshop 3 - Agenda Setting and Relationship Maintenance.pptxEfficiency Agenda Setting Practice.docxWorkshop 4 - Visit Closure.pptxEfficiency Closure Card and Cases.docxEfficiency Postworkshop Evaluation.docx [file mep_2374-8265.10989-s001.zip › D. Workshop 2 - Preclinic Preparation and Rapport Building.pptx]

## Slide 1
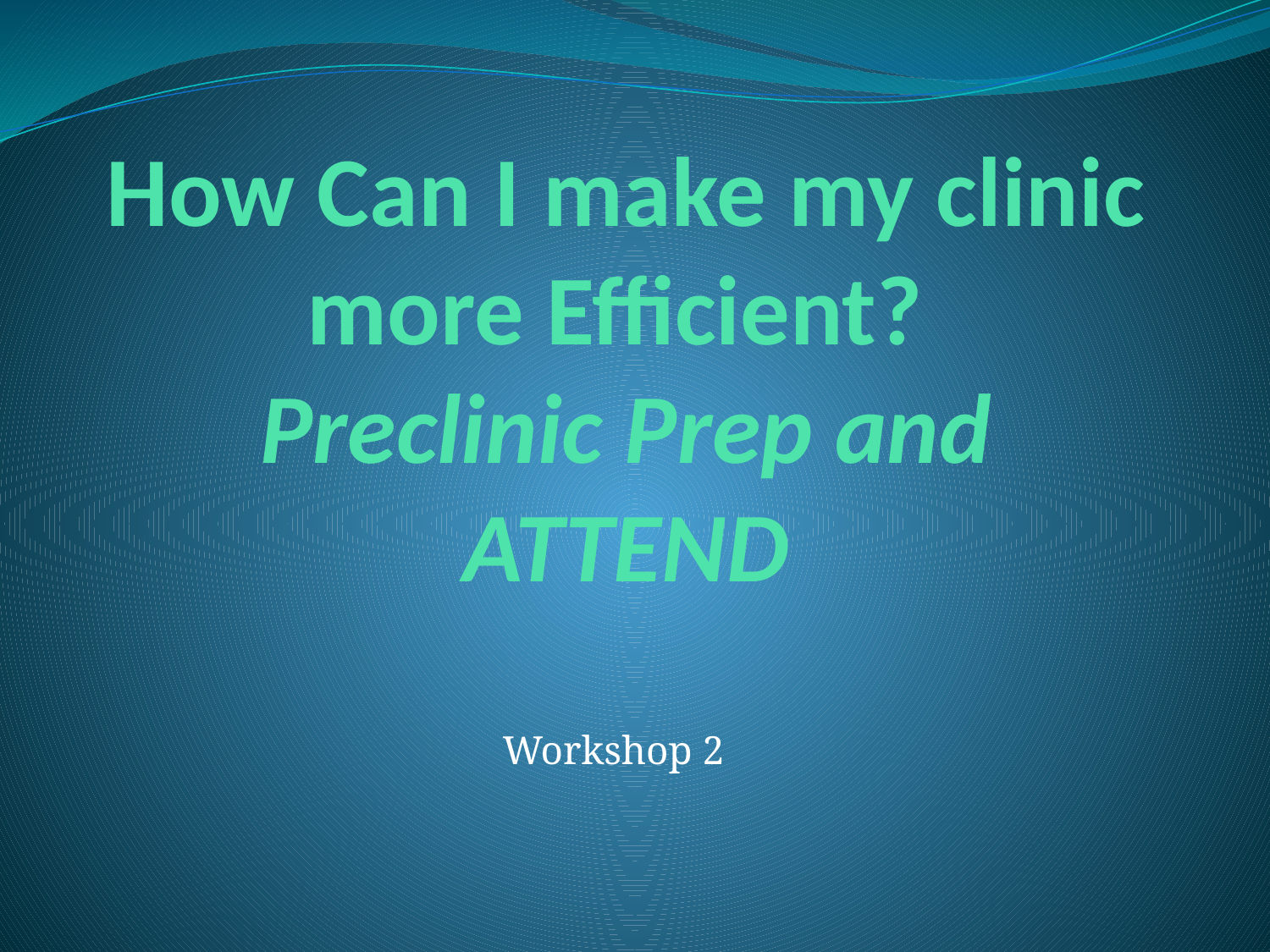

# How Can I make my clinic more Efficient? Preclinic Prep and ATTEND
Workshop 2

## Slide 2
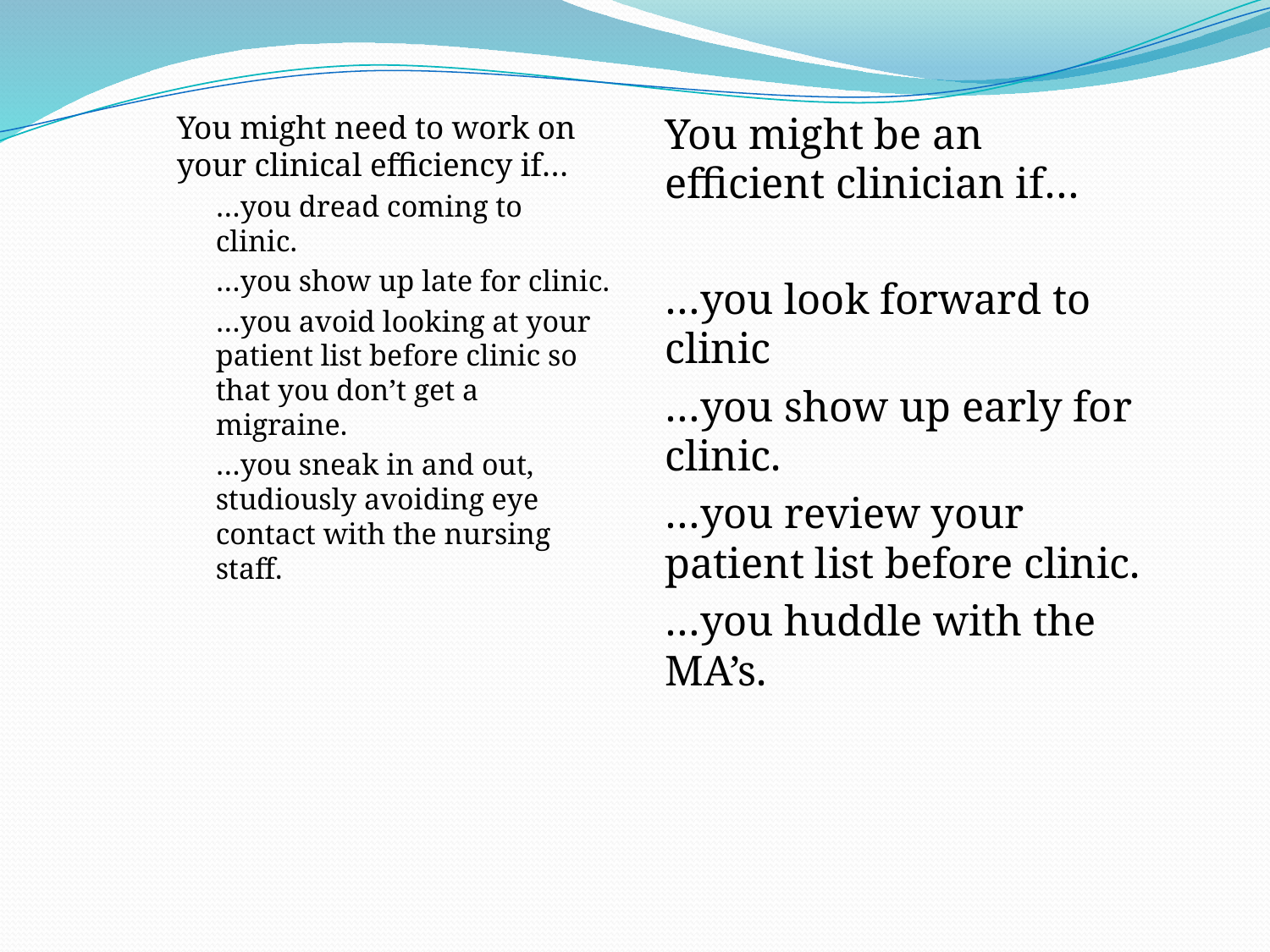

You might need to work on your clinical efficiency if…
…you dread coming to clinic.
…you show up late for clinic.
…you avoid looking at your patient list before clinic so that you don’t get a migraine.
…you sneak in and out, studiously avoiding eye contact with the nursing staff.
You might be an efficient clinician if…
…you look forward to clinic
…you show up early for clinic.
…you review your patient list before clinic.
…you huddle with the MA’s.

## Slide 3
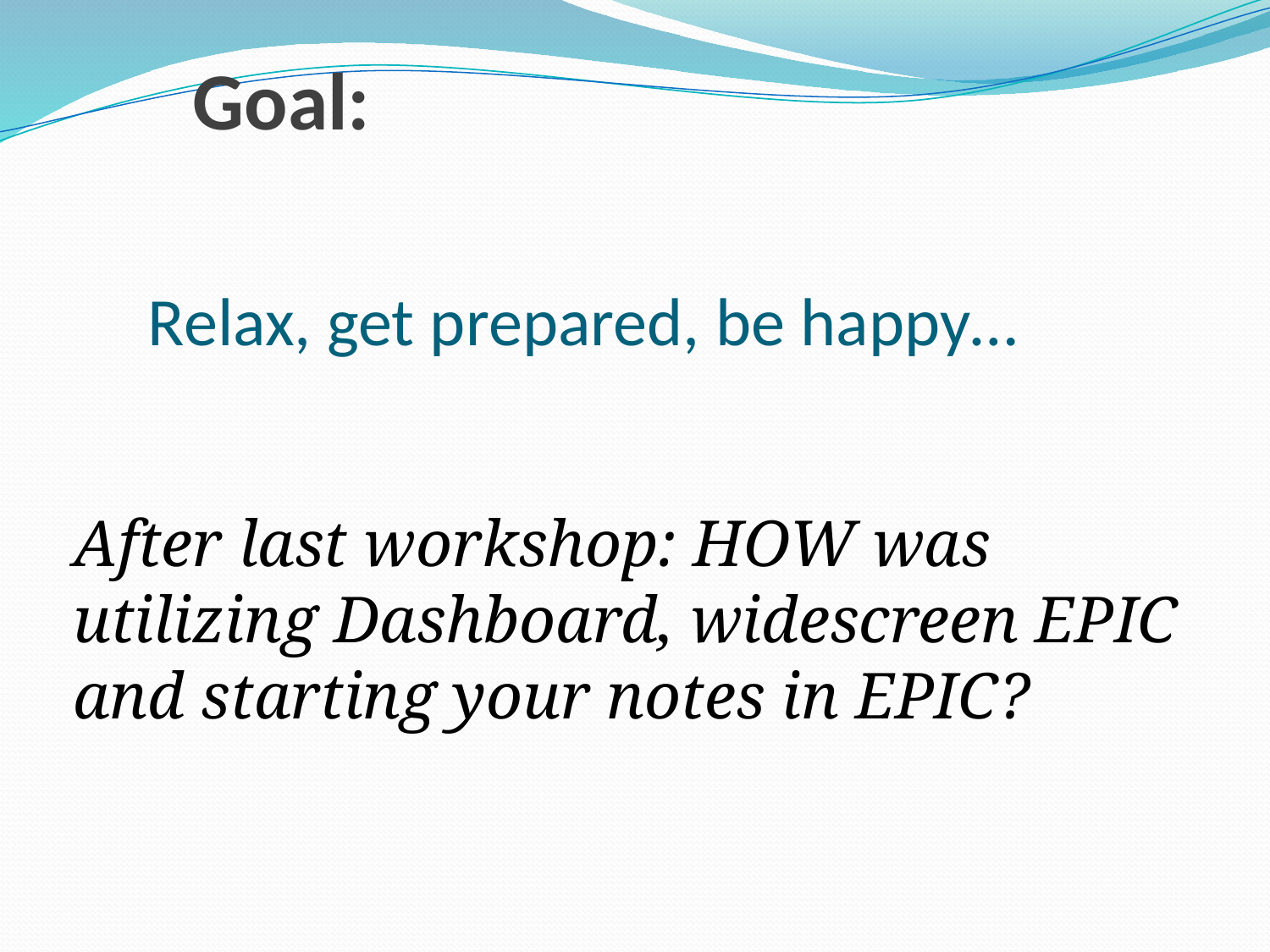

Goal:
# Relax, get prepared, be happy…
After last workshop: HOW was utilizing Dashboard, widescreen EPIC and starting your notes in EPIC?

## Slide 4
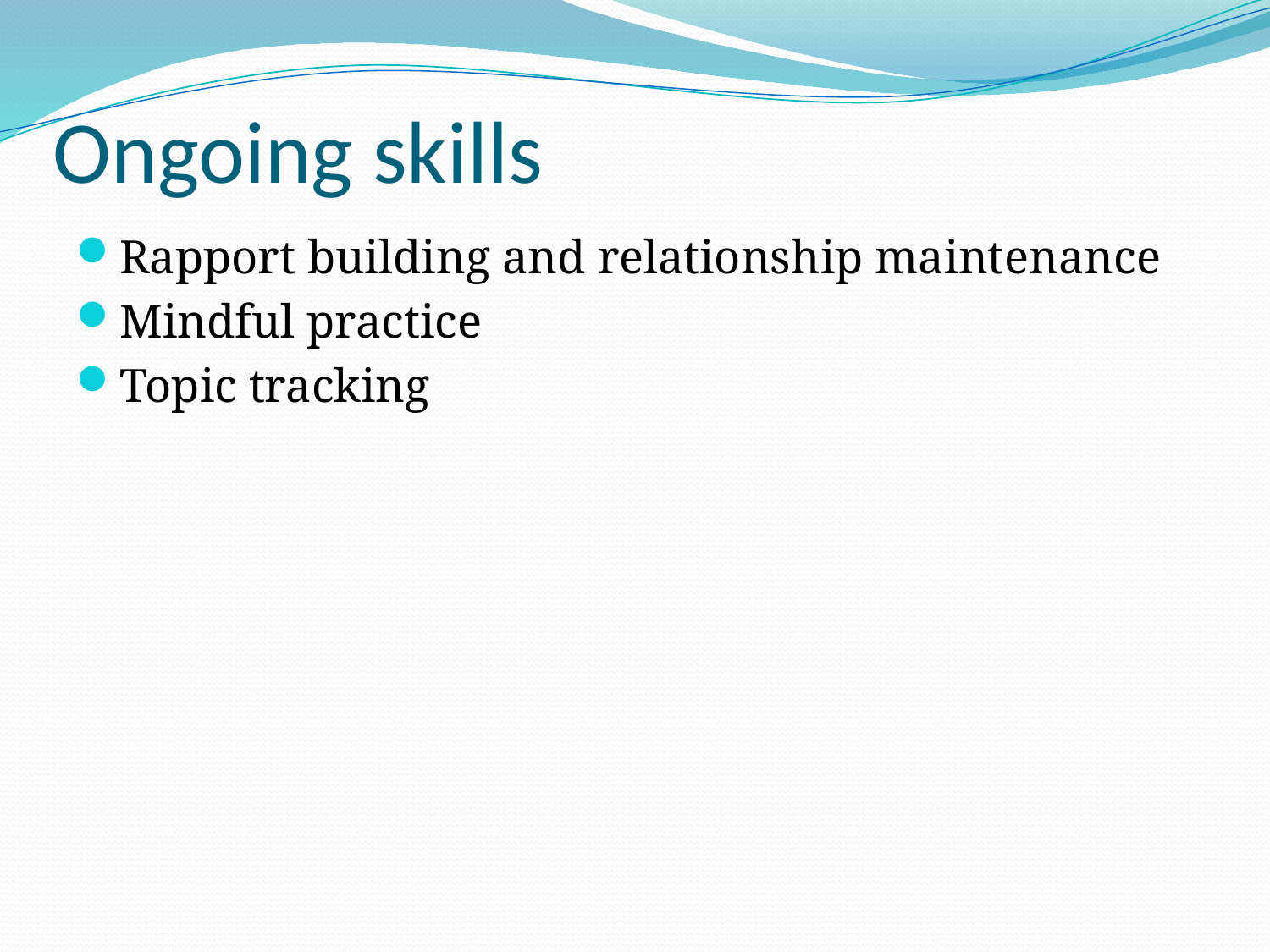

# Ongoing skills
Rapport building and relationship maintenance
Mindful practice
Topic tracking

## Slide 5
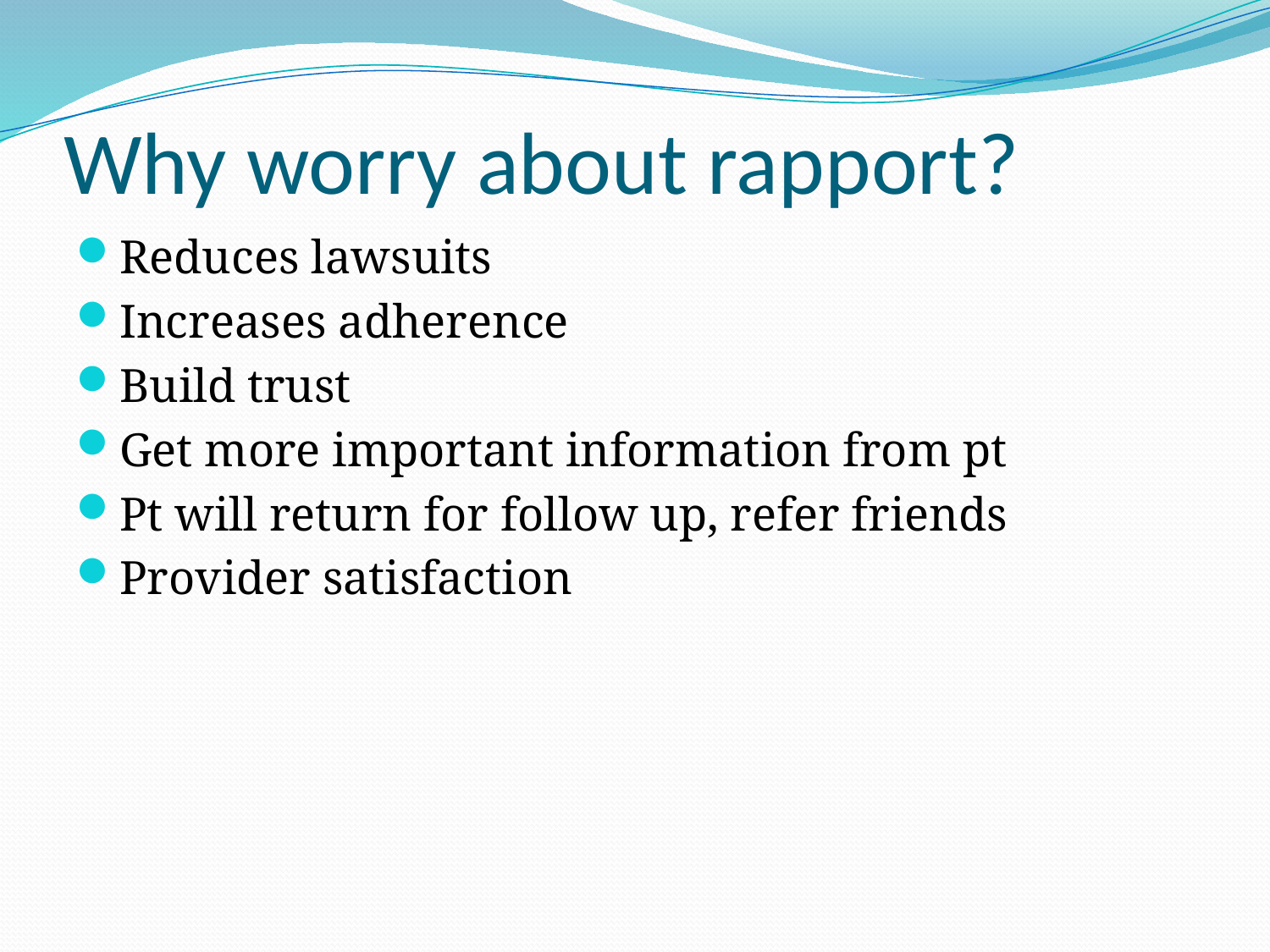

# Why worry about rapport?
Reduces lawsuits
Increases adherence
Build trust
Get more important information from pt
Pt will return for follow up, refer friends
Provider satisfaction

## Slide 6
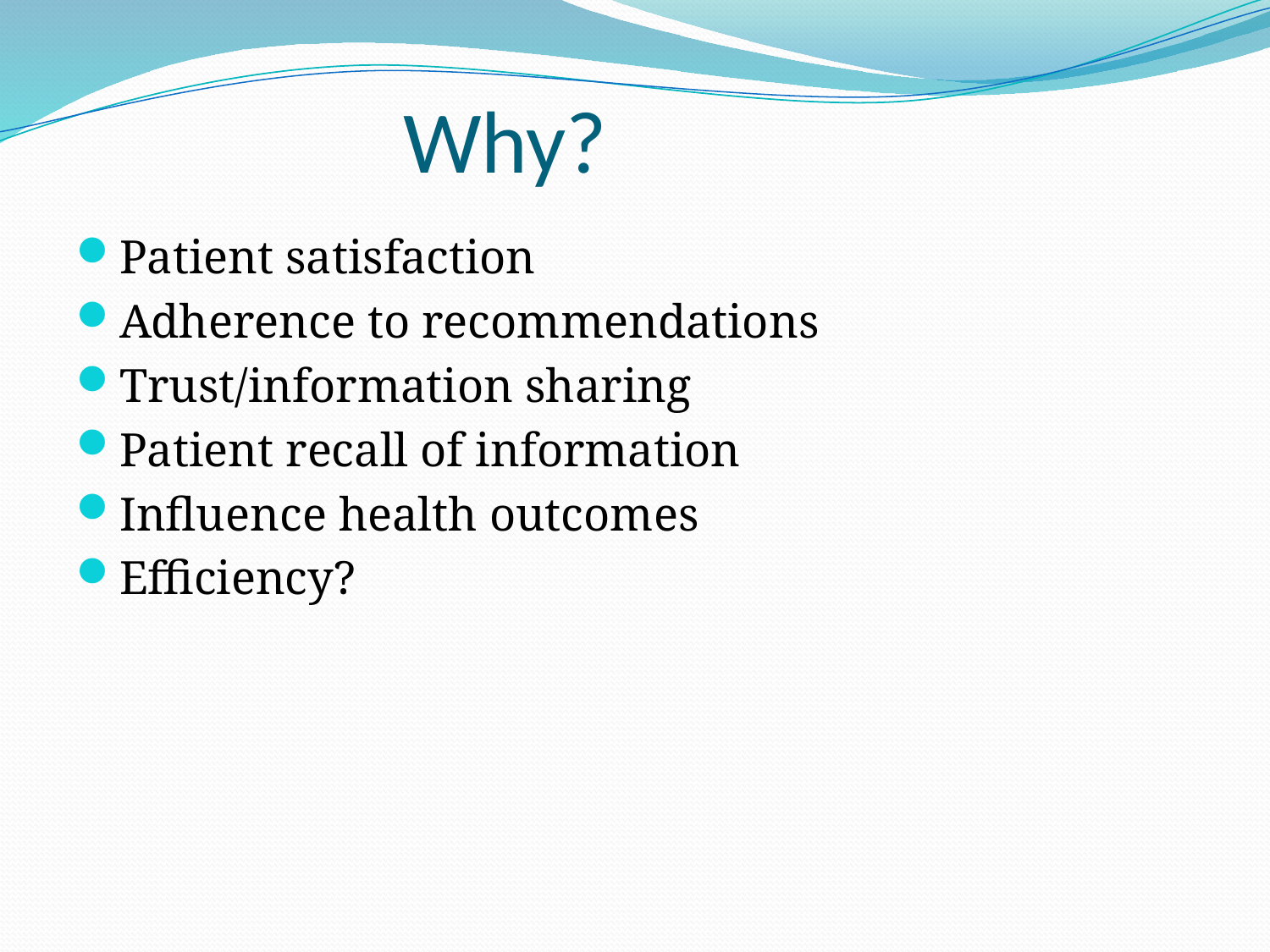

# Why?
Patient satisfaction
Adherence to recommendations
Trust/information sharing
Patient recall of information
Influence health outcomes
Efficiency?

## Slide 7
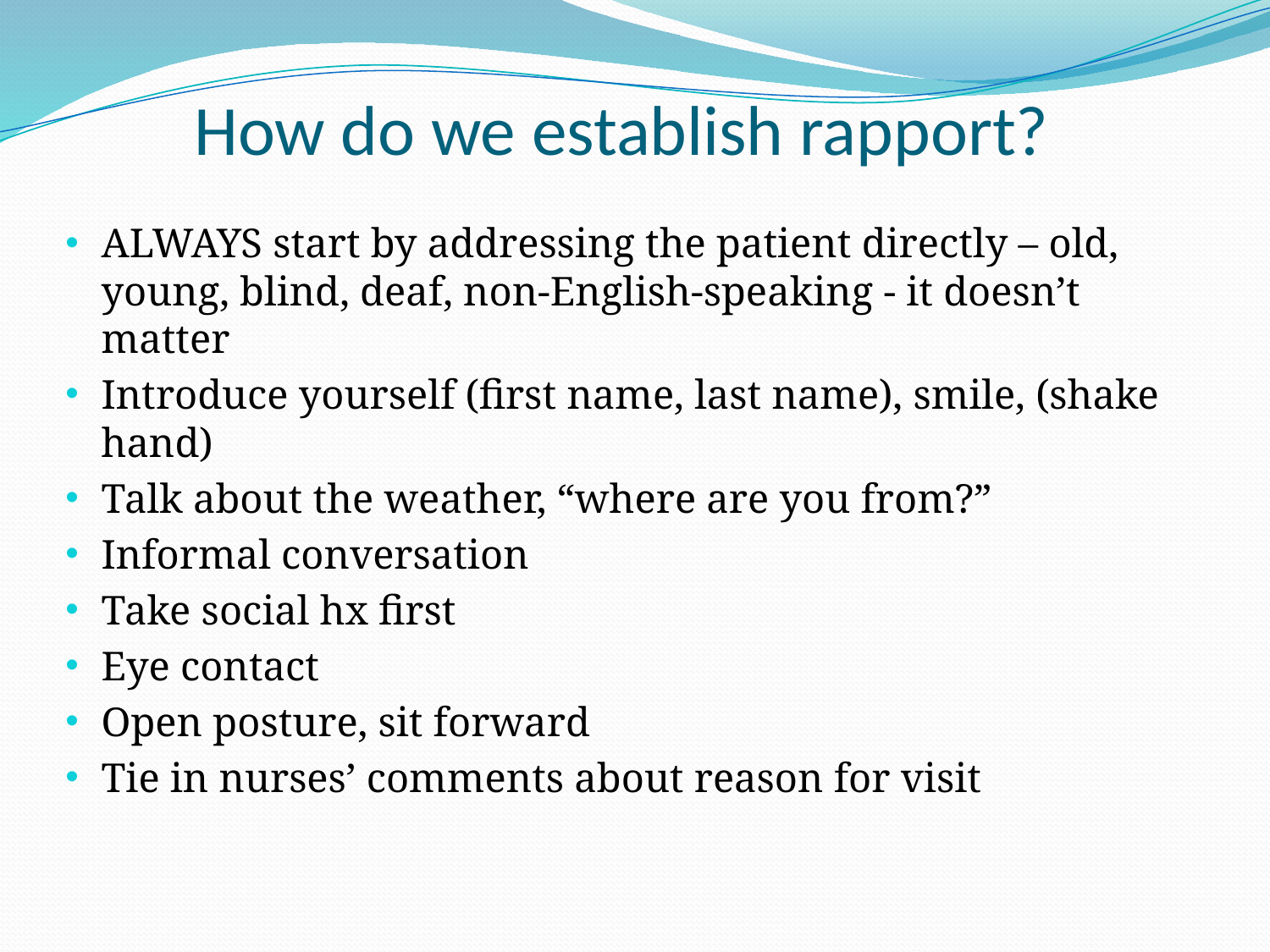

# How do we establish rapport?
ALWAYS start by addressing the patient directly – old, young, blind, deaf, non-English-speaking - it doesn’t matter
Introduce yourself (first name, last name), smile, (shake hand)
Talk about the weather, “where are you from?”
Informal conversation
Take social hx first
Eye contact
Open posture, sit forward
Tie in nurses’ comments about reason for visit

## Slide 8
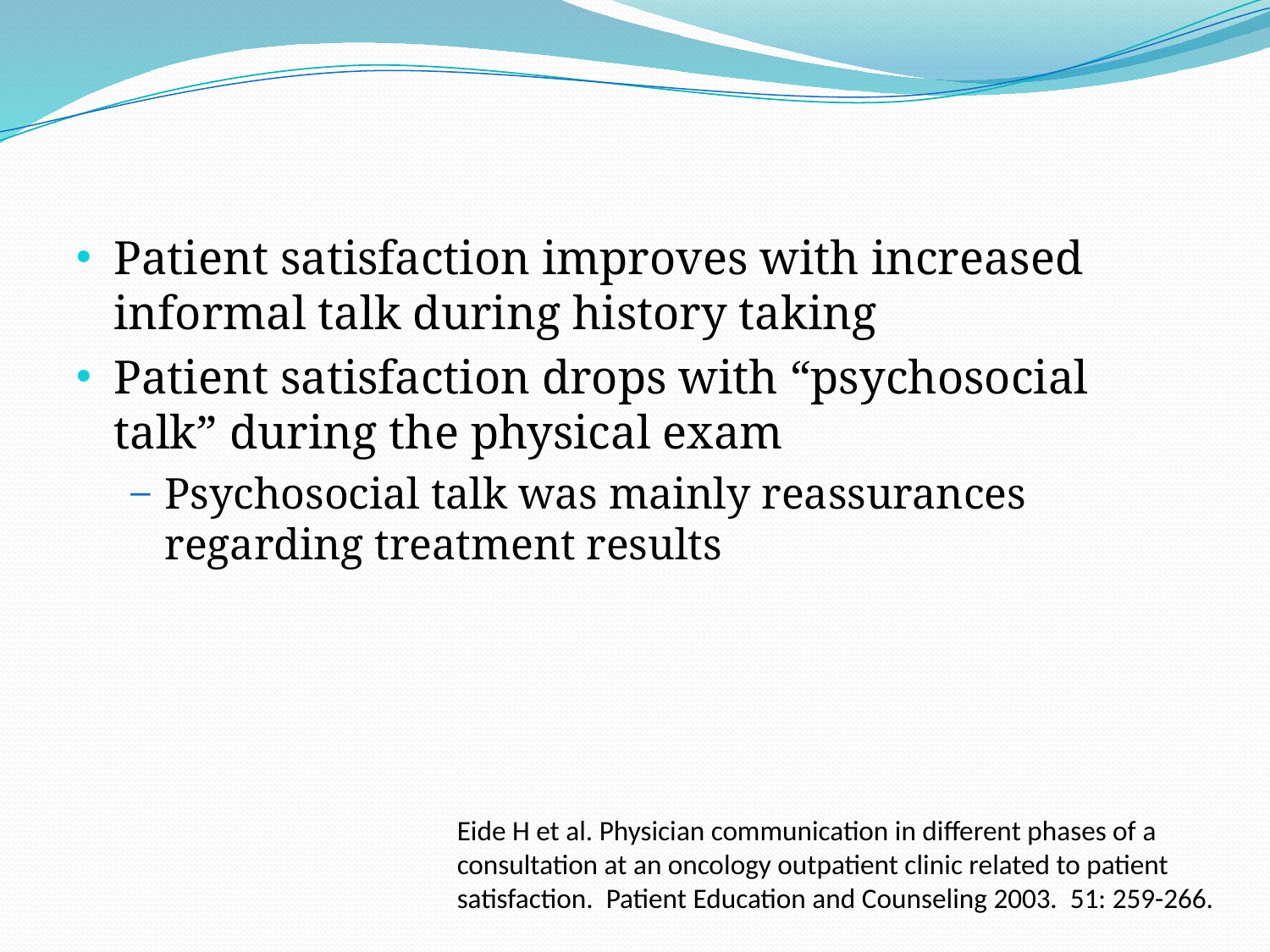

Patient satisfaction improves with increased informal talk during history taking
Patient satisfaction drops with “psychosocial talk” during the physical exam
Psychosocial talk was mainly reassurances regarding treatment results
Eide H et al. Physician communication in different phases of a consultation at an oncology outpatient clinic related to patient satisfaction. Patient Education and Counseling 2003. 51: 259-266.

## Slide 9
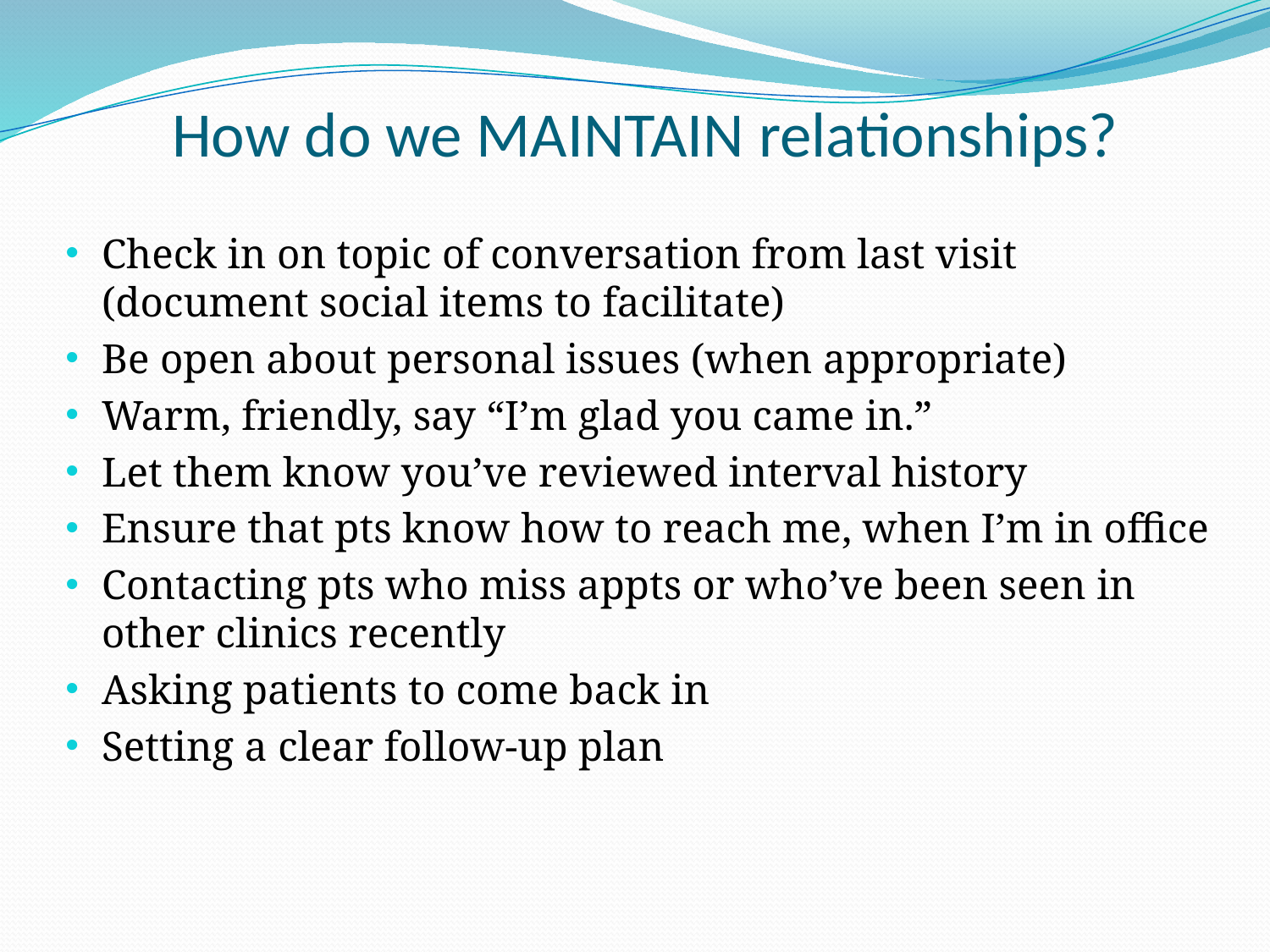

# How do we MAINTAIN relationships?
Check in on topic of conversation from last visit (document social items to facilitate)
Be open about personal issues (when appropriate)
Warm, friendly, say “I’m glad you came in.”
Let them know you’ve reviewed interval history
Ensure that pts know how to reach me, when I’m in office
Contacting pts who miss appts or who’ve been seen in other clinics recently
Asking patients to come back in
Setting a clear follow-up plan

## Slide 10
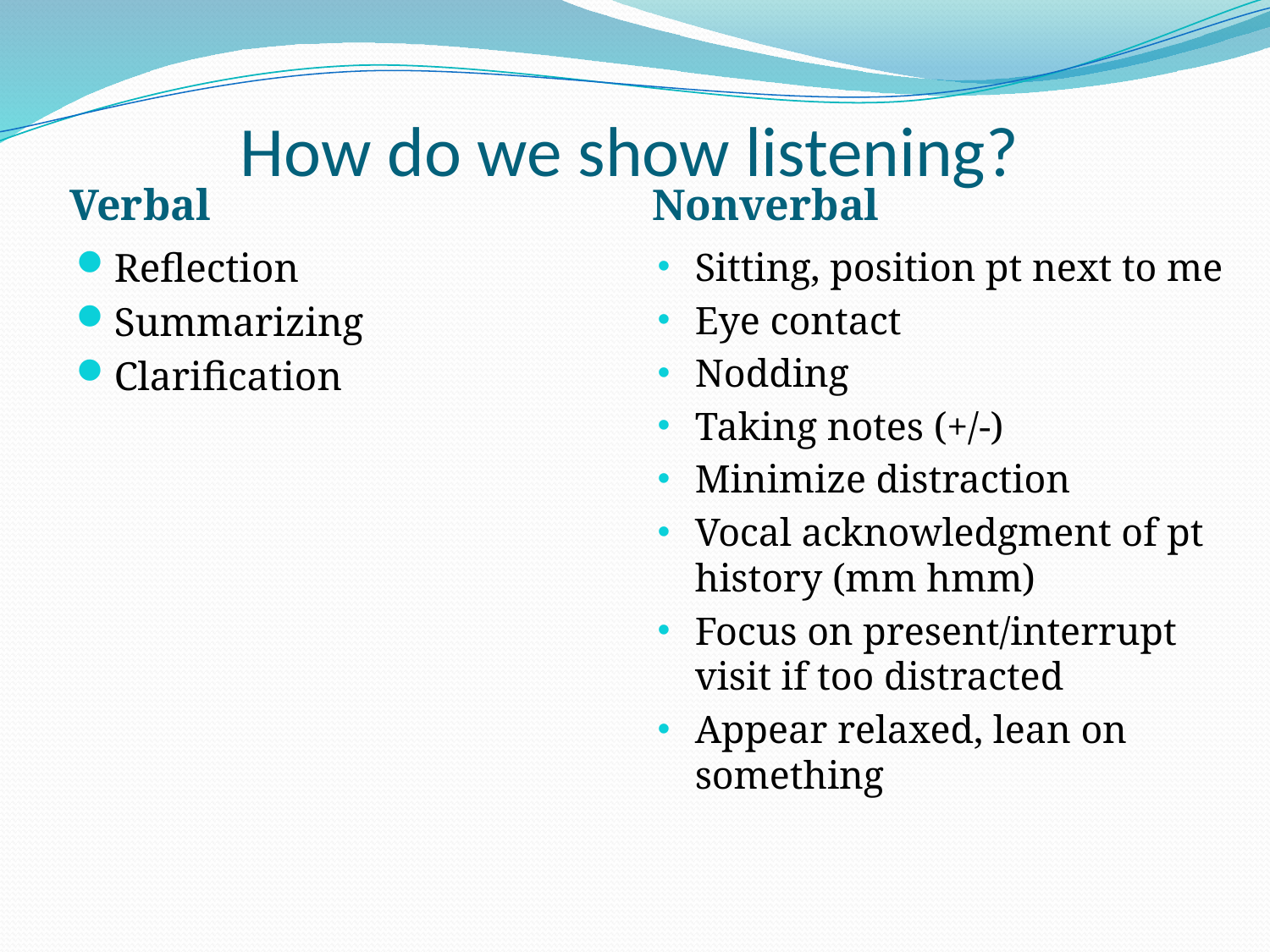

# How do we show listening?
Verbal
Nonverbal
Reflection
Summarizing
Clarification
Sitting, position pt next to me
Eye contact
Nodding
Taking notes (+/-)
Minimize distraction
Vocal acknowledgment of pt history (mm hmm)
Focus on present/interrupt visit if too distracted
Appear relaxed, lean on something

## Slide 11
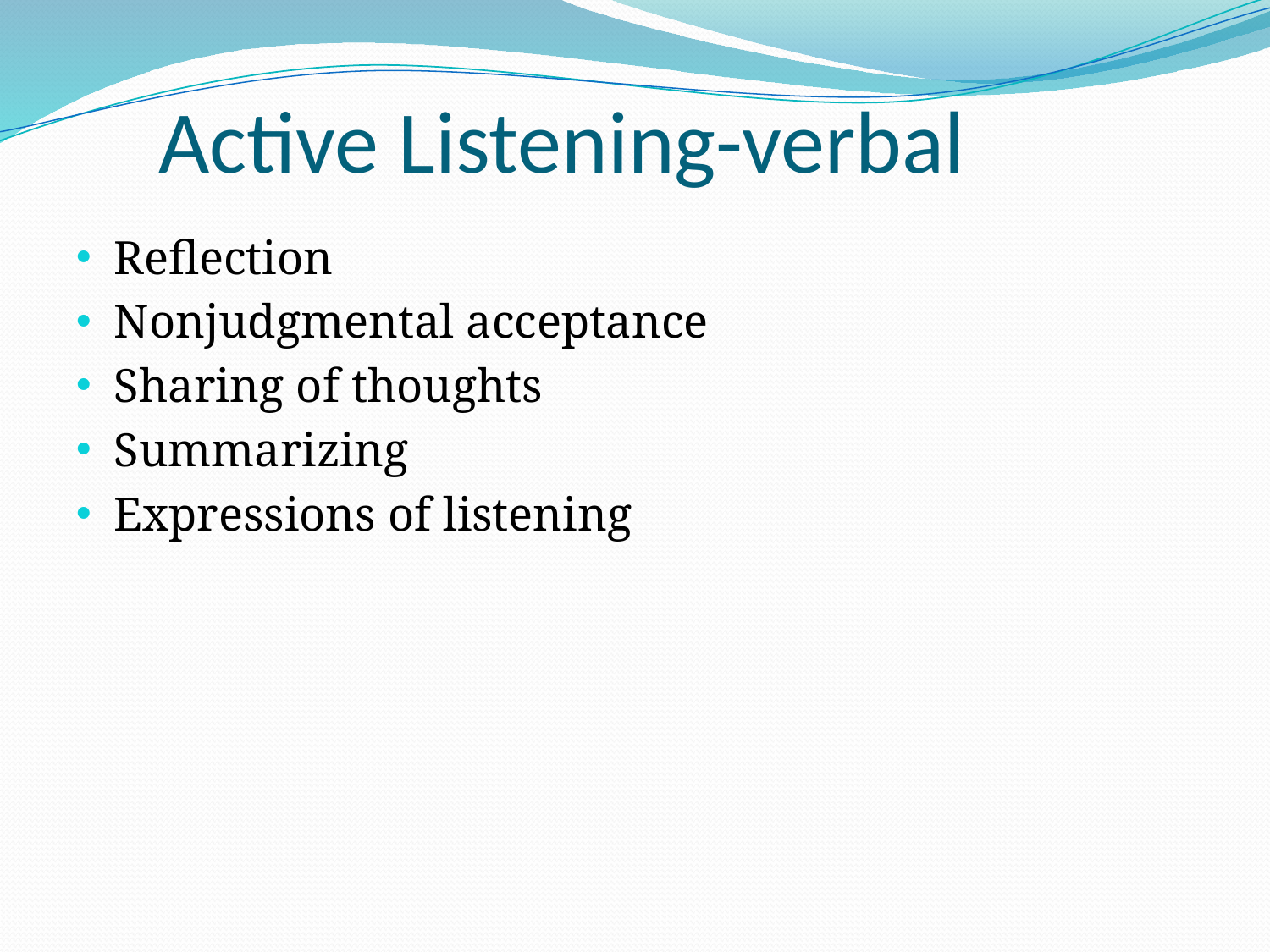

# Active Listening-verbal
Reflection
Nonjudgmental acceptance
Sharing of thoughts
Summarizing
Expressions of listening

## Slide 12
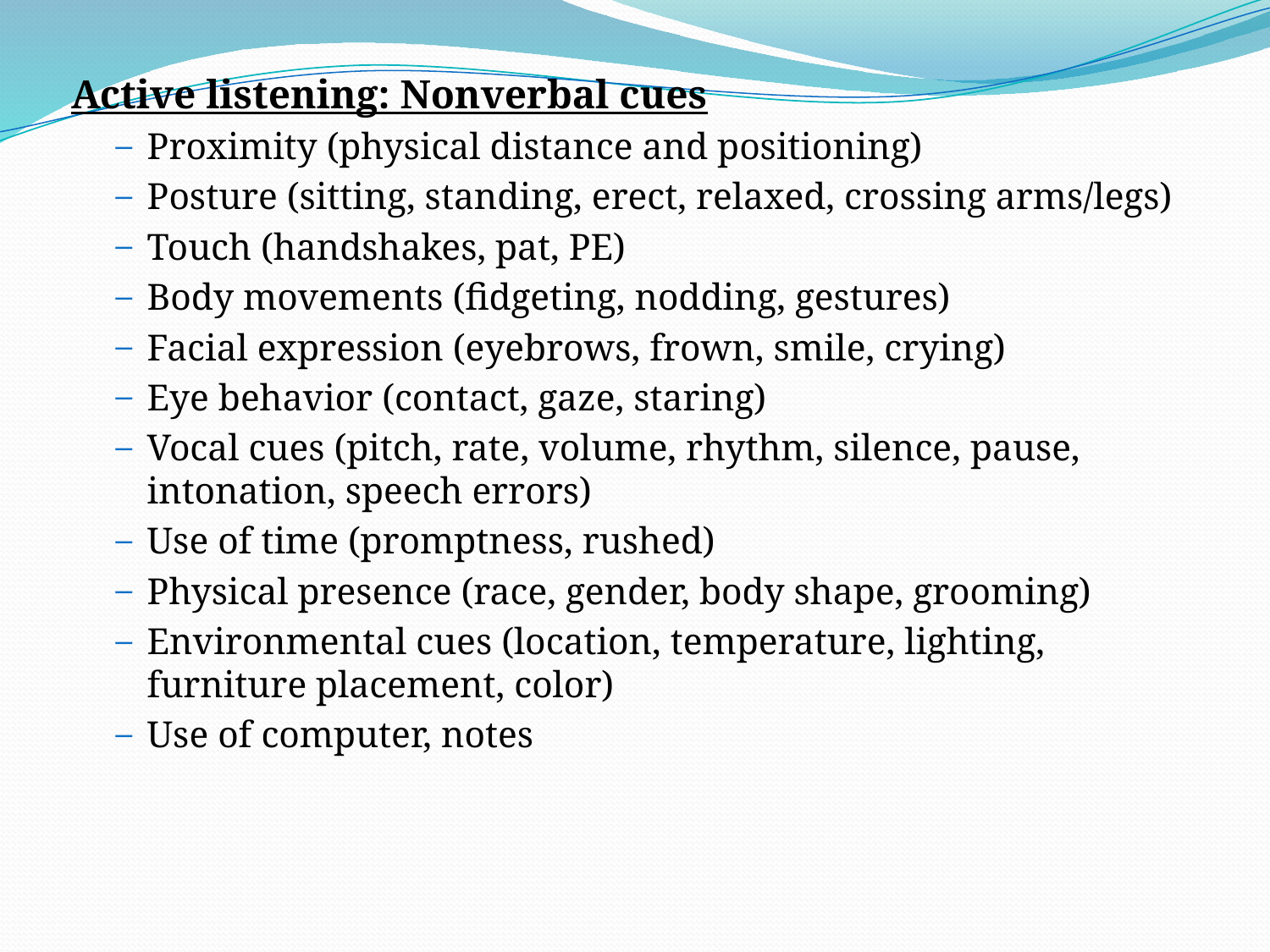

Active listening: Nonverbal cues
Proximity (physical distance and positioning)
Posture (sitting, standing, erect, relaxed, crossing arms/legs)
Touch (handshakes, pat, PE)
Body movements (fidgeting, nodding, gestures)
Facial expression (eyebrows, frown, smile, crying)
Eye behavior (contact, gaze, staring)
Vocal cues (pitch, rate, volume, rhythm, silence, pause, intonation, speech errors)
Use of time (promptness, rushed)
Physical presence (race, gender, body shape, grooming)
Environmental cues (location, temperature, lighting, furniture placement, color)
Use of computer, notes

## Slide 13
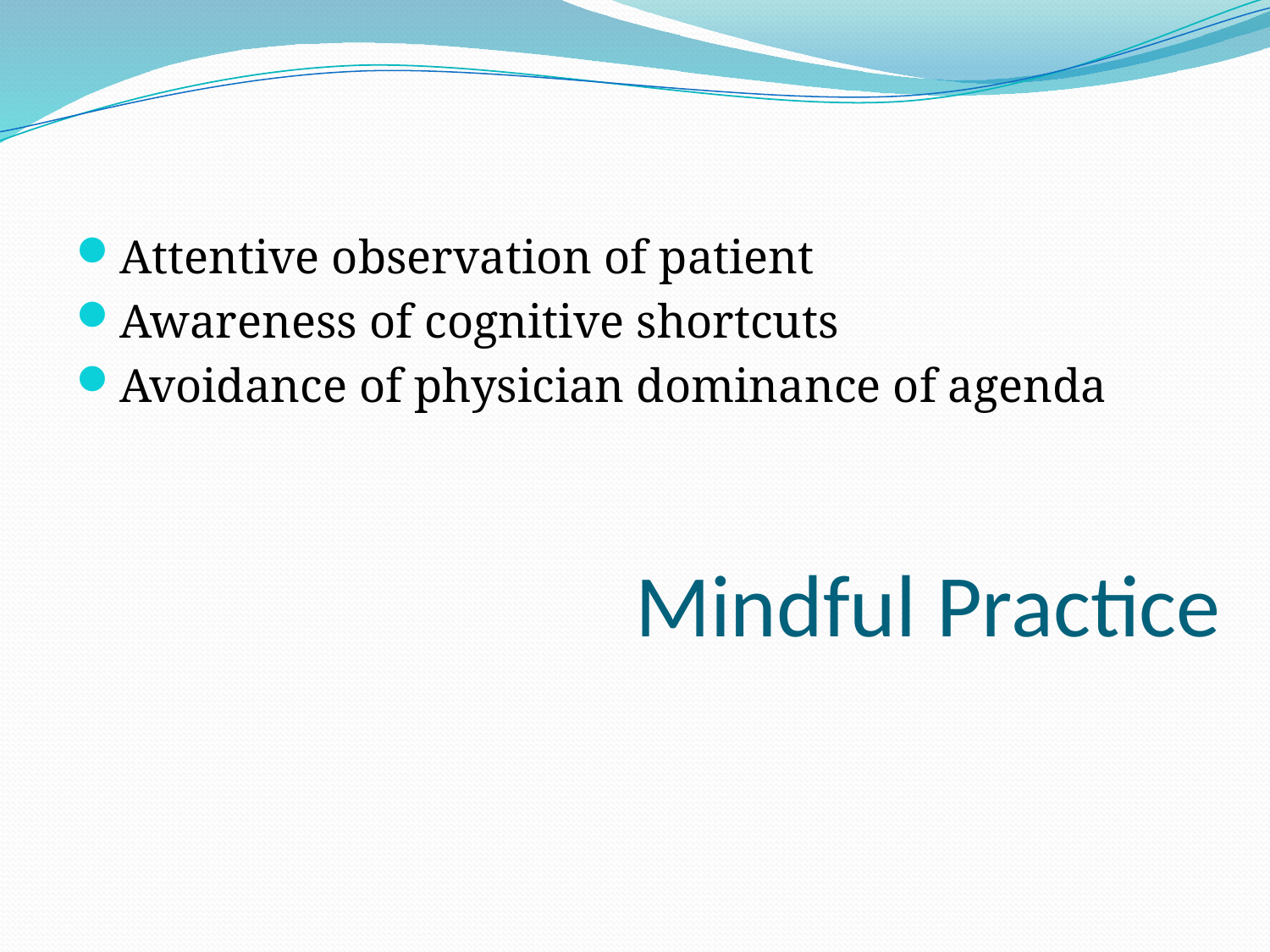

Attentive observation of patient
Awareness of cognitive shortcuts
Avoidance of physician dominance of agenda
# Mindful Practice

## Slide 14
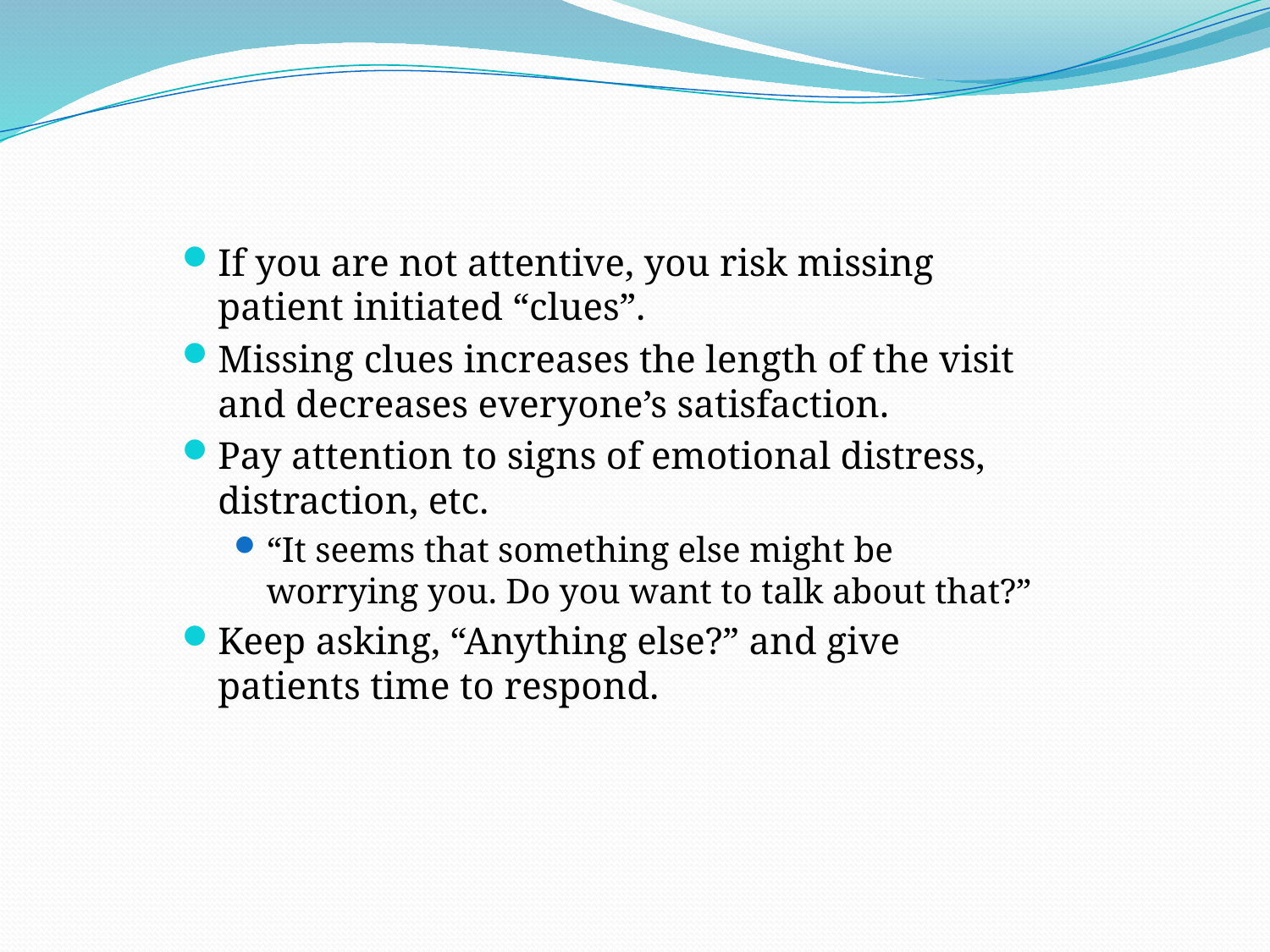

If you are not attentive, you risk missing patient initiated “clues”.
Missing clues increases the length of the visit and decreases everyone’s satisfaction.
Pay attention to signs of emotional distress, distraction, etc.
“It seems that something else might be worrying you. Do you want to talk about that?”
Keep asking, “Anything else?” and give patients time to respond.

## Slide 15
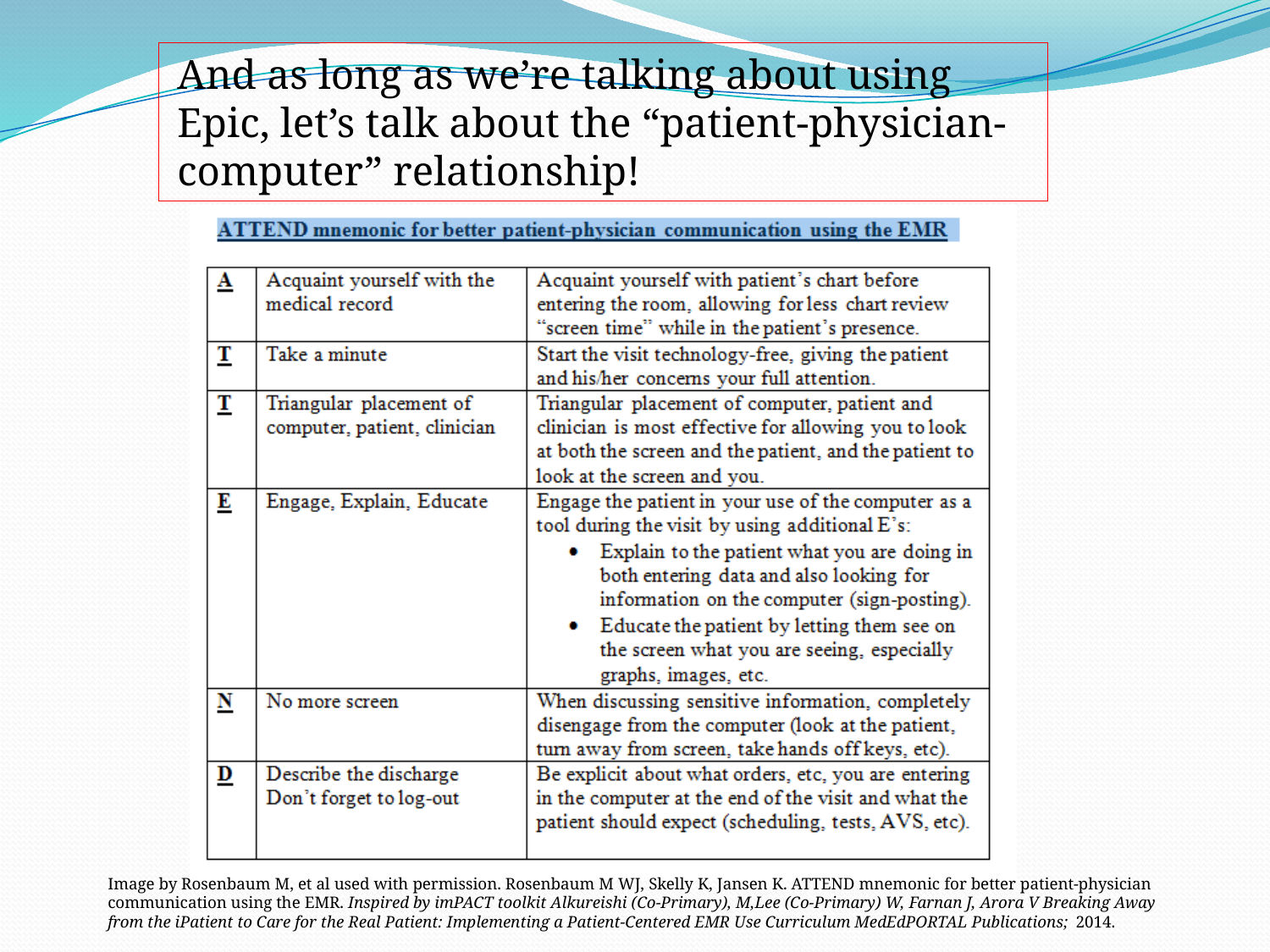

And as long as we’re talking about using Epic, let’s talk about the “patient-physician-computer” relationship!
Image by Rosenbaum M, et al used with permission. Rosenbaum M WJ, Skelly K, Jansen K. ATTEND mnemonic for better patient-physician communication using the EMR. Inspired by imPACT toolkit Alkureishi (Co-Primary), M,Lee (Co-Primary) W, Farnan J, Arora V Breaking Away from the iPatient to Care for the Real Patient: Implementing a Patient-Centered EMR Use Curriculum MedEdPORTAL Publications; 2014.

## Slide 16
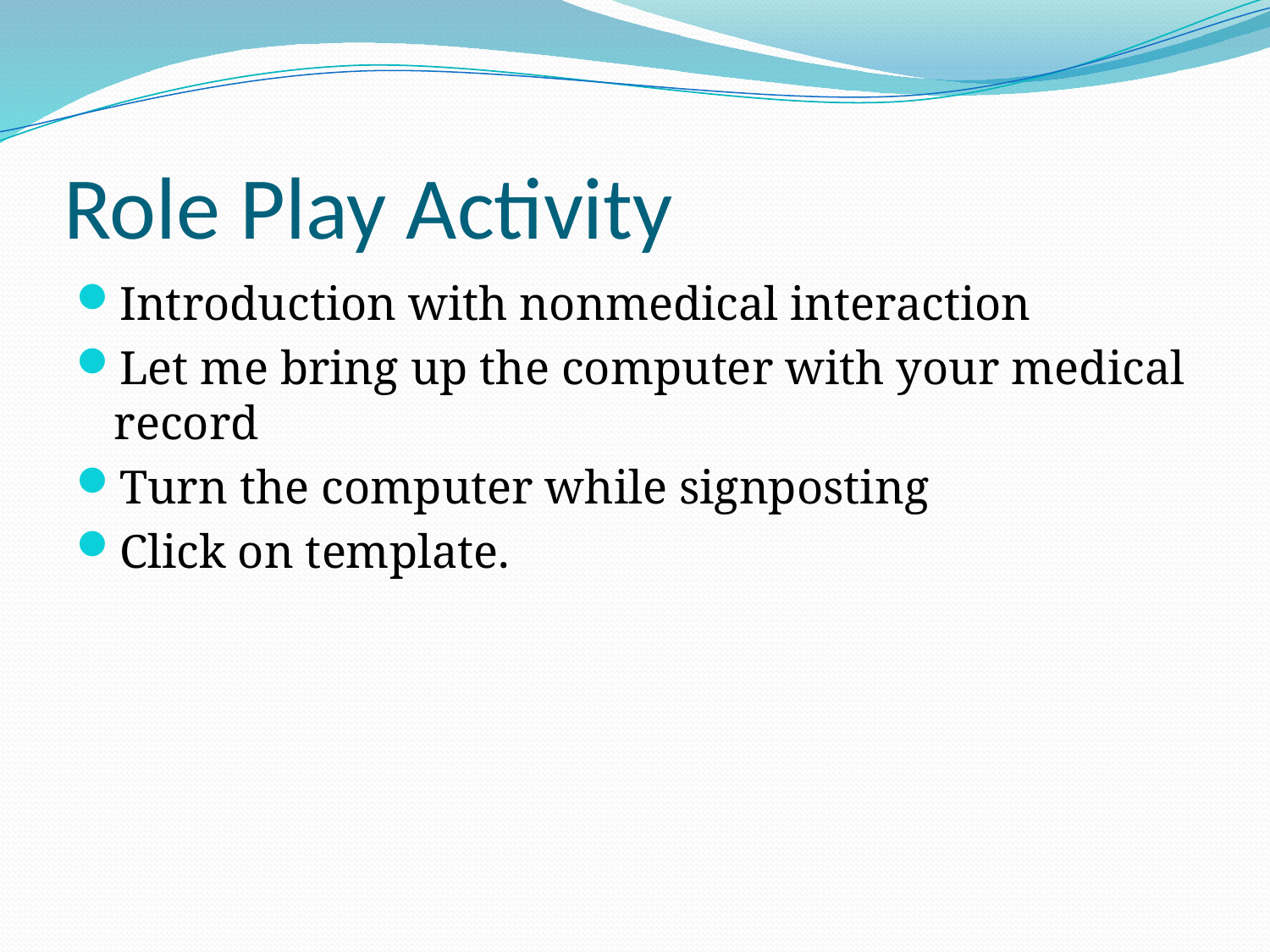

# Role Play Activity
Introduction with nonmedical interaction
Let me bring up the computer with your medical record
Turn the computer while signposting
Click on template.

## Slide 17
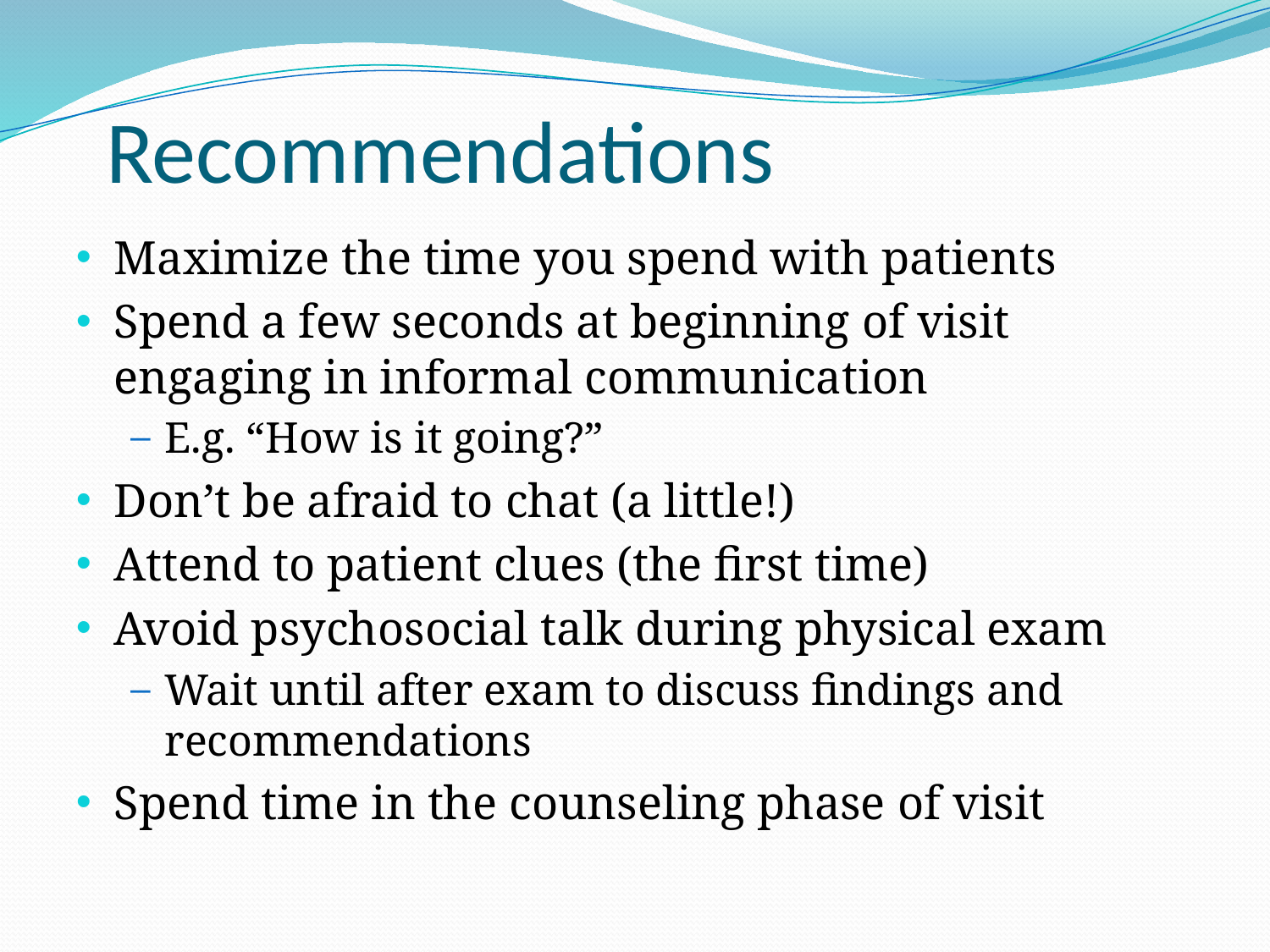

# Recommendations
Maximize the time you spend with patients
Spend a few seconds at beginning of visit engaging in informal communication
E.g. “How is it going?”
Don’t be afraid to chat (a little!)
Attend to patient clues (the first time)
Avoid psychosocial talk during physical exam
Wait until after exam to discuss findings and recommendations
Spend time in the counseling phase of visit

## Slide 18
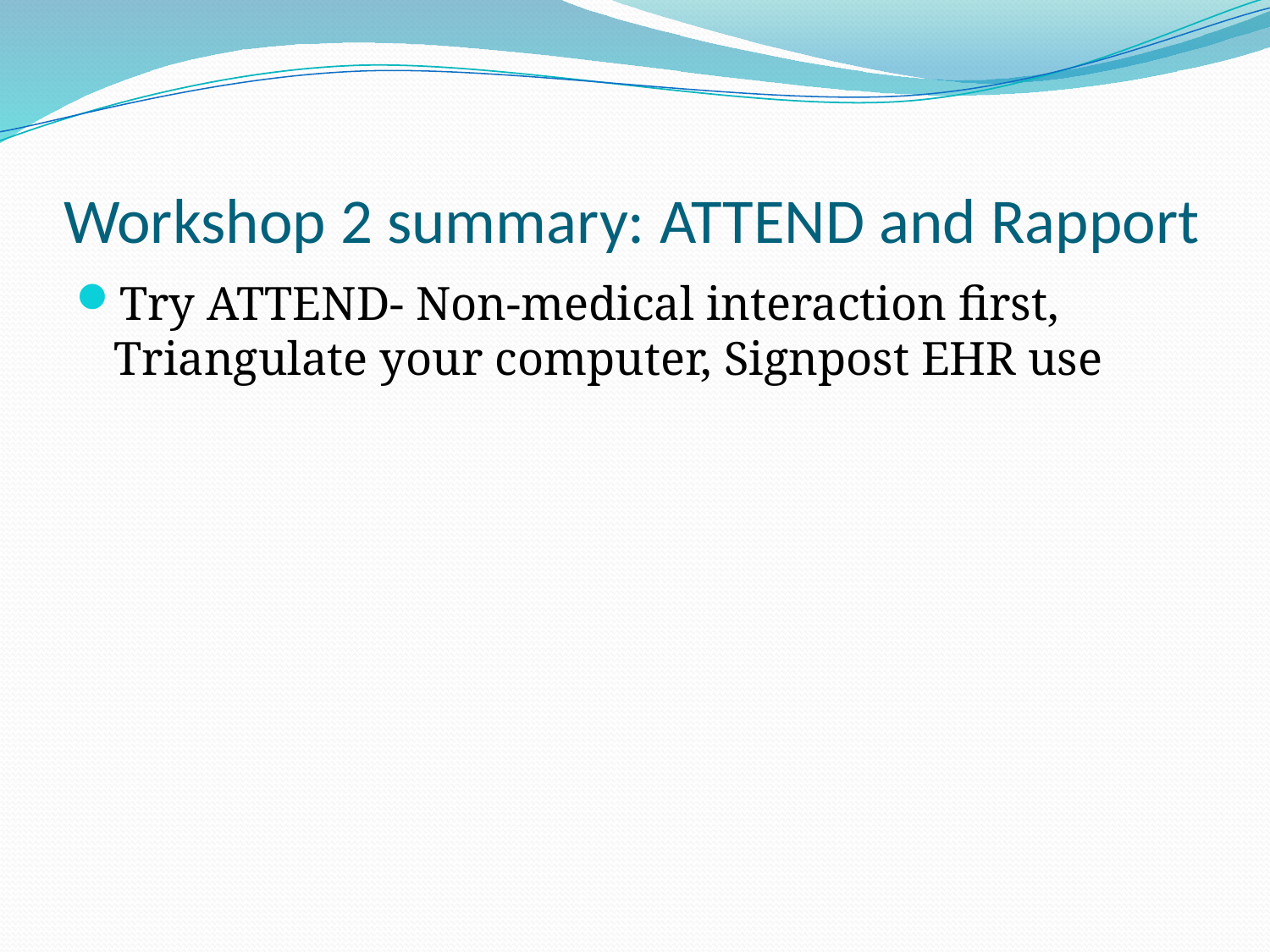

# Workshop 2 summary: ATTEND and Rapport
Try ATTEND- Non-medical interaction first, Triangulate your computer, Signpost EHR use

## Slide 19
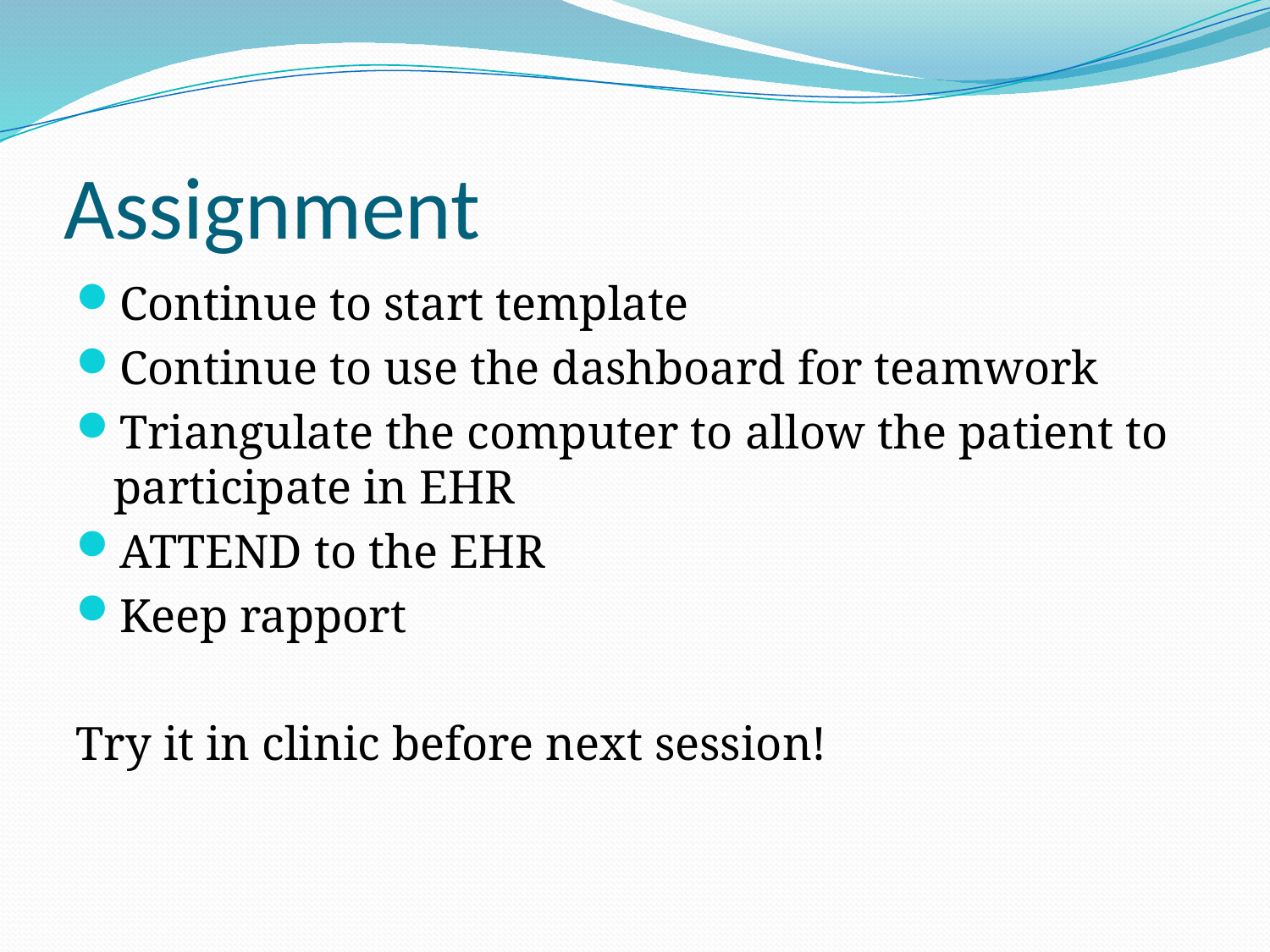

# Assignment
Continue to start template
Continue to use the dashboard for teamwork
Triangulate the computer to allow the patient to participate in EHR
ATTEND to the EHR
Keep rapport
Try it in clinic before next session!

## Slide 20
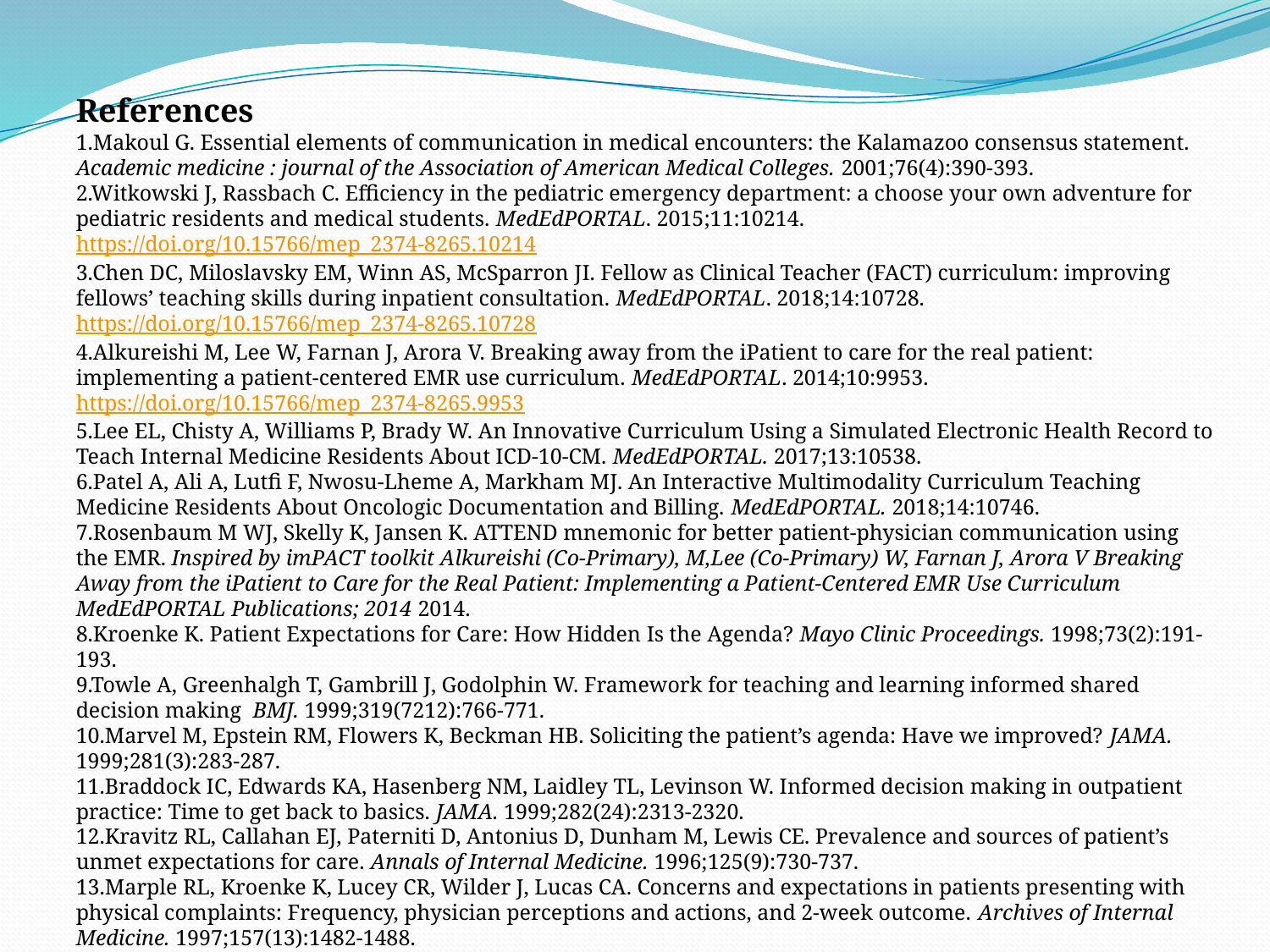

References
1.Makoul G. Essential elements of communication in medical encounters: the Kalamazoo consensus statement. Academic medicine : journal of the Association of American Medical Colleges. 2001;76(4):390-393.
2.Witkowski J, Rassbach C. Efficiency in the pediatric emergency department: a choose your own adventure for pediatric residents and medical students. MedEdPORTAL. 2015;11:10214. https://doi.org/10.15766/mep_2374-8265.10214
3.Chen DC, Miloslavsky EM, Winn AS, McSparron JI. Fellow as Clinical Teacher (FACT) curriculum: improving fellows’ teaching skills during inpatient consultation. MedEdPORTAL. 2018;14:10728. https://doi.org/10.15766/mep_2374-8265.10728
4.Alkureishi M, Lee W, Farnan J, Arora V. Breaking away from the iPatient to care for the real patient: implementing a patient-centered EMR use curriculum. MedEdPORTAL. 2014;10:9953. https://doi.org/10.15766/mep_2374-8265.9953
5.Lee EL, Chisty A, Williams P, Brady W. An Innovative Curriculum Using a Simulated Electronic Health Record to Teach Internal Medicine Residents About ICD-10-CM. MedEdPORTAL. 2017;13:10538.
6.Patel A, Ali A, Lutfi F, Nwosu-Lheme A, Markham MJ. An Interactive Multimodality Curriculum Teaching Medicine Residents About Oncologic Documentation and Billing. MedEdPORTAL. 2018;14:10746.
7.Rosenbaum M WJ, Skelly K, Jansen K. ATTEND mnemonic for better patient-physician communication using the EMR. Inspired by imPACT toolkit Alkureishi (Co-Primary), M,Lee (Co-Primary) W, Farnan J, Arora V Breaking Away from the iPatient to Care for the Real Patient: Implementing a Patient-Centered EMR Use Curriculum MedEdPORTAL Publications; 2014 2014.
8.Kroenke K. Patient Expectations for Care: How Hidden Is the Agenda? Mayo Clinic Proceedings. 1998;73(2):191-193.
9.Towle A, Greenhalgh T, Gambrill J, Godolphin W. Framework for teaching and learning informed shared decision making BMJ. 1999;319(7212):766-771.
10.Marvel M, Epstein RM, Flowers K, Beckman HB. Soliciting the patient’s agenda: Have we improved? JAMA. 1999;281(3):283-287.
11.Braddock IC, Edwards KA, Hasenberg NM, Laidley TL, Levinson W. Informed decision making in outpatient practice: Time to get back to basics. JAMA. 1999;282(24):2313-2320.
12.Kravitz RL, Callahan EJ, Paterniti D, Antonius D, Dunham M, Lewis CE. Prevalence and sources of patient’s unmet expectations for care. Annals of Internal Medicine. 1996;125(9):730-737.
13.Marple RL, Kroenke K, Lucey CR, Wilder J, Lucas CA. Concerns and expectations in patients presenting with physical complaints: Frequency, physician perceptions and actions, and 2-week outcome. Archives of Internal Medicine. 1997;157(13):1482-1488.
14.Mauksch L, Hillenburg L, Robins L. The Establishing Focus protocol: Training for collaborative agenda setting and time management in the medical interview. Vol 192001.
15.Østbye T, Yarnall KSH, Krause KM, Pollak KI, Gradison M, Michener JL. Is There Time for Management of Patients With Chronic Diseases in Primary Care? The Annals of Family Medicine. 2005;3(3):209-214.

## Slide 21
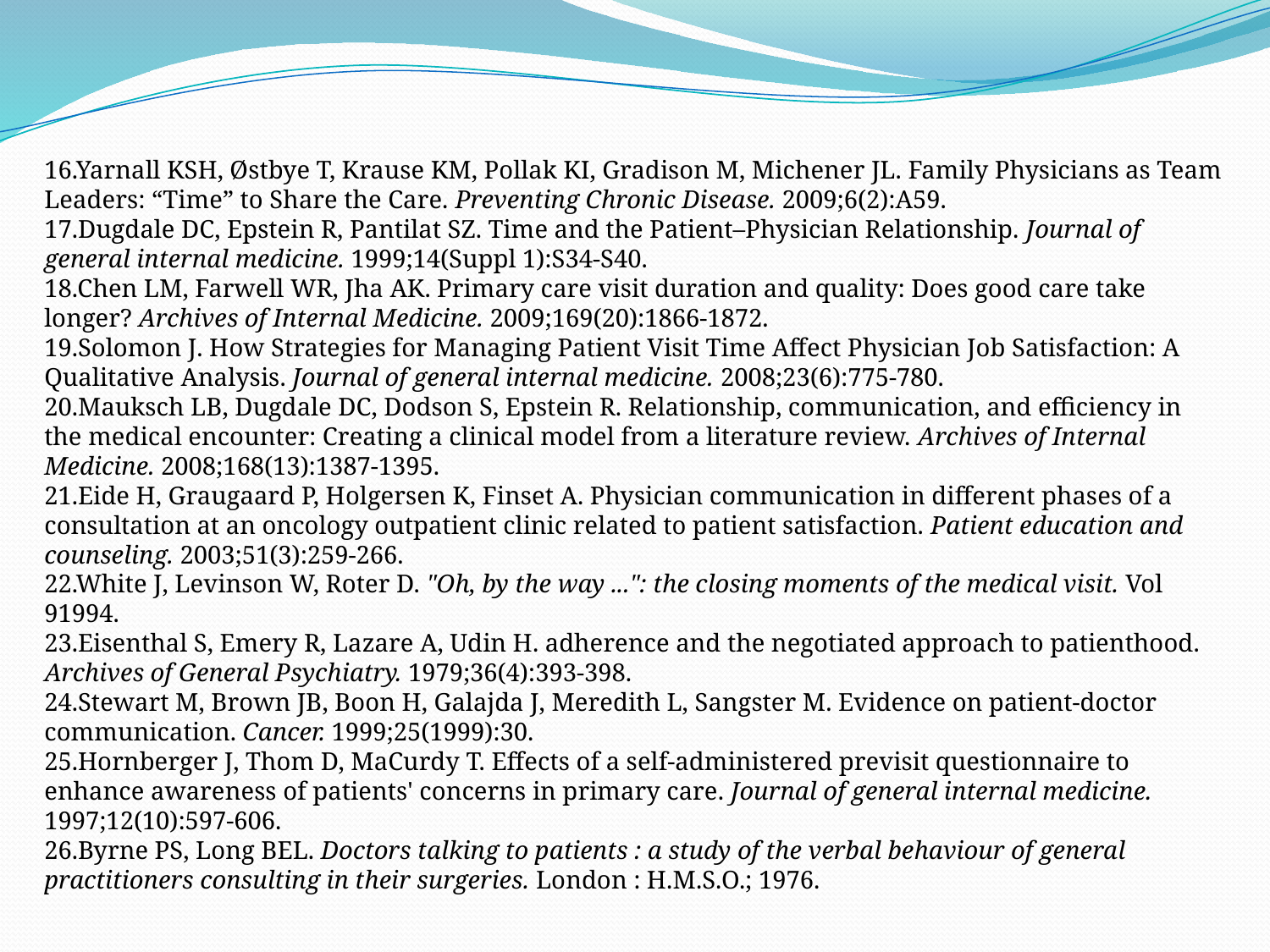

16.Yarnall KSH, Østbye T, Krause KM, Pollak KI, Gradison M, Michener JL. Family Physicians as Team Leaders: “Time” to Share the Care. Preventing Chronic Disease. 2009;6(2):A59.
17.Dugdale DC, Epstein R, Pantilat SZ. Time and the Patient–Physician Relationship. Journal of general internal medicine. 1999;14(Suppl 1):S34-S40.
18.Chen LM, Farwell WR, Jha AK. Primary care visit duration and quality: Does good care take longer? Archives of Internal Medicine. 2009;169(20):1866-1872.
19.Solomon J. How Strategies for Managing Patient Visit Time Affect Physician Job Satisfaction: A Qualitative Analysis. Journal of general internal medicine. 2008;23(6):775-780.
20.Mauksch LB, Dugdale DC, Dodson S, Epstein R. Relationship, communication, and efficiency in the medical encounter: Creating a clinical model from a literature review. Archives of Internal Medicine. 2008;168(13):1387-1395.
21.Eide H, Graugaard P, Holgersen K, Finset A. Physician communication in different phases of a consultation at an oncology outpatient clinic related to patient satisfaction. Patient education and counseling. 2003;51(3):259-266.
22.White J, Levinson W, Roter D. "Oh, by the way ...": the closing moments of the medical visit. Vol 91994.
23.Eisenthal S, Emery R, Lazare A, Udin H. adherence and the negotiated approach to patienthood. Archives of General Psychiatry. 1979;36(4):393-398.
24.Stewart M, Brown JB, Boon H, Galajda J, Meredith L, Sangster M. Evidence on patient-doctor communication. Cancer. 1999;25(1999):30.
25.Hornberger J, Thom D, MaCurdy T. Effects of a self-administered previsit questionnaire to enhance awareness of patients' concerns in primary care. Journal of general internal medicine. 1997;12(10):597-606.
26.Byrne PS, Long BEL. Doctors talking to patients : a study of the verbal behaviour of general practitioners consulting in their surgeries. London : H.M.S.O.; 1976.
