# Supplementary material for: A Curriculum for Teaching Clinical Efficiency Focusing on Specific Communication Skills While Maximizing the Electronic Health Record
Source: MedEdPORTAL. 2020 Oct 29;16:10989. doi: 10.15766/mep_2374-8265.10989 (PMC7597939; doi:10.15766/mep_2374-8265.10989)
Supplement: Supplementary file 1 — Efficiency Preworkshop Needs Assessment Survey.docxWorkshop 1 - Setting up the Template and Working in EHR.pptxSample Clinic Note and AVS Template.docxWorkshop 2 - Preclinic Preparation and Rapport Building.pptxEfficiency ATTEND Practice Card.docxWorkshop 3 - Agenda Setting and Relationship Maintenance.pptxEfficiency Agenda Setting Practice.docxWorkshop 4 - Visit Closure.pptxEfficiency Closure Card and Cases.docxEfficiency Postworkshop Evaluation.docx [file mep_2374-8265.10989-s001.zip › E. Efficiency ATTEND Practice Card.docx]

**Appendix E: Attend Practice Card**

**ATTEND mnemonic for better patient-physician communication using the EMR**

| **A** | Acquaint yourself with the medical record | Acquaint yourself with patient’s chart before entering the room, allowing for less chart review “screen time” while in the patient’s presence. |
| --- | --- | --- |
| **T** | Take a minute | Start the visit technology-free, giving the patient and his/her concerns your full attention. |
| **T** | Triangular placement of computer, patient, clinician | Triangular placement of computer, patient and clinician is most effective for allowing you to look at both the screen and the patient, and the patient to look at the screen and you. |
| **E** | Engage, Explain, Educate | Engage the patient in your use of the computer as a tool during the visit by using additional E’s:   - Explain to the patient what you are doing in both entering data and also looking for information on the computer (sign-posting). - Educate the patient by letting them see on the screen what you are seeing, especially graphs, images, etc. |
| **N** | No more screen | When discussing sensitive information, completely disengage from the computer (look at the patient, turn away from screen, take hands off keys, etc). |
| **D** | Describe the discharge  Don’t forget to log-out | Be explicit about what orders, etc, you are entering in the computer at the end of the visit and what the patient should expect (scheduling, tests, AVS, etc). |

^1^

1. Rosenbaum M WJ, Skelly K, Jansen K. ATTEND mnemonic for better patient-physician communication using the EMR. *Inspired by imPACT toolkit Alkureishi (Co-Primary), M,Lee (Co-Primary) W, Farnan J, Arora V Breaking Away from the iPatient to Care for the Real Patient: Implementing a Patient-Centered EMR Use Curriculum MedEdPORTAL Publications; 2014* 2014.
