# Supplementary material for: A Curriculum for Teaching Clinical Efficiency Focusing on Specific Communication Skills While Maximizing the Electronic Health Record
Source: MedEdPORTAL. 2020 Oct 29;16:10989. doi: 10.15766/mep_2374-8265.10989 (PMC7597939; doi:10.15766/mep_2374-8265.10989)
Supplement: Supplementary file 1 — Efficiency Preworkshop Needs Assessment Survey.docxWorkshop 1 - Setting up the Template and Working in EHR.pptxSample Clinic Note and AVS Template.docxWorkshop 2 - Preclinic Preparation and Rapport Building.pptxEfficiency ATTEND Practice Card.docxWorkshop 3 - Agenda Setting and Relationship Maintenance.pptxEfficiency Agenda Setting Practice.docxWorkshop 4 - Visit Closure.pptxEfficiency Closure Card and Cases.docxEfficiency Postworkshop Evaluation.docx [file mep_2374-8265.10989-s001.zip › F. Workshop 3 - Agenda Setting and Relationship Maintenance.pptx]

## Slide 1
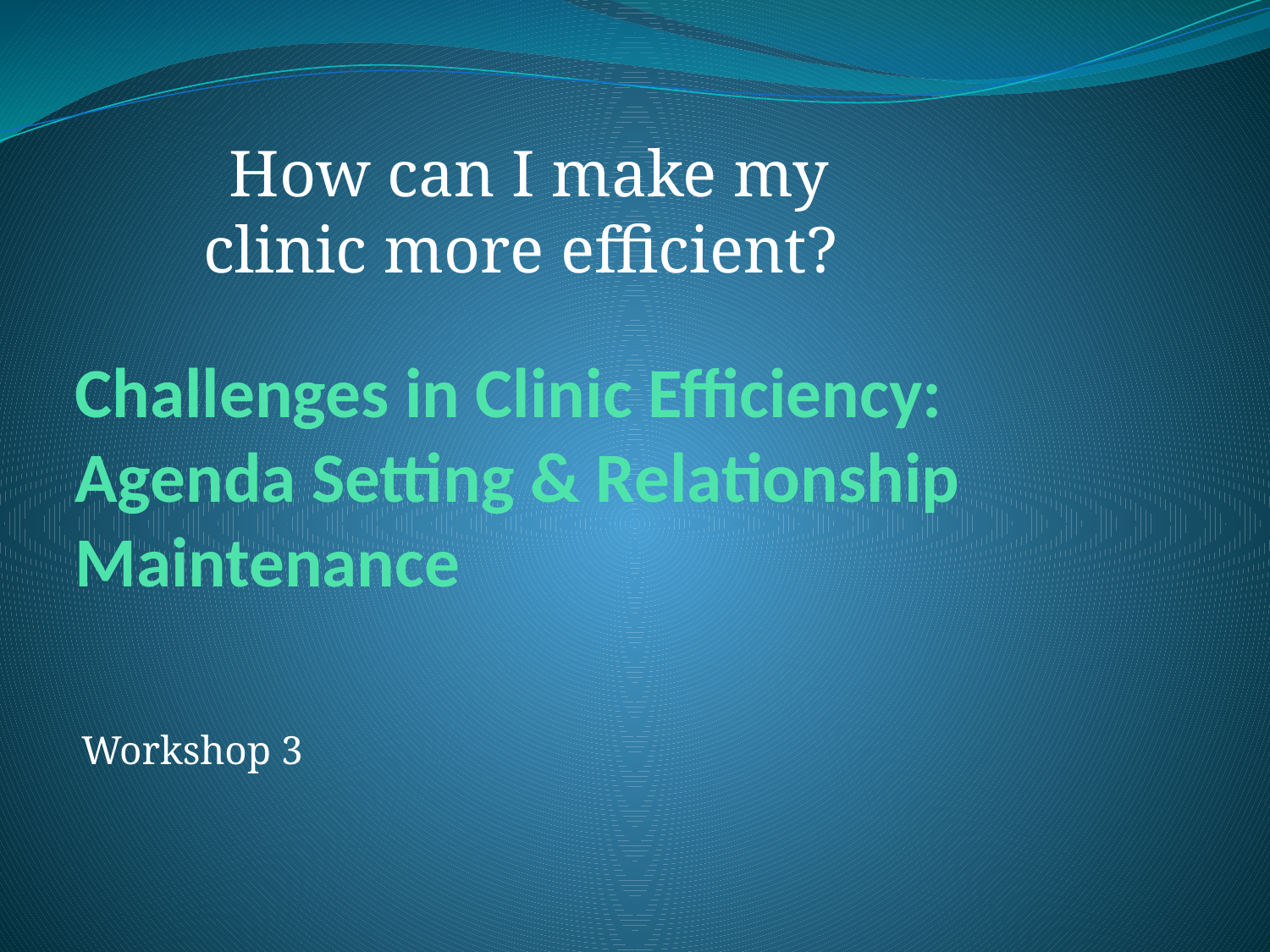

How can I make my clinic more efficient?
# Challenges in Clinic Efficiency: Agenda Setting & Relationship Maintenance
Workshop 3

## Slide 2
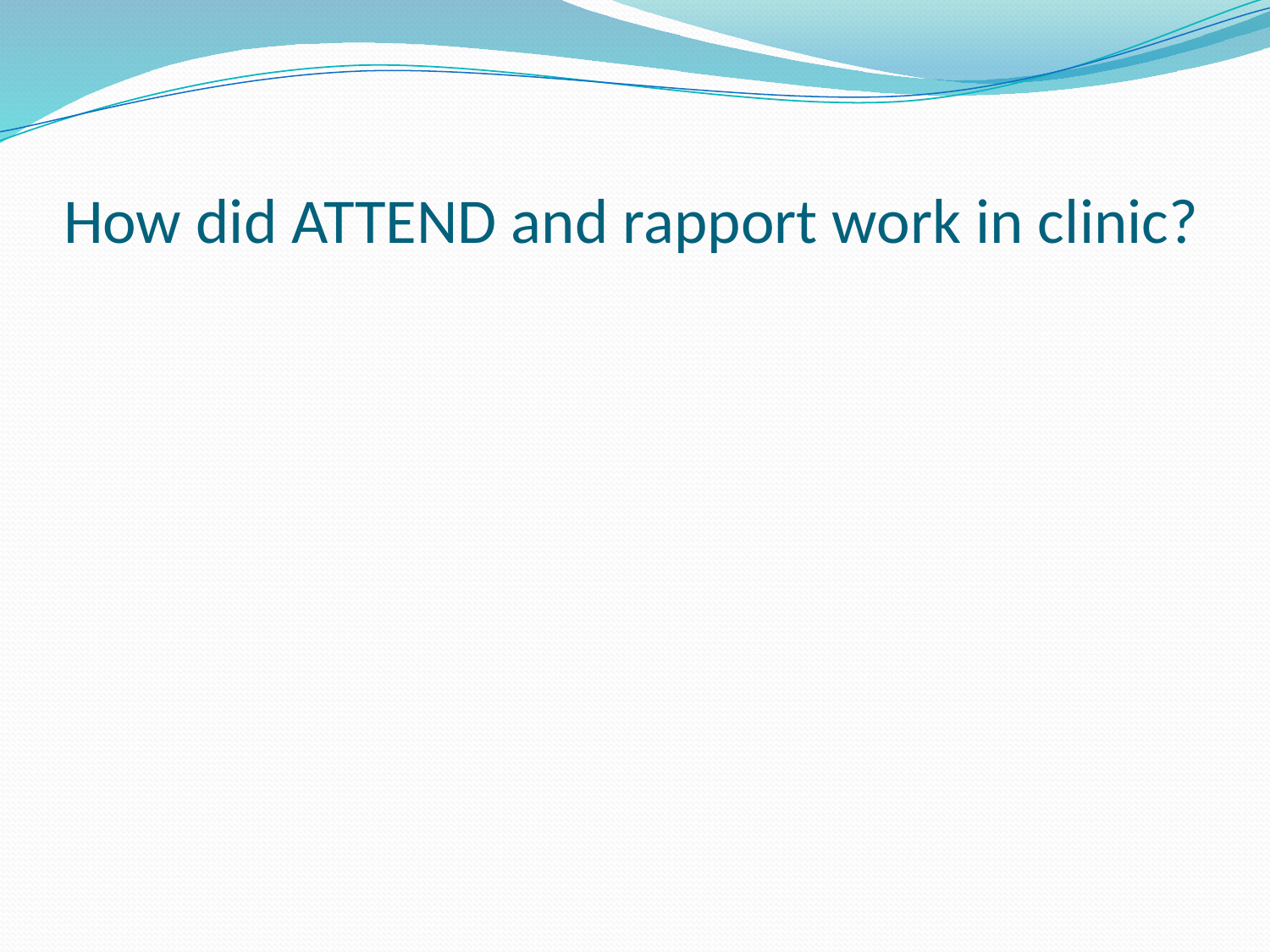

# How did ATTEND and rapport work in clinic?

## Slide 3
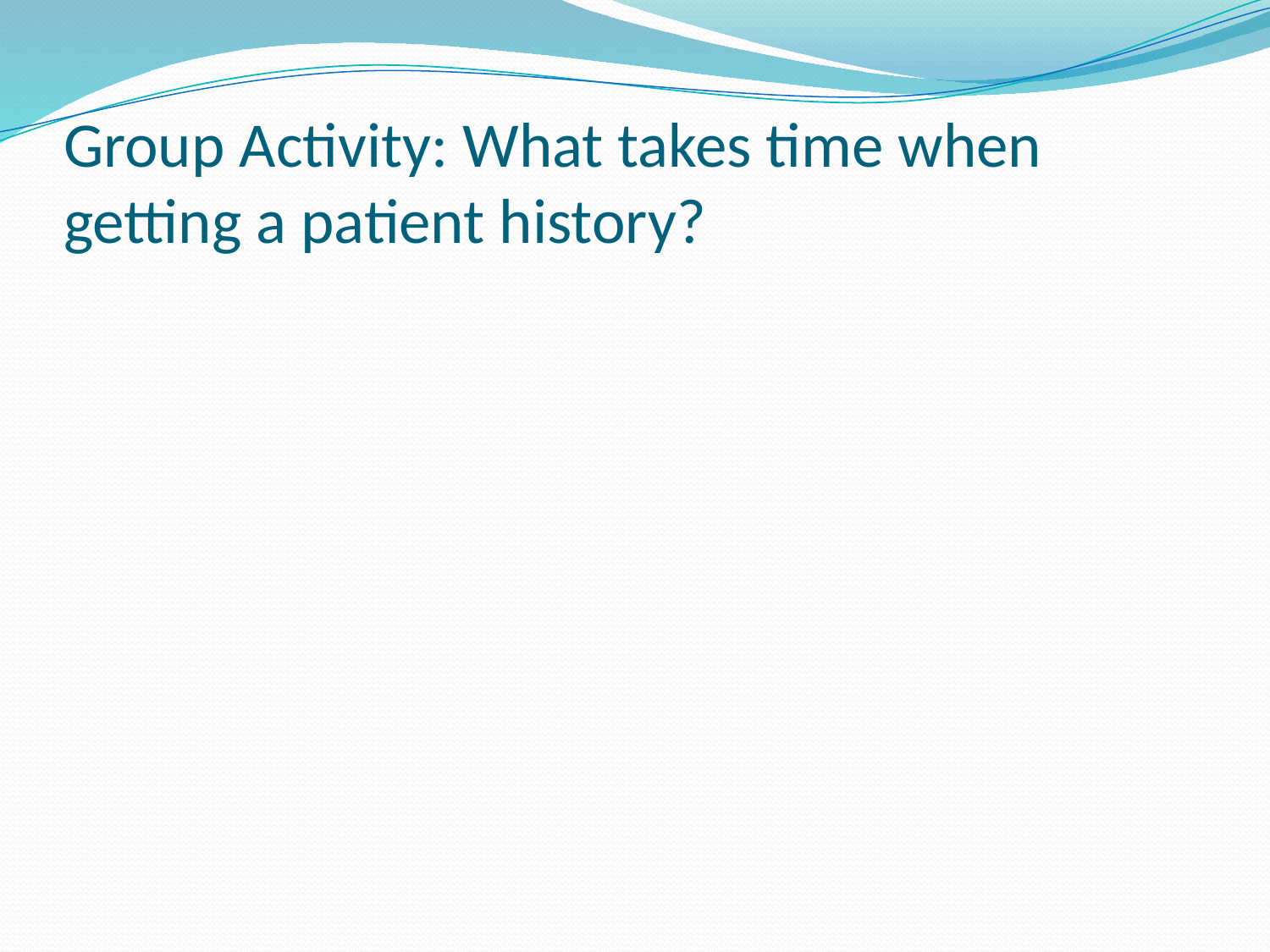

# Group Activity: What takes time when getting a patient history?

## Slide 4
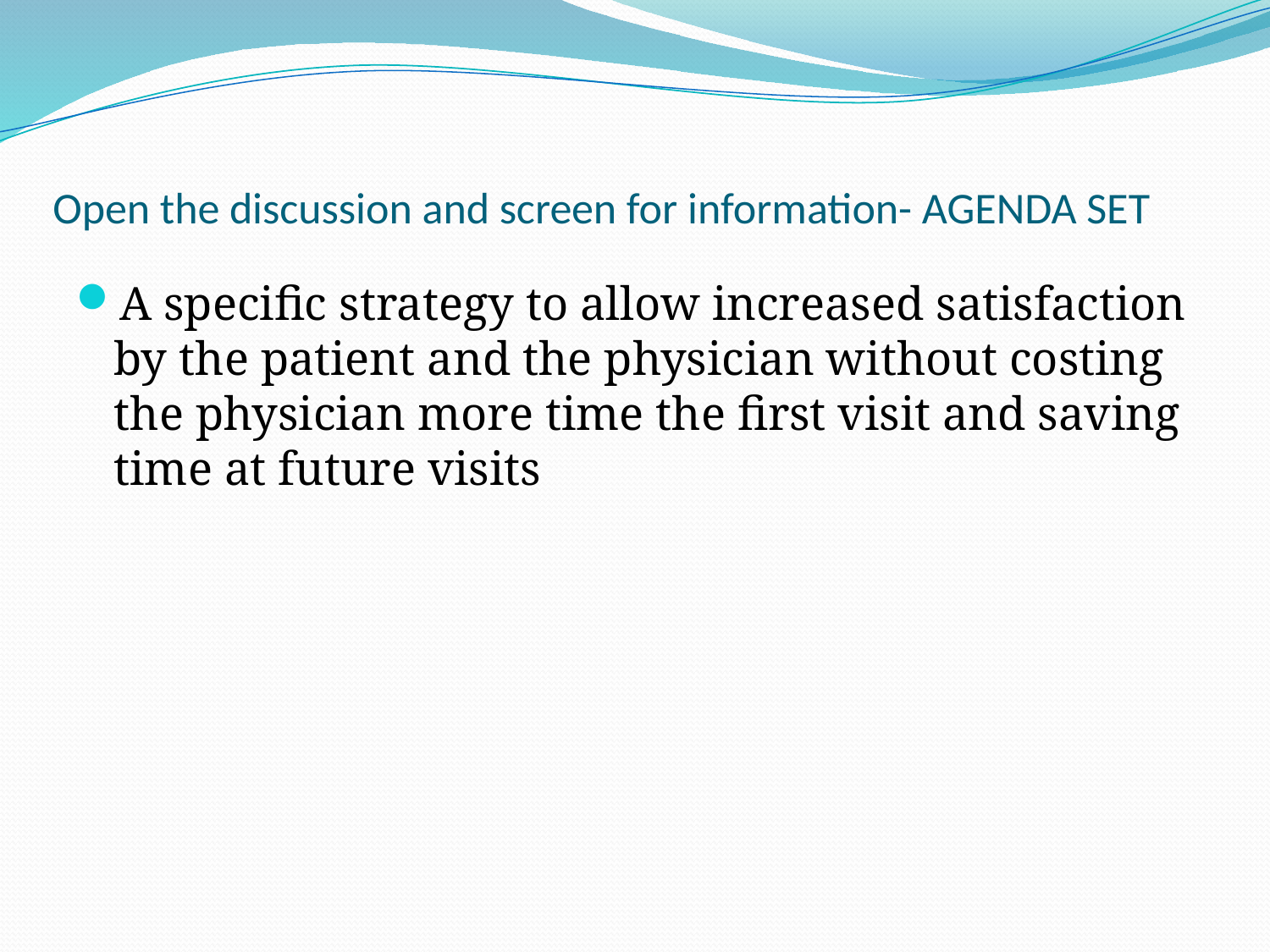

# Open the discussion and screen for information- AGENDA SET
A specific strategy to allow increased satisfaction by the patient and the physician without costing the physician more time the first visit and saving time at future visits

## Slide 5
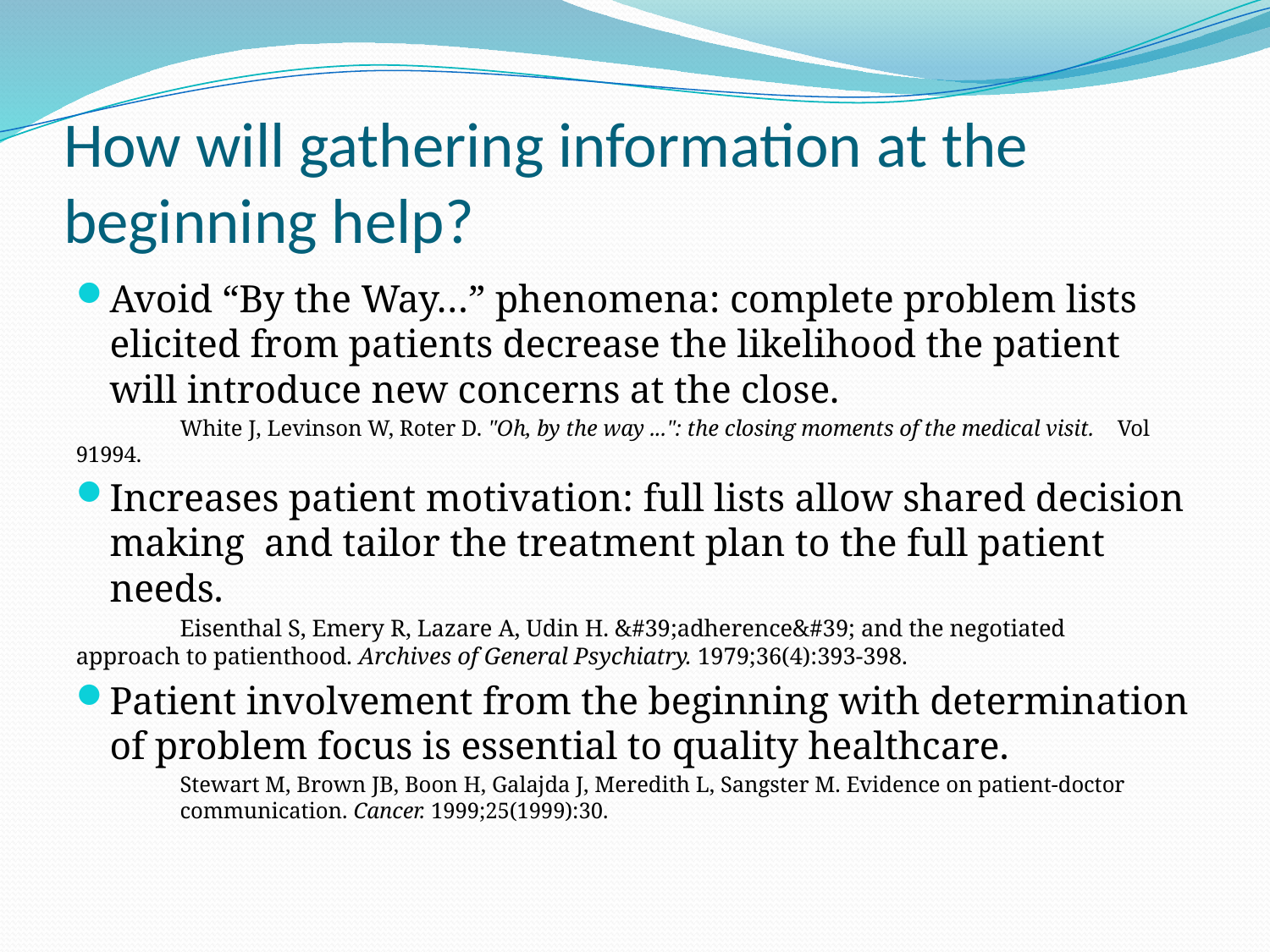

# How will gathering information at the beginning help?
Avoid “By the Way…” phenomena: complete problem lists elicited from patients decrease the likelihood the patient will introduce new concerns at the close.
	White J, Levinson W, Roter D. "Oh, by the way ...": the closing moments of the medical visit. 	Vol 91994.
Increases patient motivation: full lists allow shared decision making and tailor the treatment plan to the full patient needs.
	Eisenthal S, Emery R, Lazare A, Udin H. &#39;adherence&#39; and the negotiated 	approach to patienthood. Archives of General Psychiatry. 1979;36(4):393-398.
Patient involvement from the beginning with determination of problem focus is essential to quality healthcare.
	Stewart M, Brown JB, Boon H, Galajda J, Meredith L, Sangster M. Evidence on patient-doctor 	communication. Cancer. 1999;25(1999):30.

## Slide 6
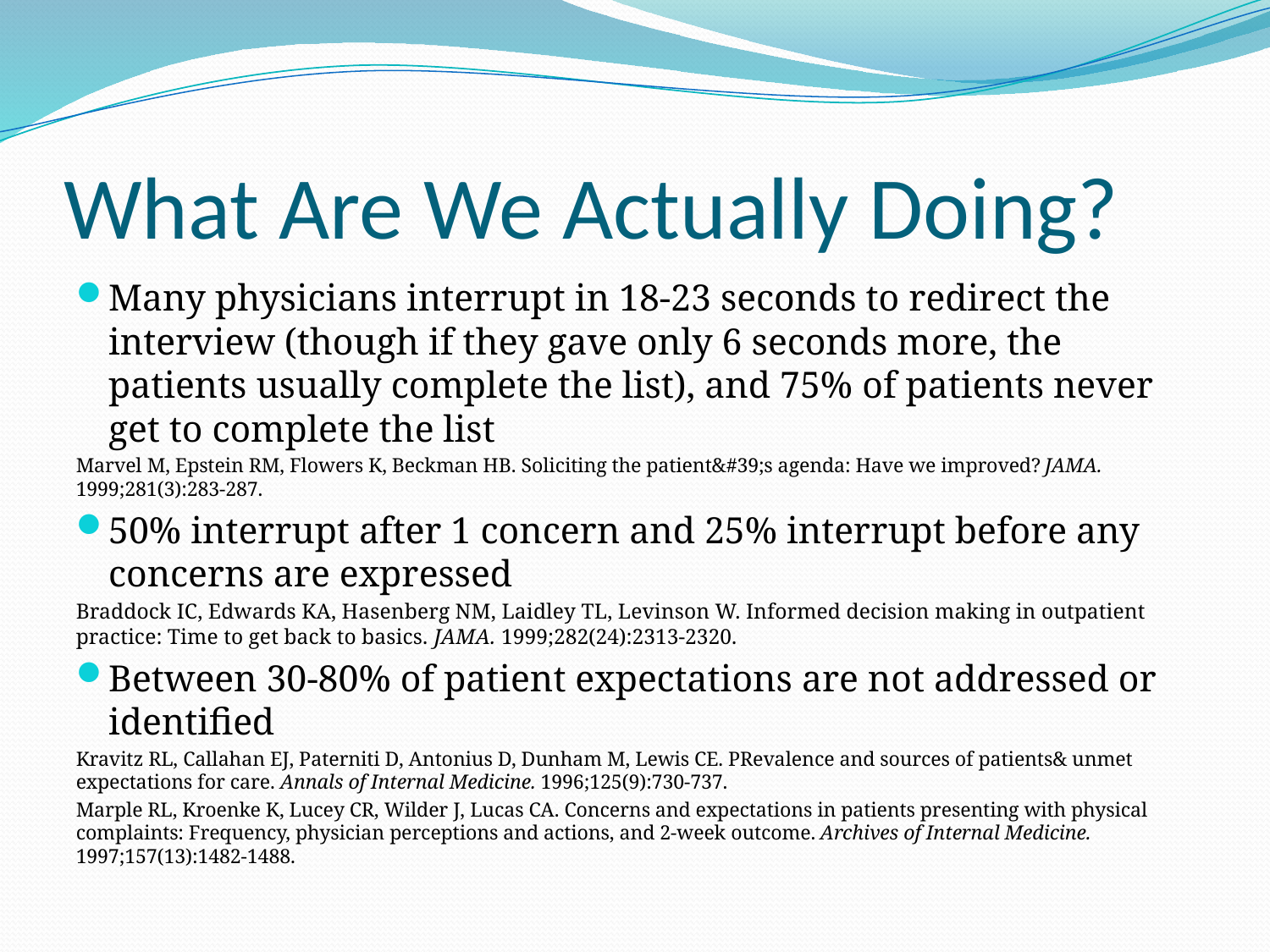

# What Are We Actually Doing?
Many physicians interrupt in 18-23 seconds to redirect the interview (though if they gave only 6 seconds more, the patients usually complete the list), and 75% of patients never get to complete the list
Marvel M, Epstein RM, Flowers K, Beckman HB. Soliciting the patient&#39;s agenda: Have we improved? JAMA. 1999;281(3):283-287.
50% interrupt after 1 concern and 25% interrupt before any concerns are expressed
Braddock IC, Edwards KA, Hasenberg NM, Laidley TL, Levinson W. Informed decision making in outpatient practice: Time to get back to basics. JAMA. 1999;282(24):2313-2320.
Between 30-80% of patient expectations are not addressed or identified
Kravitz RL, Callahan EJ, Paterniti D, Antonius D, Dunham M, Lewis CE. PRevalence and sources of patients& unmet expectations for care. Annals of Internal Medicine. 1996;125(9):730-737.
Marple RL, Kroenke K, Lucey CR, Wilder J, Lucas CA. Concerns and expectations in patients presenting with physical complaints: Frequency, physician perceptions and actions, and 2-week outcome. Archives of Internal Medicine. 1997;157(13):1482-1488.

## Slide 7
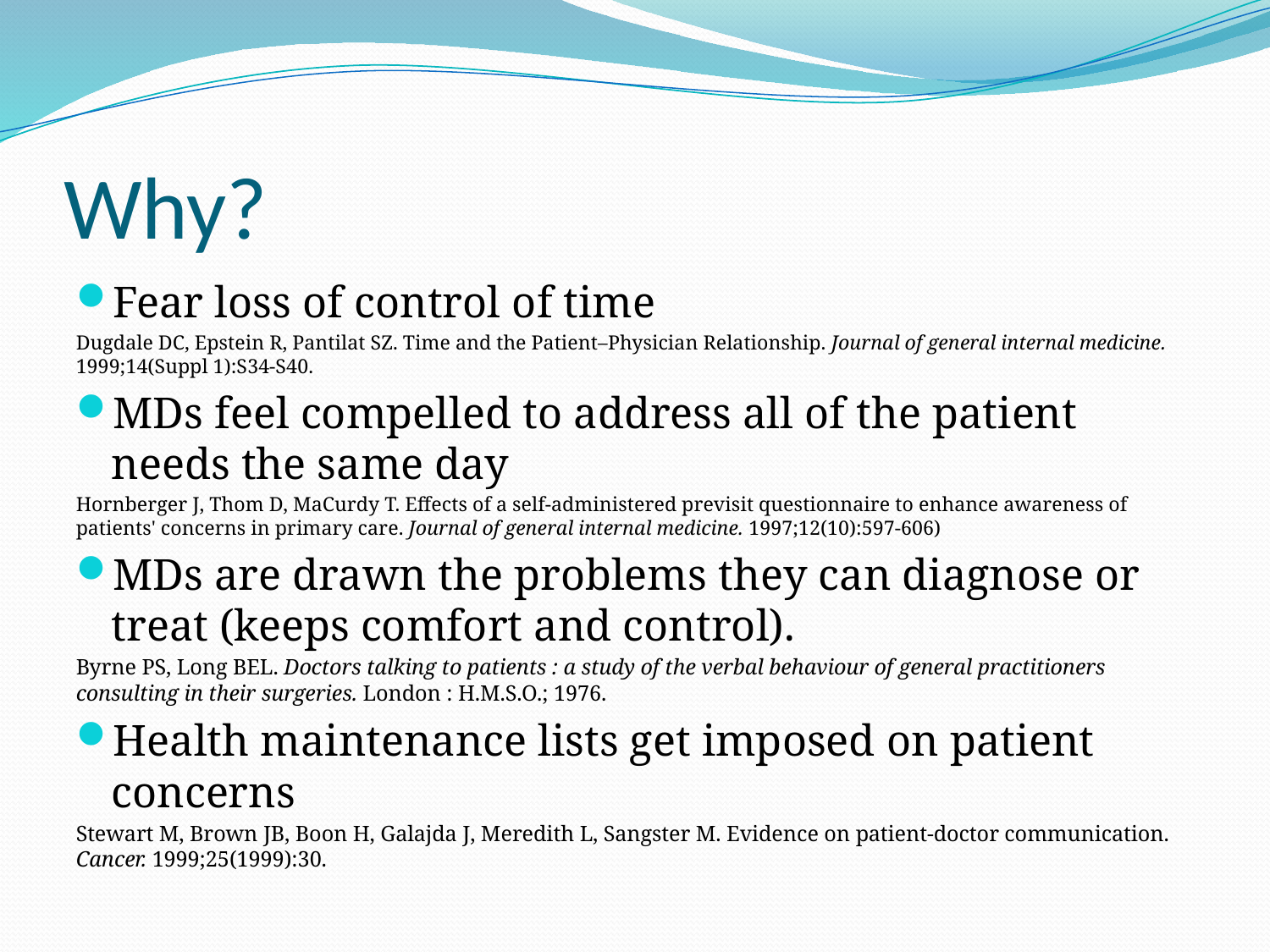

# Why?
Fear loss of control of time
Dugdale DC, Epstein R, Pantilat SZ. Time and the Patient–Physician Relationship. Journal of general internal medicine. 1999;14(Suppl 1):S34-S40.
MDs feel compelled to address all of the patient needs the same day
Hornberger J, Thom D, MaCurdy T. Effects of a self-administered previsit questionnaire to enhance awareness of patients' concerns in primary care. Journal of general internal medicine. 1997;12(10):597-606)
MDs are drawn the problems they can diagnose or treat (keeps comfort and control).
Byrne PS, Long BEL. Doctors talking to patients : a study of the verbal behaviour of general practitioners consulting in their surgeries. London : H.M.S.O.; 1976.
Health maintenance lists get imposed on patient concerns
Stewart M, Brown JB, Boon H, Galajda J, Meredith L, Sangster M. Evidence on patient-doctor communication. Cancer. 1999;25(1999):30.

## Slide 8
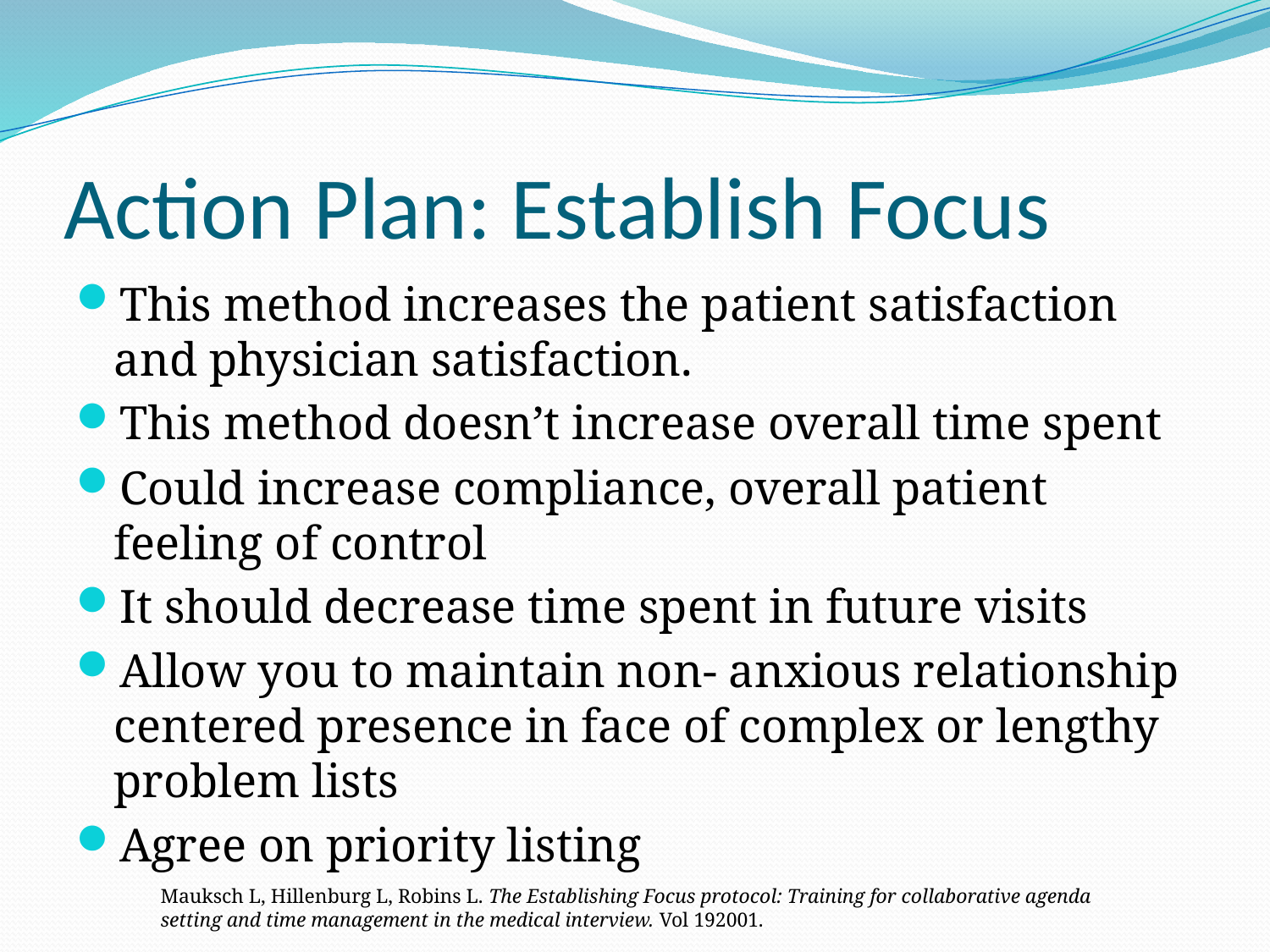

# Action Plan: Establish Focus
This method increases the patient satisfaction and physician satisfaction.
This method doesn’t increase overall time spent
Could increase compliance, overall patient feeling of control
It should decrease time spent in future visits
Allow you to maintain non- anxious relationship centered presence in face of complex or lengthy problem lists
Agree on priority listing
Mauksch L, Hillenburg L, Robins L. The Establishing Focus protocol: Training for collaborative agenda setting and time management in the medical interview. Vol 192001.

## Slide 9
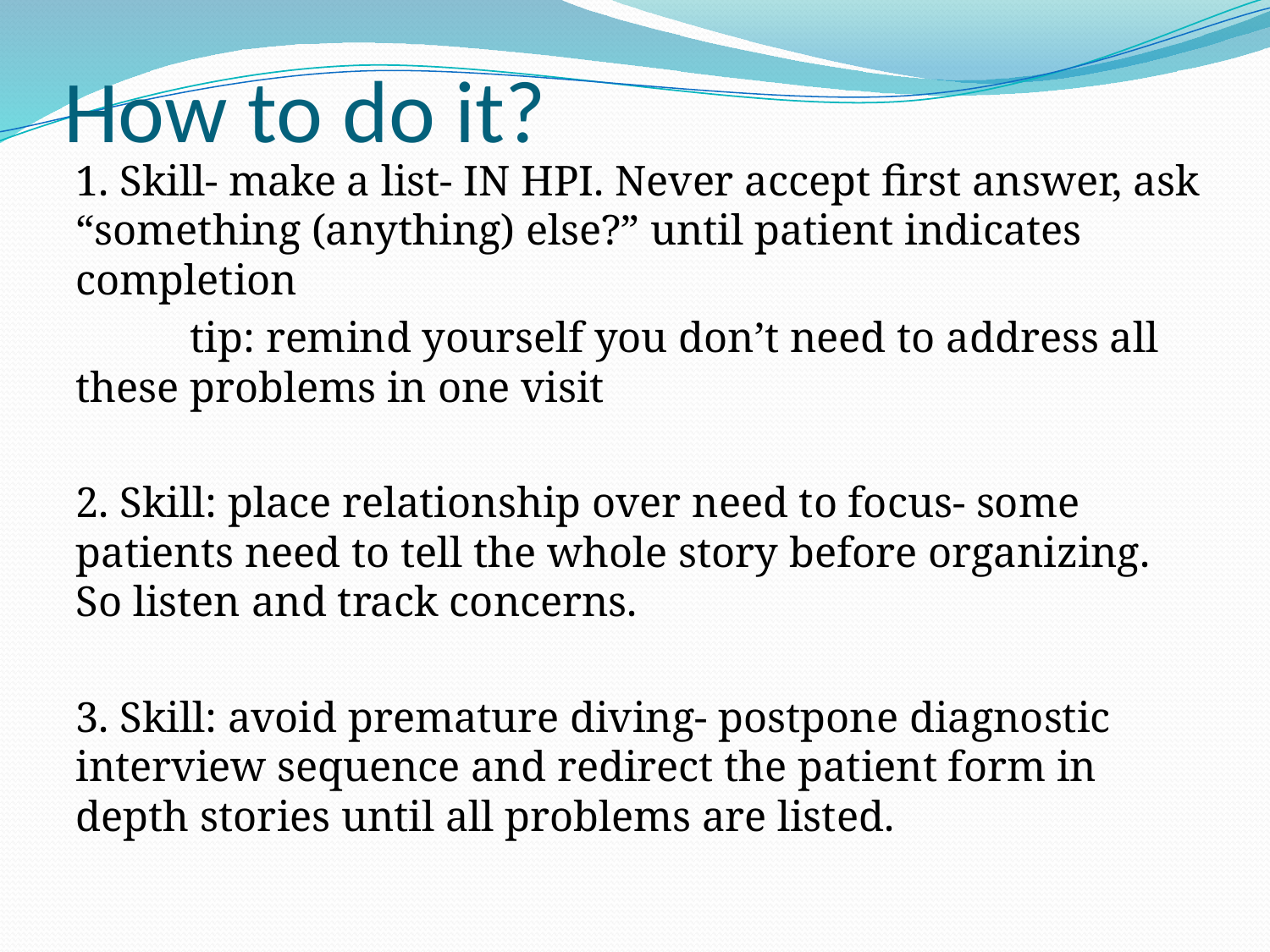

# How to do it?
1. Skill- make a list- IN HPI. Never accept first answer, ask “something (anything) else?” until patient indicates completion
	tip: remind yourself you don’t need to address all these problems in one visit
2. Skill: place relationship over need to focus- some patients need to tell the whole story before organizing. So listen and track concerns.
3. Skill: avoid premature diving- postpone diagnostic interview sequence and redirect the patient form in depth stories until all problems are listed.

## Slide 10
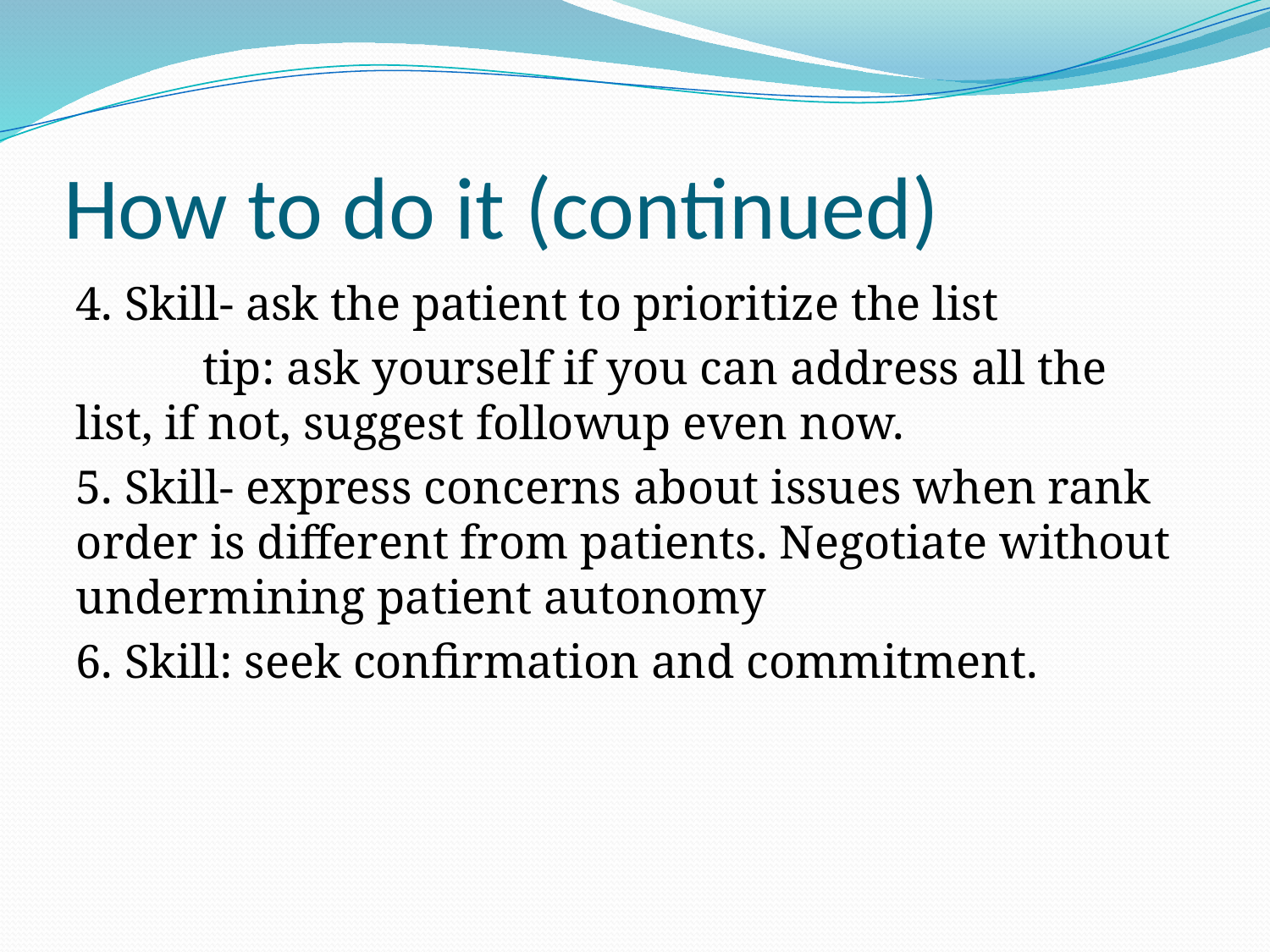

# How to do it (continued)
4. Skill- ask the patient to prioritize the list
	tip: ask yourself if you can address all the list, if not, suggest followup even now.
5. Skill- express concerns about issues when rank order is different from patients. Negotiate without undermining patient autonomy
6. Skill: seek confirmation and commitment.

## Slide 11
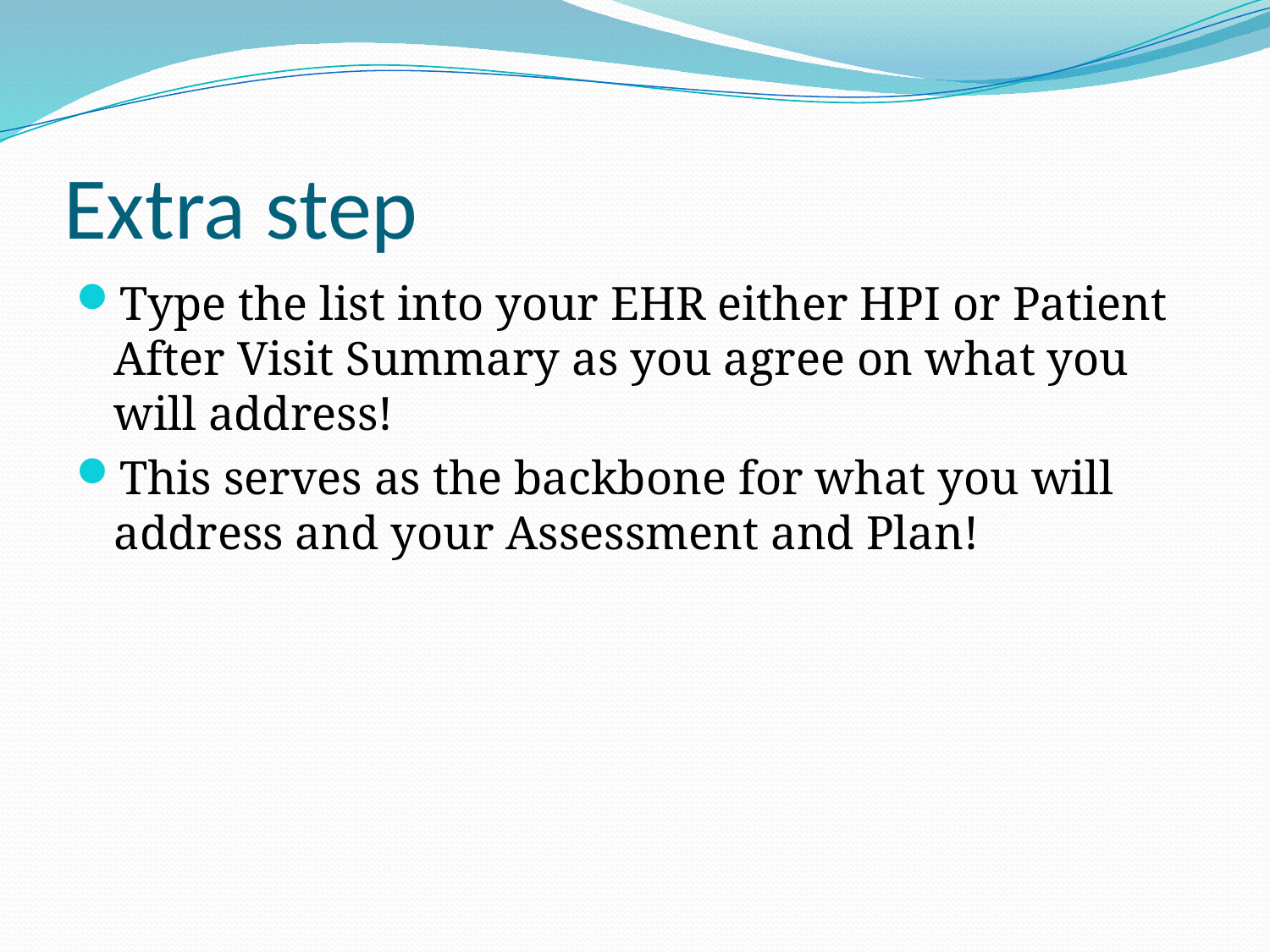

# Extra step
Type the list into your EHR either HPI or Patient After Visit Summary as you agree on what you will address!
This serves as the backbone for what you will address and your Assessment and Plan!

## Slide 12
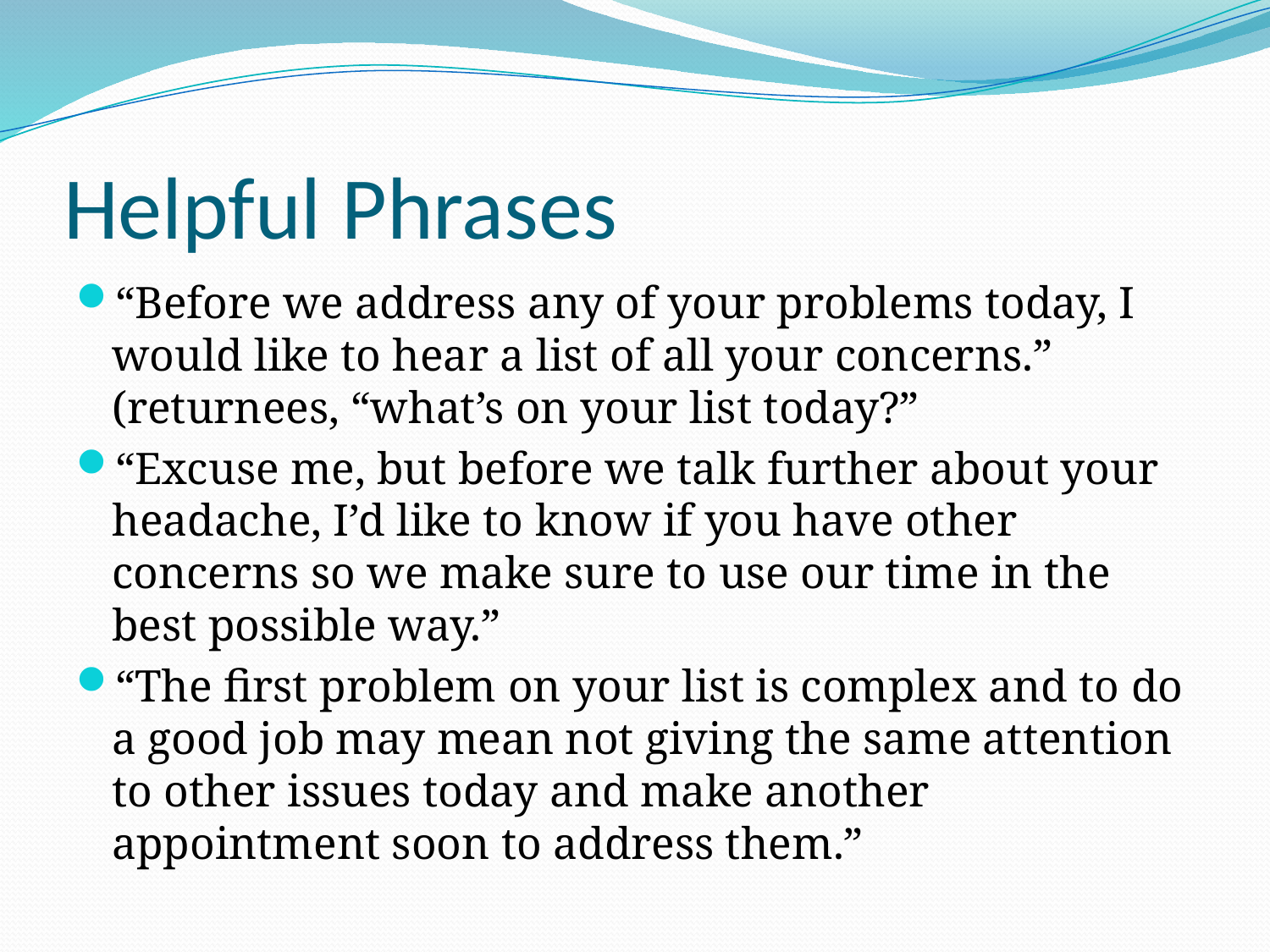

# Helpful Phrases
“Before we address any of your problems today, I would like to hear a list of all your concerns.” (returnees, “what’s on your list today?”
“Excuse me, but before we talk further about your headache, I’d like to know if you have other concerns so we make sure to use our time in the best possible way.”
“The first problem on your list is complex and to do a good job may mean not giving the same attention to other issues today and make another appointment soon to address them.”

## Slide 13
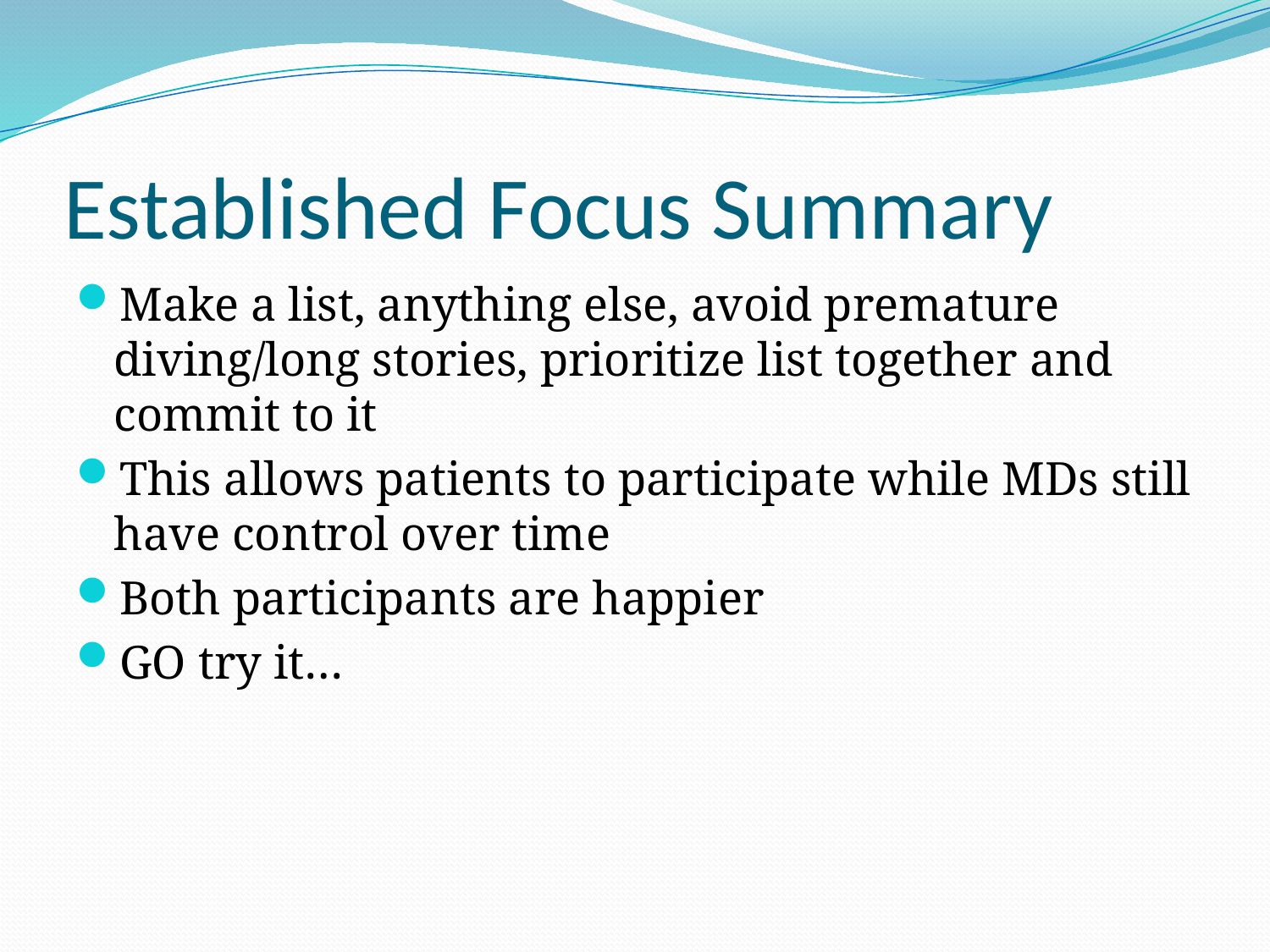

# Established Focus Summary
Make a list, anything else, avoid premature diving/long stories, prioritize list together and commit to it
This allows patients to participate while MDs still have control over time
Both participants are happier
GO try it…

## Slide 14
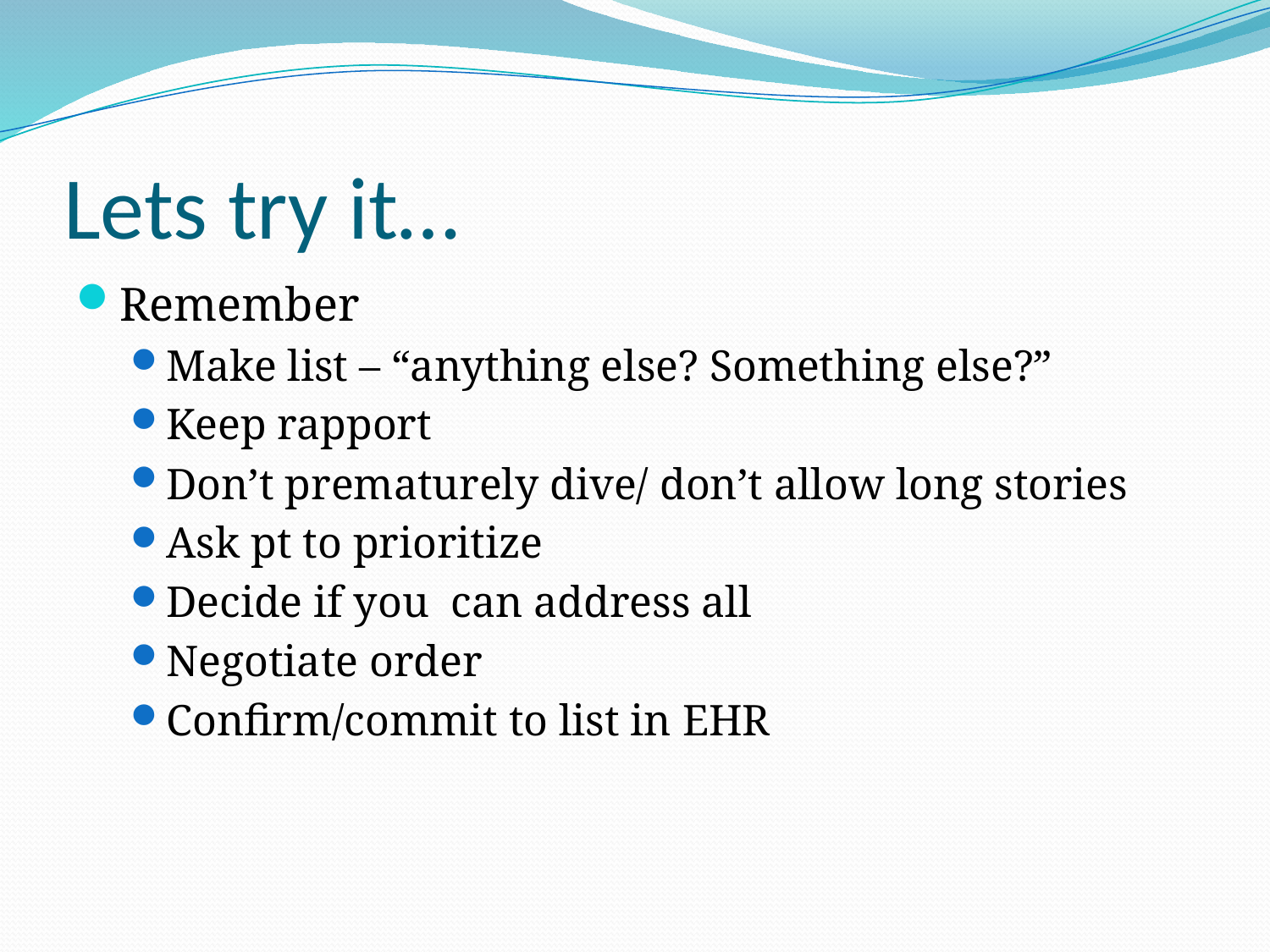

# Lets try it…
Remember
Make list – “anything else? Something else?”
Keep rapport
Don’t prematurely dive/ don’t allow long stories
Ask pt to prioritize
Decide if you can address all
Negotiate order
Confirm/commit to list in EHR

## Slide 15
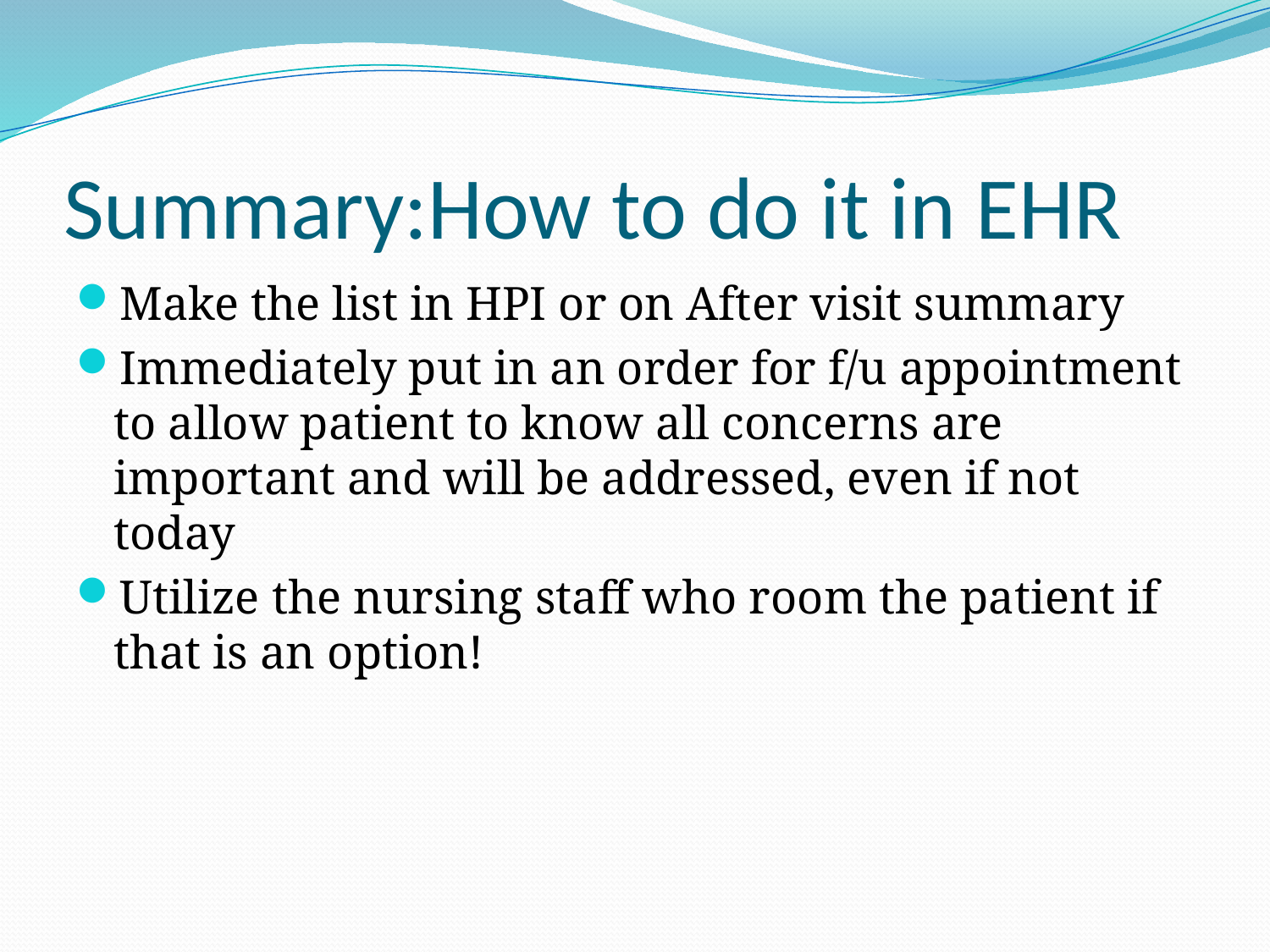

# Summary:How to do it in EHR
Make the list in HPI or on After visit summary
Immediately put in an order for f/u appointment to allow patient to know all concerns are important and will be addressed, even if not today
Utilize the nursing staff who room the patient if that is an option!

## Slide 16
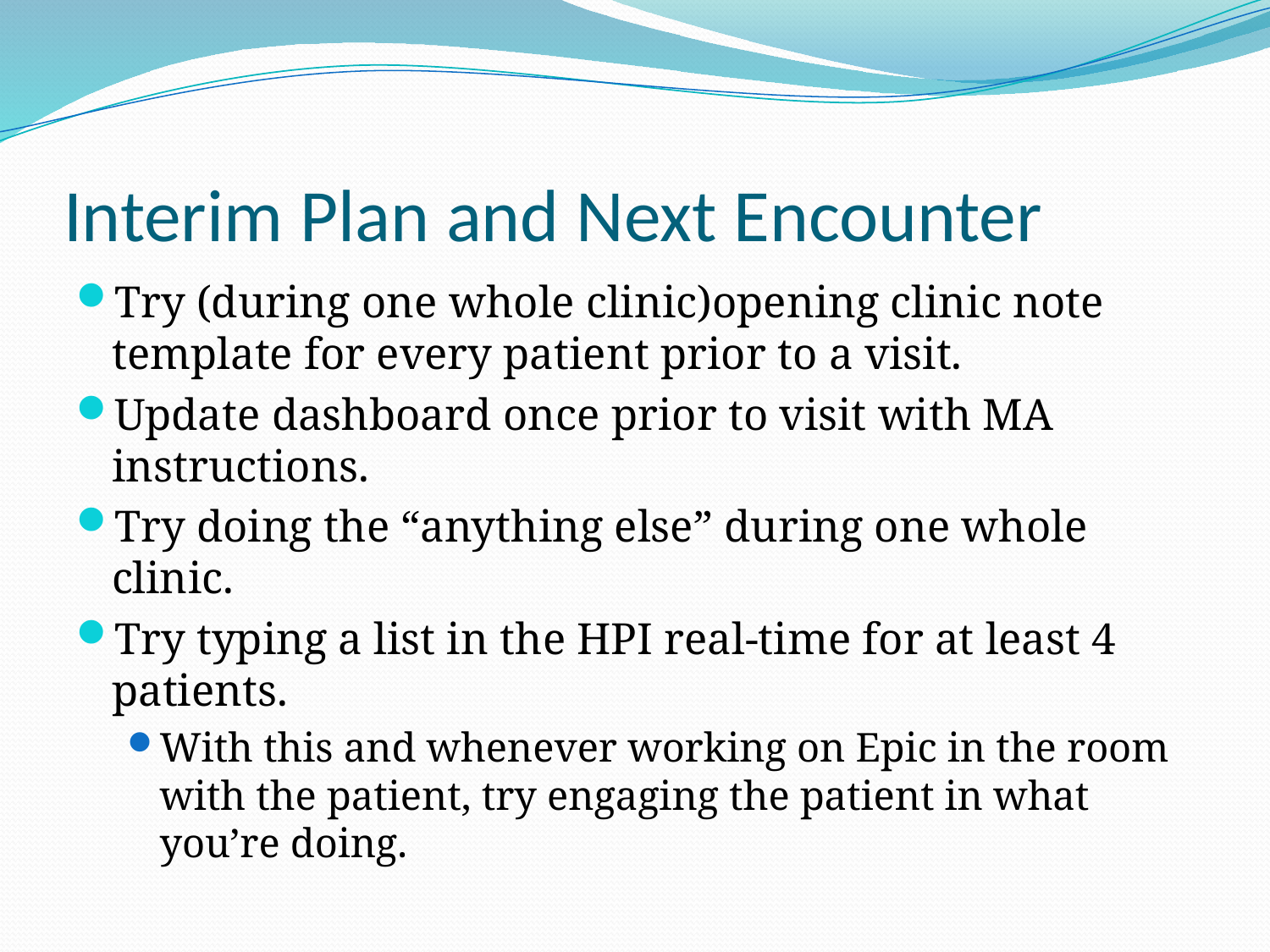

# Interim Plan and Next Encounter
Try (during one whole clinic)opening clinic note template for every patient prior to a visit.
Update dashboard once prior to visit with MA instructions.
Try doing the “anything else” during one whole clinic.
Try typing a list in the HPI real-time for at least 4 patients.
With this and whenever working on Epic in the room with the patient, try engaging the patient in what you’re doing.

## Slide 17
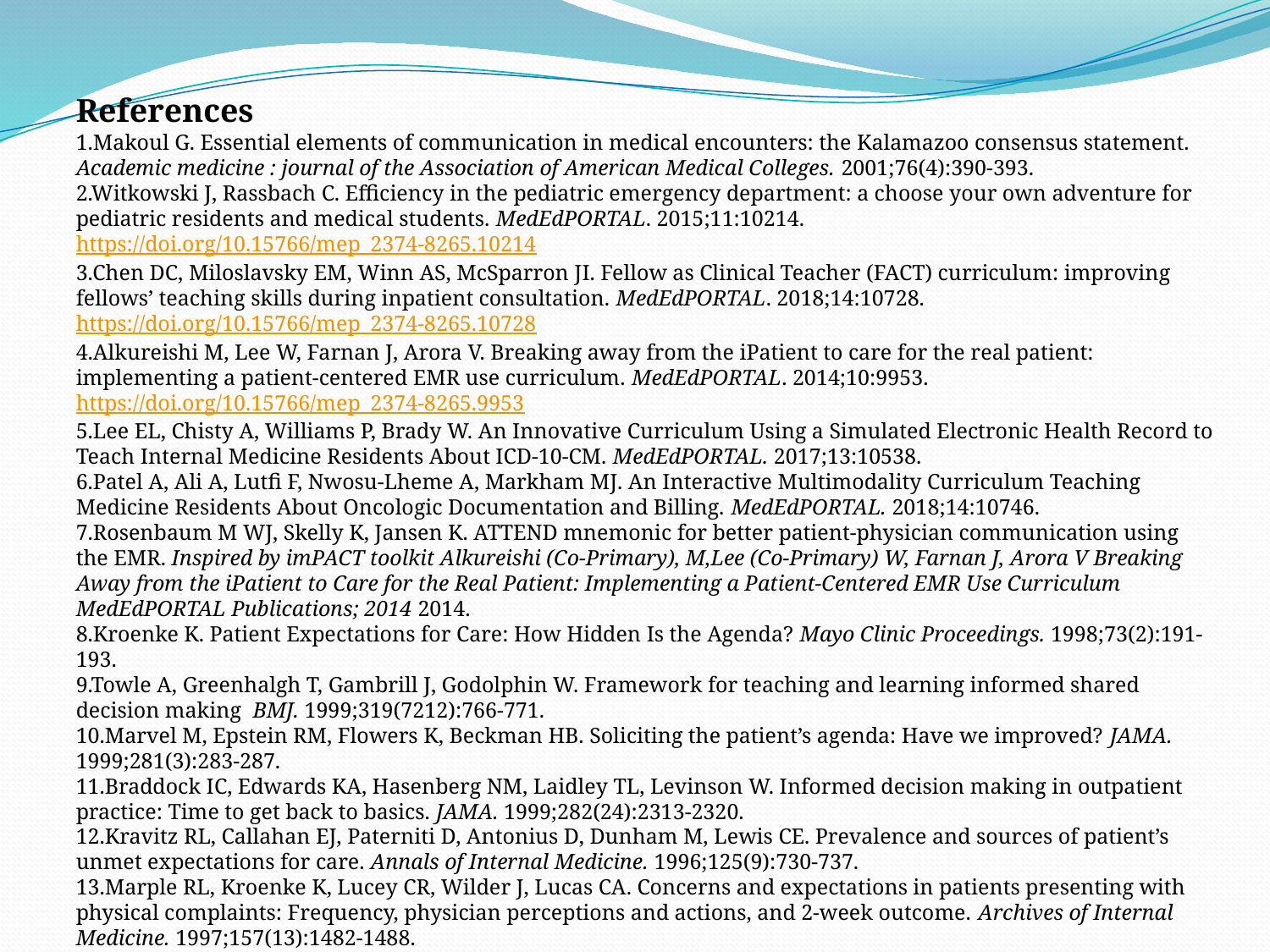

References
1.Makoul G. Essential elements of communication in medical encounters: the Kalamazoo consensus statement. Academic medicine : journal of the Association of American Medical Colleges. 2001;76(4):390-393.
2.Witkowski J, Rassbach C. Efficiency in the pediatric emergency department: a choose your own adventure for pediatric residents and medical students. MedEdPORTAL. 2015;11:10214. https://doi.org/10.15766/mep_2374-8265.10214
3.Chen DC, Miloslavsky EM, Winn AS, McSparron JI. Fellow as Clinical Teacher (FACT) curriculum: improving fellows’ teaching skills during inpatient consultation. MedEdPORTAL. 2018;14:10728. https://doi.org/10.15766/mep_2374-8265.10728
4.Alkureishi M, Lee W, Farnan J, Arora V. Breaking away from the iPatient to care for the real patient: implementing a patient-centered EMR use curriculum. MedEdPORTAL. 2014;10:9953. https://doi.org/10.15766/mep_2374-8265.9953
5.Lee EL, Chisty A, Williams P, Brady W. An Innovative Curriculum Using a Simulated Electronic Health Record to Teach Internal Medicine Residents About ICD-10-CM. MedEdPORTAL. 2017;13:10538.
6.Patel A, Ali A, Lutfi F, Nwosu-Lheme A, Markham MJ. An Interactive Multimodality Curriculum Teaching Medicine Residents About Oncologic Documentation and Billing. MedEdPORTAL. 2018;14:10746.
7.Rosenbaum M WJ, Skelly K, Jansen K. ATTEND mnemonic for better patient-physician communication using the EMR. Inspired by imPACT toolkit Alkureishi (Co-Primary), M,Lee (Co-Primary) W, Farnan J, Arora V Breaking Away from the iPatient to Care for the Real Patient: Implementing a Patient-Centered EMR Use Curriculum MedEdPORTAL Publications; 2014 2014.
8.Kroenke K. Patient Expectations for Care: How Hidden Is the Agenda? Mayo Clinic Proceedings. 1998;73(2):191-193.
9.Towle A, Greenhalgh T, Gambrill J, Godolphin W. Framework for teaching and learning informed shared decision making BMJ. 1999;319(7212):766-771.
10.Marvel M, Epstein RM, Flowers K, Beckman HB. Soliciting the patient’s agenda: Have we improved? JAMA. 1999;281(3):283-287.
11.Braddock IC, Edwards KA, Hasenberg NM, Laidley TL, Levinson W. Informed decision making in outpatient practice: Time to get back to basics. JAMA. 1999;282(24):2313-2320.
12.Kravitz RL, Callahan EJ, Paterniti D, Antonius D, Dunham M, Lewis CE. Prevalence and sources of patient’s unmet expectations for care. Annals of Internal Medicine. 1996;125(9):730-737.
13.Marple RL, Kroenke K, Lucey CR, Wilder J, Lucas CA. Concerns and expectations in patients presenting with physical complaints: Frequency, physician perceptions and actions, and 2-week outcome. Archives of Internal Medicine. 1997;157(13):1482-1488.
14.Mauksch L, Hillenburg L, Robins L. The Establishing Focus protocol: Training for collaborative agenda setting and time management in the medical interview. Vol 192001.
15.Østbye T, Yarnall KSH, Krause KM, Pollak KI, Gradison M, Michener JL. Is There Time for Management of Patients With Chronic Diseases in Primary Care? The Annals of Family Medicine. 2005;3(3):209-214.

## Slide 18
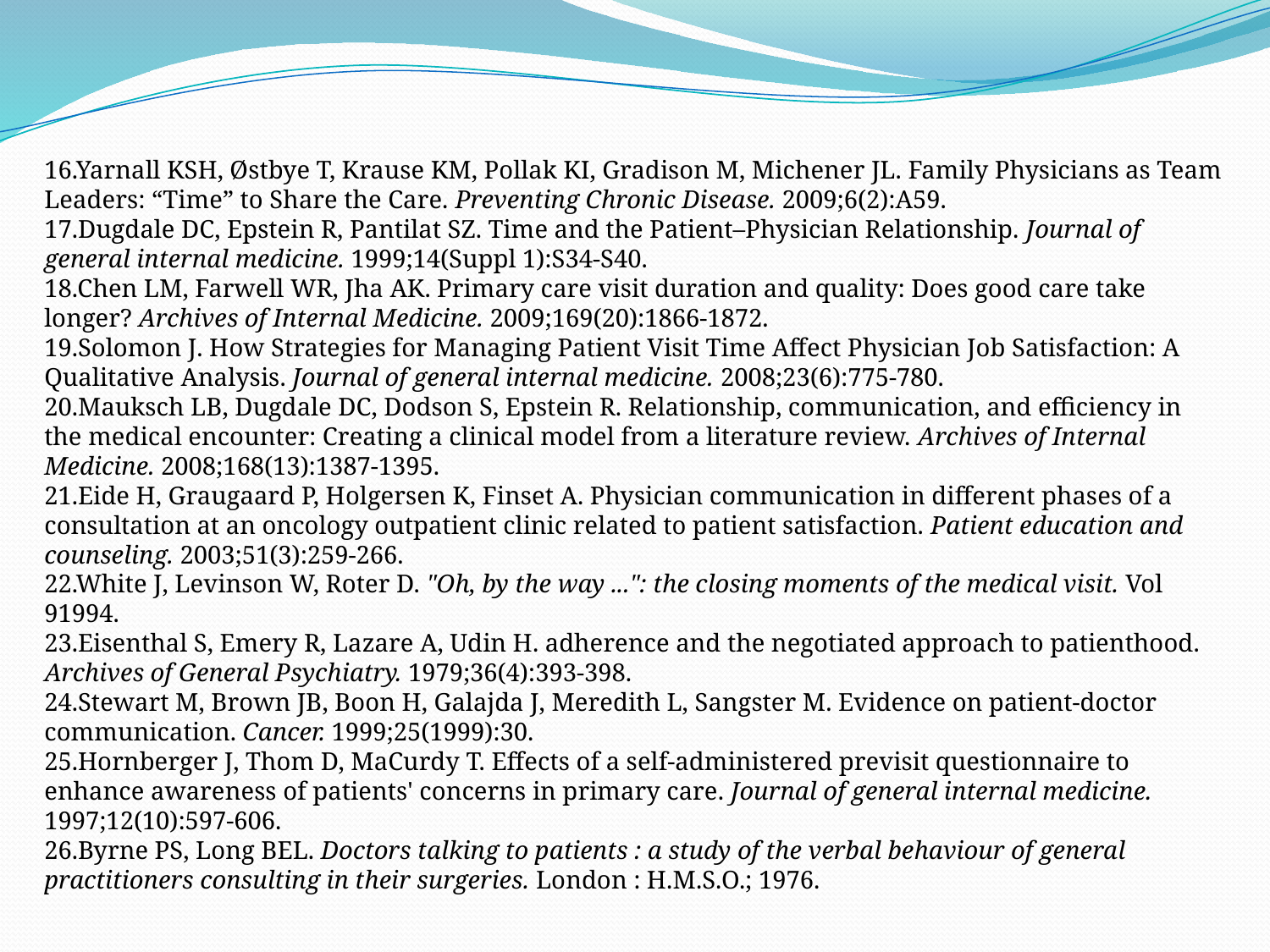

16.Yarnall KSH, Østbye T, Krause KM, Pollak KI, Gradison M, Michener JL. Family Physicians as Team Leaders: “Time” to Share the Care. Preventing Chronic Disease. 2009;6(2):A59.
17.Dugdale DC, Epstein R, Pantilat SZ. Time and the Patient–Physician Relationship. Journal of general internal medicine. 1999;14(Suppl 1):S34-S40.
18.Chen LM, Farwell WR, Jha AK. Primary care visit duration and quality: Does good care take longer? Archives of Internal Medicine. 2009;169(20):1866-1872.
19.Solomon J. How Strategies for Managing Patient Visit Time Affect Physician Job Satisfaction: A Qualitative Analysis. Journal of general internal medicine. 2008;23(6):775-780.
20.Mauksch LB, Dugdale DC, Dodson S, Epstein R. Relationship, communication, and efficiency in the medical encounter: Creating a clinical model from a literature review. Archives of Internal Medicine. 2008;168(13):1387-1395.
21.Eide H, Graugaard P, Holgersen K, Finset A. Physician communication in different phases of a consultation at an oncology outpatient clinic related to patient satisfaction. Patient education and counseling. 2003;51(3):259-266.
22.White J, Levinson W, Roter D. "Oh, by the way ...": the closing moments of the medical visit. Vol 91994.
23.Eisenthal S, Emery R, Lazare A, Udin H. adherence and the negotiated approach to patienthood. Archives of General Psychiatry. 1979;36(4):393-398.
24.Stewart M, Brown JB, Boon H, Galajda J, Meredith L, Sangster M. Evidence on patient-doctor communication. Cancer. 1999;25(1999):30.
25.Hornberger J, Thom D, MaCurdy T. Effects of a self-administered previsit questionnaire to enhance awareness of patients' concerns in primary care. Journal of general internal medicine. 1997;12(10):597-606.
26.Byrne PS, Long BEL. Doctors talking to patients : a study of the verbal behaviour of general practitioners consulting in their surgeries. London : H.M.S.O.; 1976.
