# Supplementary material for: A Curriculum for Teaching Clinical Efficiency Focusing on Specific Communication Skills While Maximizing the Electronic Health Record
Source: MedEdPORTAL. 2020 Oct 29;16:10989. doi: 10.15766/mep_2374-8265.10989 (PMC7597939; doi:10.15766/mep_2374-8265.10989)
Supplement: Supplementary file 1 — Efficiency Preworkshop Needs Assessment Survey.docxWorkshop 1 - Setting up the Template and Working in EHR.pptxSample Clinic Note and AVS Template.docxWorkshop 2 - Preclinic Preparation and Rapport Building.pptxEfficiency ATTEND Practice Card.docxWorkshop 3 - Agenda Setting and Relationship Maintenance.pptxEfficiency Agenda Setting Practice.docxWorkshop 4 - Visit Closure.pptxEfficiency Closure Card and Cases.docxEfficiency Postworkshop Evaluation.docx [file mep_2374-8265.10989-s001.zip › G. Efficiency Agenda Setting Practice.docx]

| **Establish Focus Method**   - Make List – “Anything Else? Something else?” - Keep Rapport - Don’t prematurely dive/don’t allow long stories - Ask pt to prioritize - Decide if you can address all (and if not, suggest follow-up even as prioritizing) - Negotiate order - Confirm/Commit to list | **Establish Focus Method**   - Make List – “Anything Else? Something else?” - Keep Rapport - Don’t prematurely dive/don’t allow long stories - Ask pt to prioritize - Decide if you can address all (and if not, suggest follow-up even as prioritizing) - Negotiate order - Confirm/Commit to list |
| --- | --- |
| **Establish Focus Method**   - Make List – “Anything Else? Something else?” - Keep Rapport - Don’t prematurely dive/don’t allow long stories - Ask pt to prioritize - Decide if you can address all (and if not, suggest follow-up even as prioritizing) - Negotiate order - Confirm/Commit to list | **Establish Focus Method**   - Make List – “Anything Else? Something else?” - Keep Rapport - Don’t prematurely dive/don’t allow long stories - Ask pt to prioritize - Decide if you can address all (and if not, suggest follow-up even as prioritizing) - Negotiate order - Confirm/Commit to list |
| **Helpful Phrases**  “Before we address any of your problems today, I would like to hear a list of all your concerns.” (Returnees: “What’s on your list today?”)  “Excuse me, before we talk further about your headache, I’d like to know if you have any other concerns so we can make sure to use our time in the best possible way.”  “The first problem on your list is complex, and to do a good job with it may mean not giving the same attention to other issues today.” | **Helpful Phrases**  “Before we address any of your problems today, I would like to hear a list of all your concerns.” (Returnees: “What’s on your list today?”)  “Excuse me, before we talk further about your headache, I’d like to know if you have any other concerns so we can make sure to use our time in the best possible way.”  “The first problem on your list is complex, and to do a good job with it may mean not giving the same attention to other issues today.” |
| **Helpful Phrases**  “Before we address any of your problems today, I would like to hear a list of all your concerns.” (Returnees: “What’s on your list today?”)  “Excuse me, before we talk further about your headache, I’d like to know if you have any other concerns so we can make sure to use our time in the best possible way.”  “The first problem on your list is complex, and to do a good job with it may mean not giving the same attention to other issues today.” | **Helpful Phrases**  “Before we address any of your problems today, I would like to hear a list of all your concerns.” (Returnees: “What’s on your list today?”)  “Excuse me, before we talk further about your headache, I’d like to know if you have any other concerns so we can make sure to use our time in the best possible way.”  “The first problem on your list is complex, and to do a good job with it may mean not giving the same attention to other issues today.” |
