# Supplementary material for: A Curriculum for Teaching Clinical Efficiency Focusing on Specific Communication Skills While Maximizing the Electronic Health Record
Source: MedEdPORTAL. 2020 Oct 29;16:10989. doi: 10.15766/mep_2374-8265.10989 (PMC7597939; doi:10.15766/mep_2374-8265.10989)
Supplement: Supplementary file 1 — Efficiency Preworkshop Needs Assessment Survey.docxWorkshop 1 - Setting up the Template and Working in EHR.pptxSample Clinic Note and AVS Template.docxWorkshop 2 - Preclinic Preparation and Rapport Building.pptxEfficiency ATTEND Practice Card.docxWorkshop 3 - Agenda Setting and Relationship Maintenance.pptxEfficiency Agenda Setting Practice.docxWorkshop 4 - Visit Closure.pptxEfficiency Closure Card and Cases.docxEfficiency Postworkshop Evaluation.docx [file mep_2374-8265.10989-s001.zip › H. Workshop 4 - Visit Closure.pptx]

## Slide 1
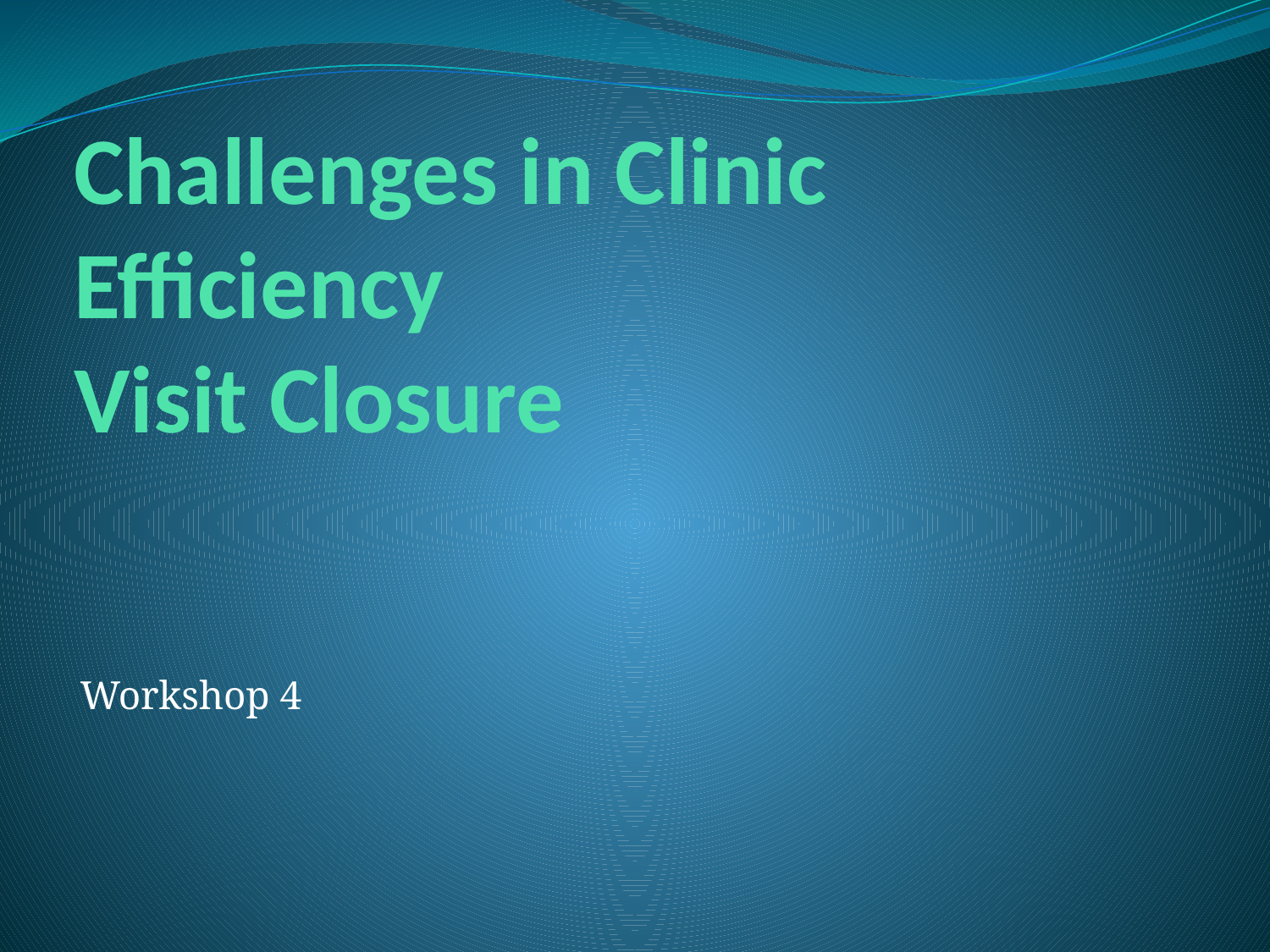

# Challenges in Clinic Efficiency Visit Closure
Workshop 4

## Slide 2
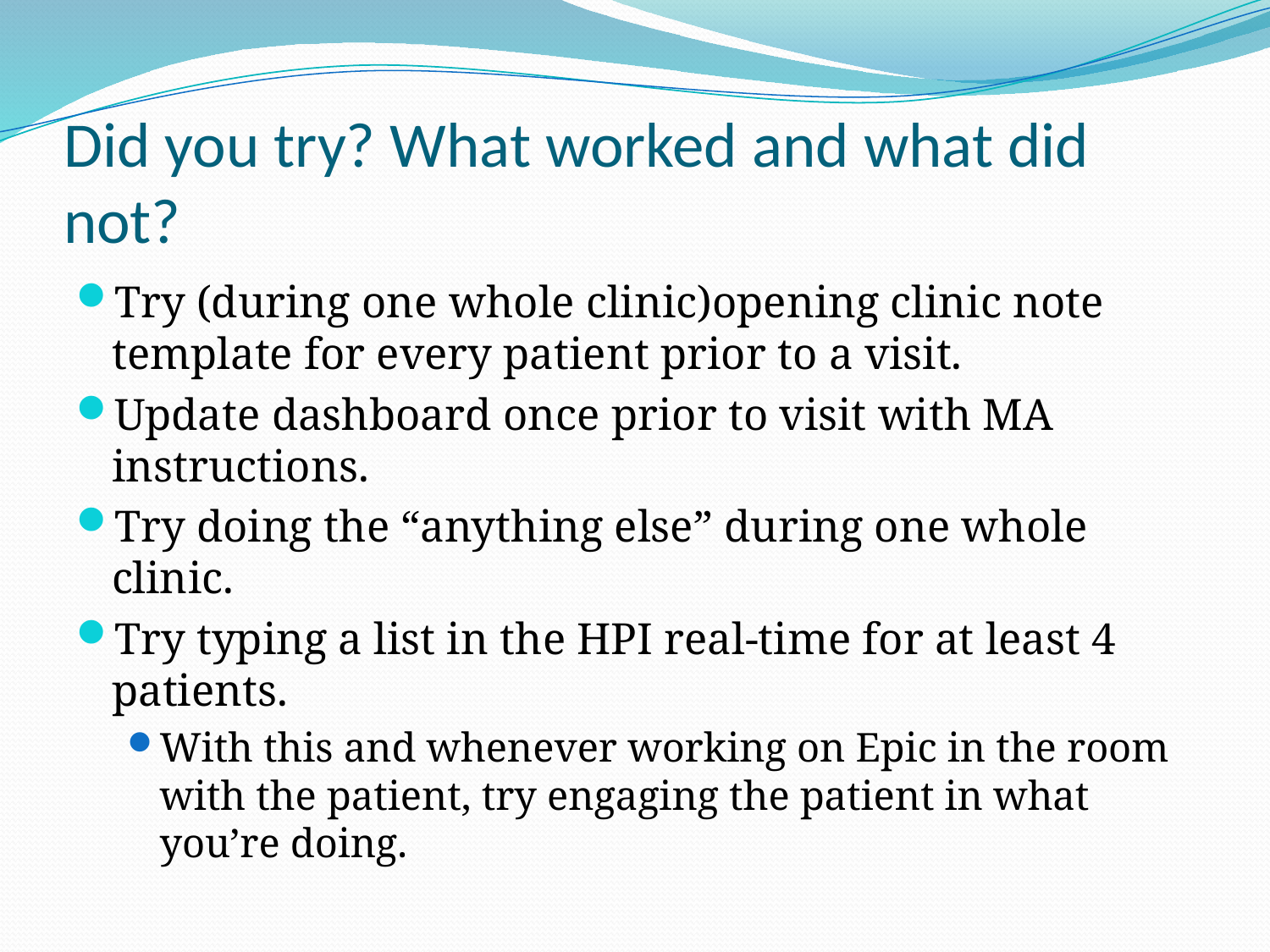

# Did you try? What worked and what did not?
Try (during one whole clinic)opening clinic note template for every patient prior to a visit.
Update dashboard once prior to visit with MA instructions.
Try doing the “anything else” during one whole clinic.
Try typing a list in the HPI real-time for at least 4 patients.
With this and whenever working on Epic in the room with the patient, try engaging the patient in what you’re doing.

## Slide 3
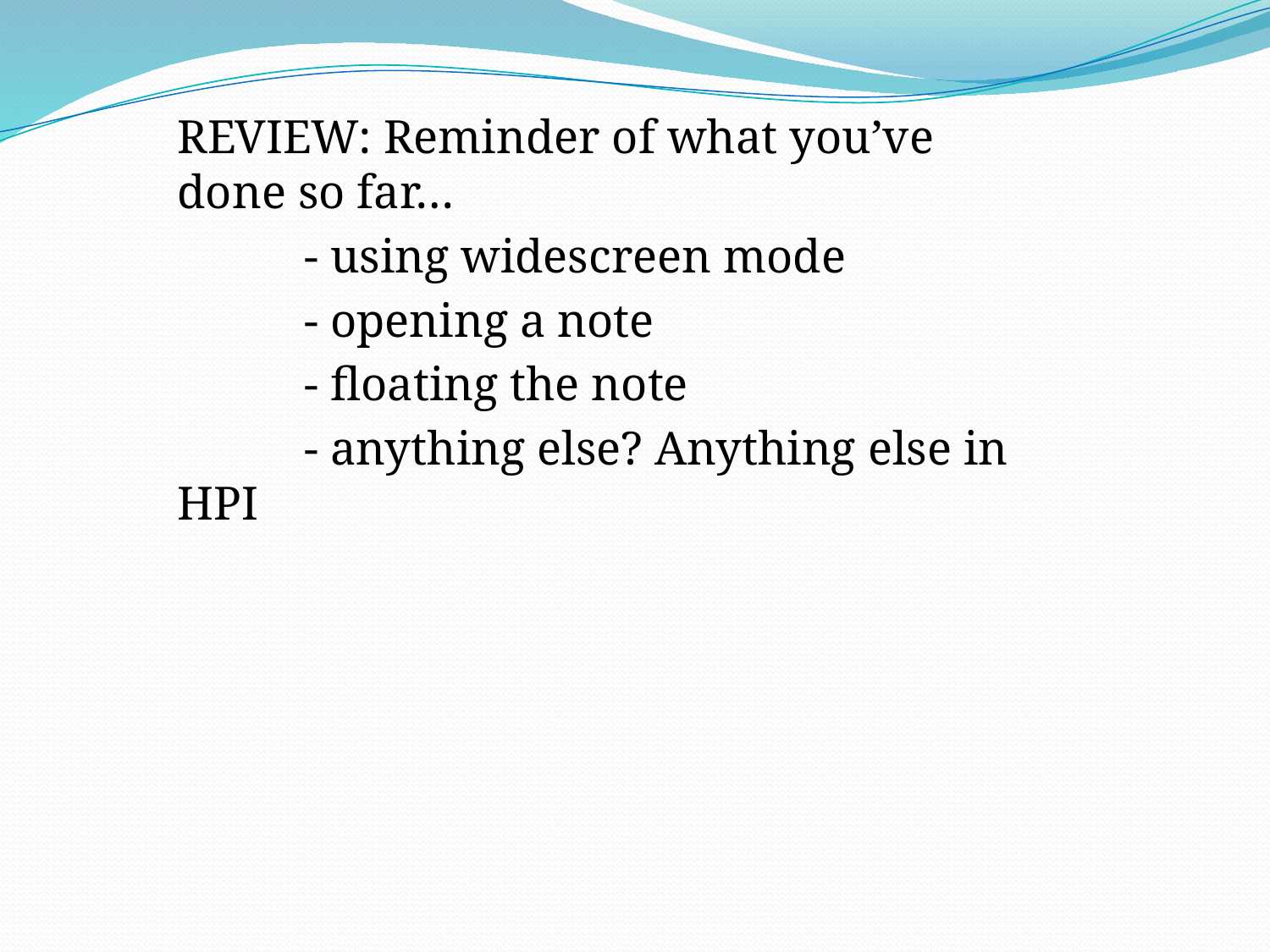

REVIEW: Reminder of what you’ve done so far…
	- using widescreen mode
	- opening a note
	- floating the note
	- anything else? Anything else in HPI

## Slide 4
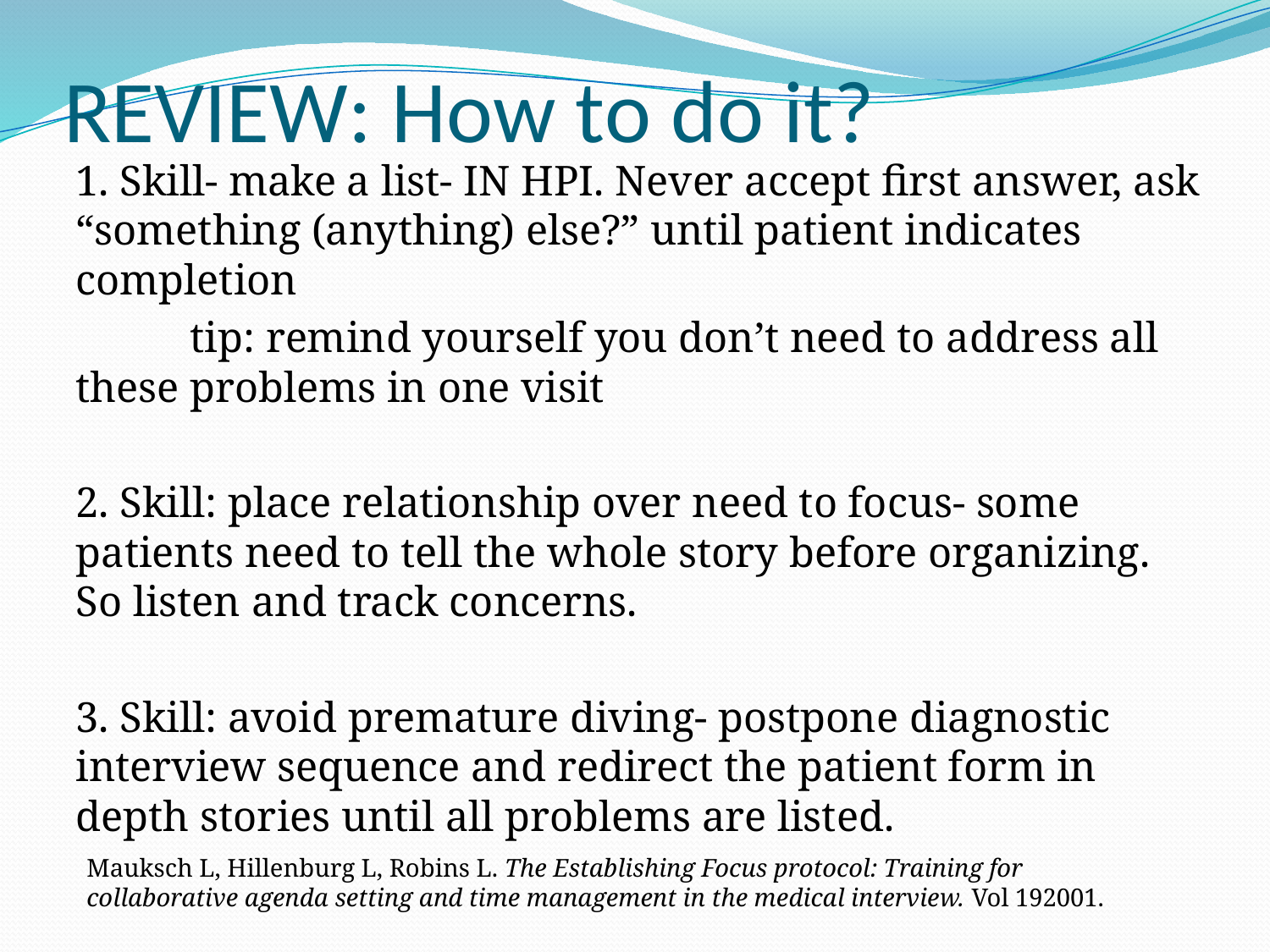

# REVIEW: How to do it?
1. Skill- make a list- IN HPI. Never accept first answer, ask “something (anything) else?” until patient indicates completion
	tip: remind yourself you don’t need to address all these problems in one visit
2. Skill: place relationship over need to focus- some patients need to tell the whole story before organizing. So listen and track concerns.
3. Skill: avoid premature diving- postpone diagnostic interview sequence and redirect the patient form in depth stories until all problems are listed.
Mauksch L, Hillenburg L, Robins L. The Establishing Focus protocol: Training for collaborative agenda setting and time management in the medical interview. Vol 192001.

## Slide 5
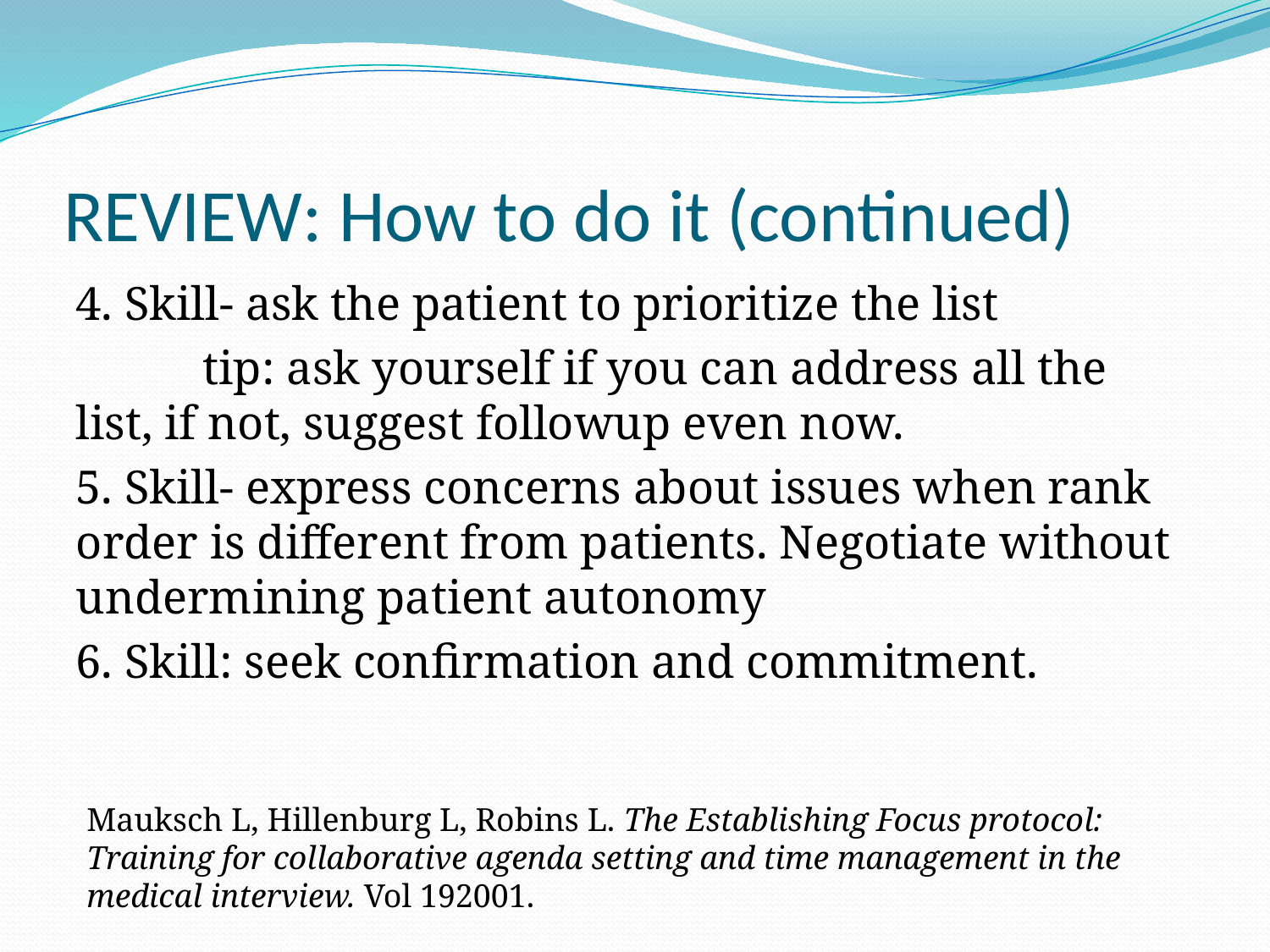

# REVIEW: How to do it (continued)
4. Skill- ask the patient to prioritize the list
	tip: ask yourself if you can address all the list, if not, suggest followup even now.
5. Skill- express concerns about issues when rank order is different from patients. Negotiate without undermining patient autonomy
6. Skill: seek confirmation and commitment.
Mauksch L, Hillenburg L, Robins L. The Establishing Focus protocol: Training for collaborative agenda setting and time management in the medical interview. Vol 192001.

## Slide 6
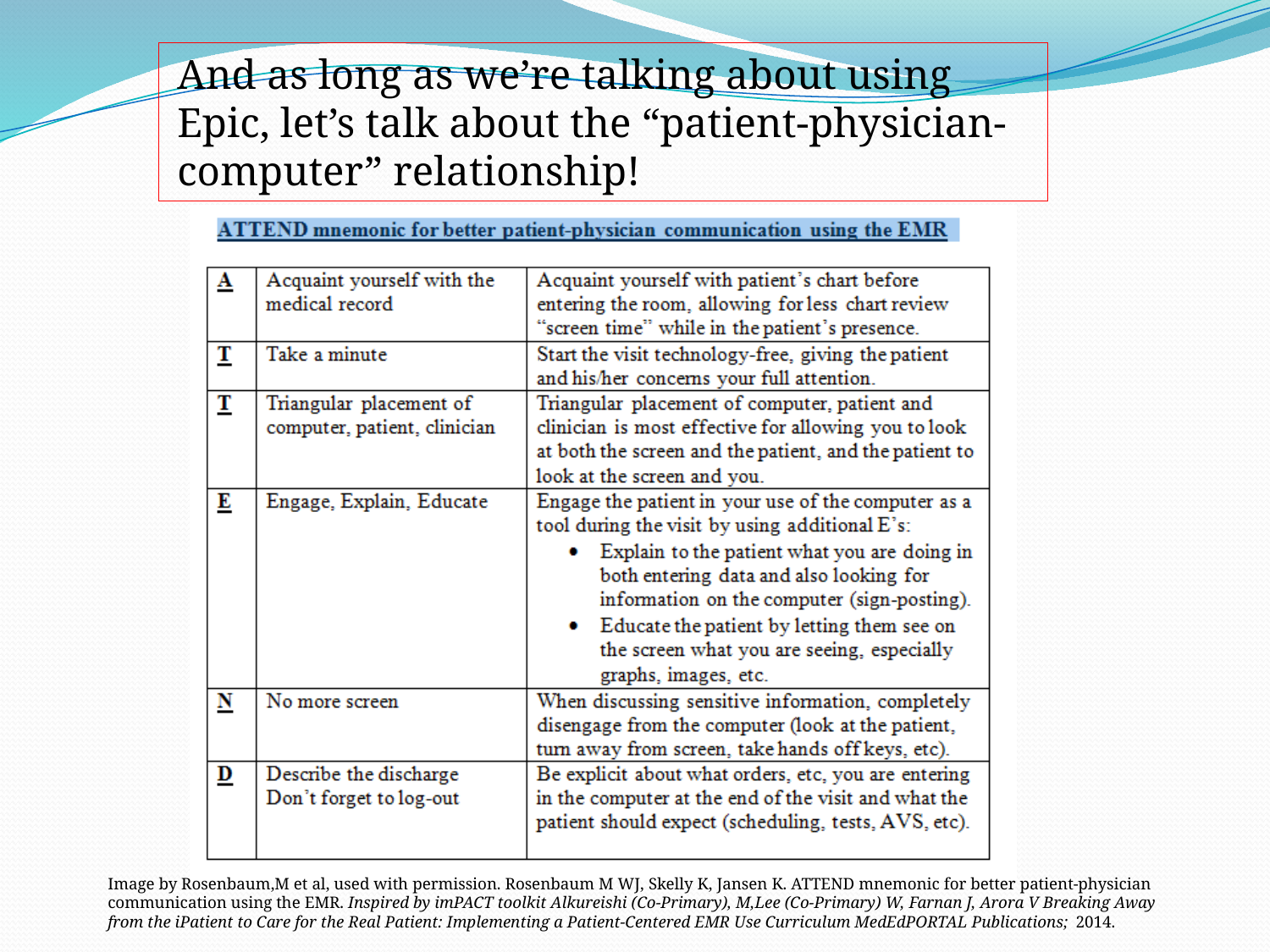

And as long as we’re talking about using Epic, let’s talk about the “patient-physician-computer” relationship!
Image by Rosenbaum,M et al, used with permission. Rosenbaum M WJ, Skelly K, Jansen K. ATTEND mnemonic for better patient-physician communication using the EMR. Inspired by imPACT toolkit Alkureishi (Co-Primary), M,Lee (Co-Primary) W, Farnan J, Arora V Breaking Away from the iPatient to Care for the Real Patient: Implementing a Patient-Centered EMR Use Curriculum MedEdPORTAL Publications; 2014.

## Slide 7
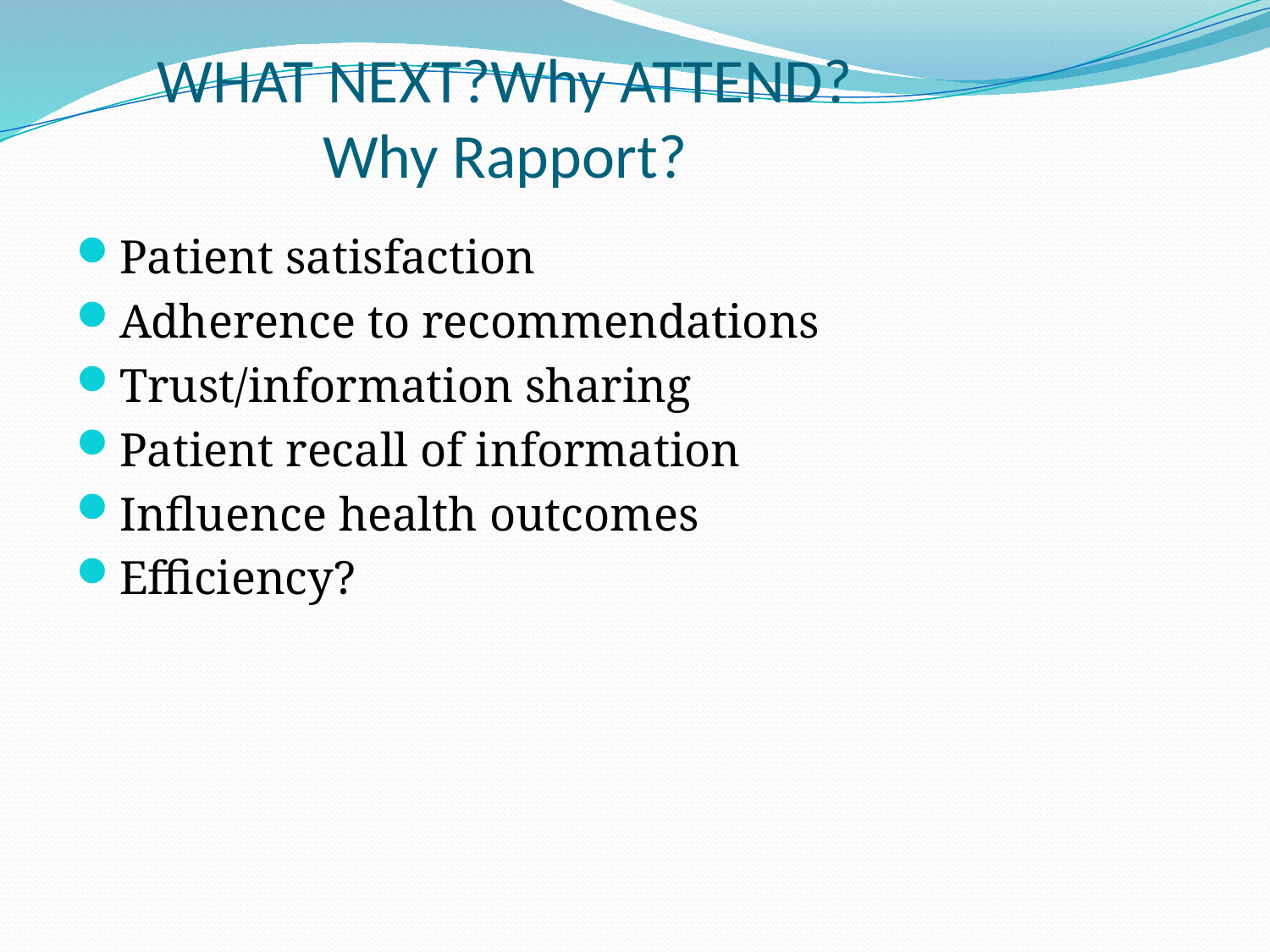

# WHAT NEXT?Why ATTEND? Why Rapport?
Patient satisfaction
Adherence to recommendations
Trust/information sharing
Patient recall of information
Influence health outcomes
Efficiency?

## Slide 8
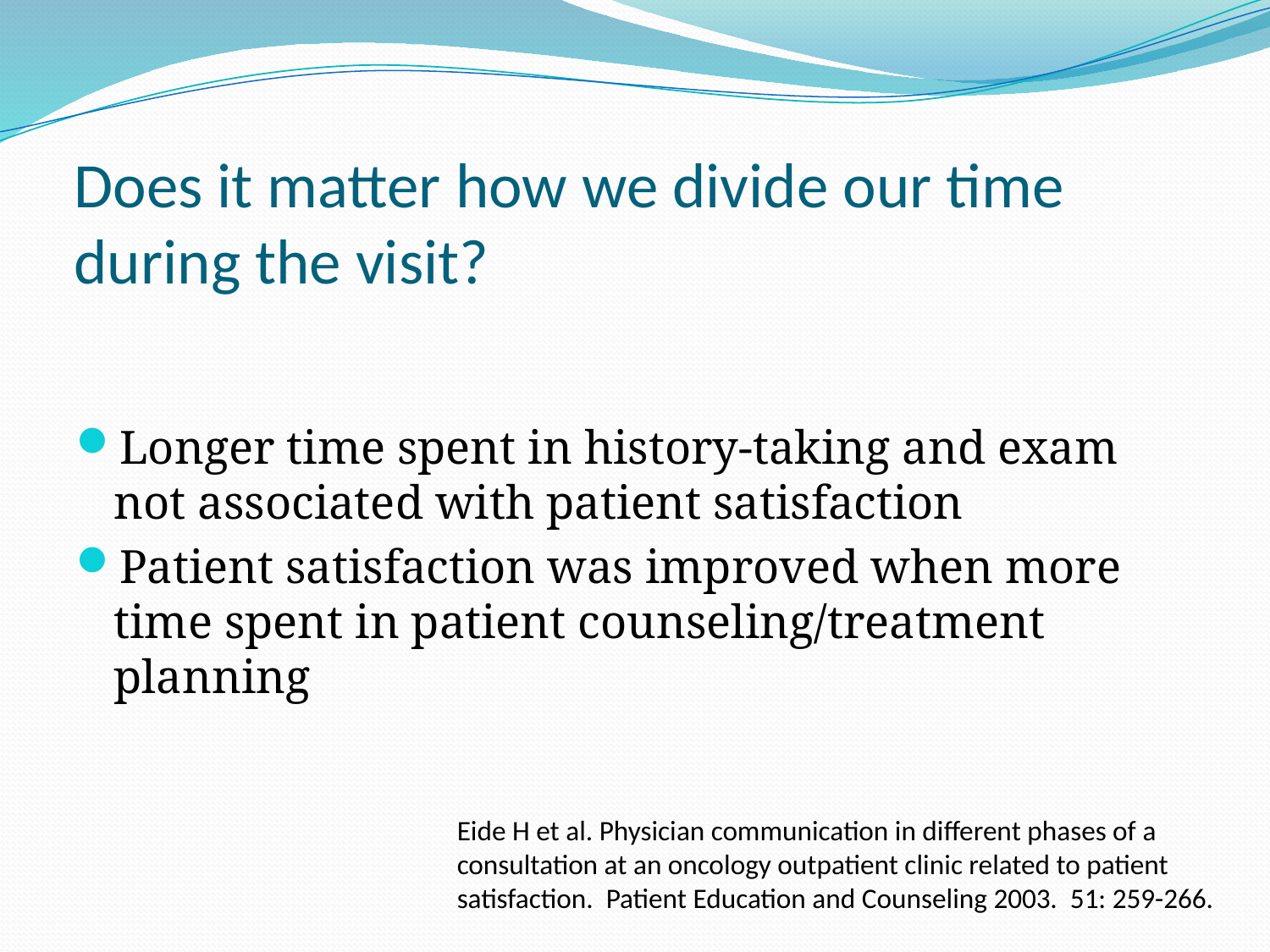

# Does it matter how we divide our time during the visit?
Longer time spent in history-taking and exam not associated with patient satisfaction
Patient satisfaction was improved when more time spent in patient counseling/treatment planning
Eide H et al. Physician communication in different phases of a consultation at an oncology outpatient clinic related to patient satisfaction. Patient Education and Counseling 2003. 51: 259-266.

## Slide 9
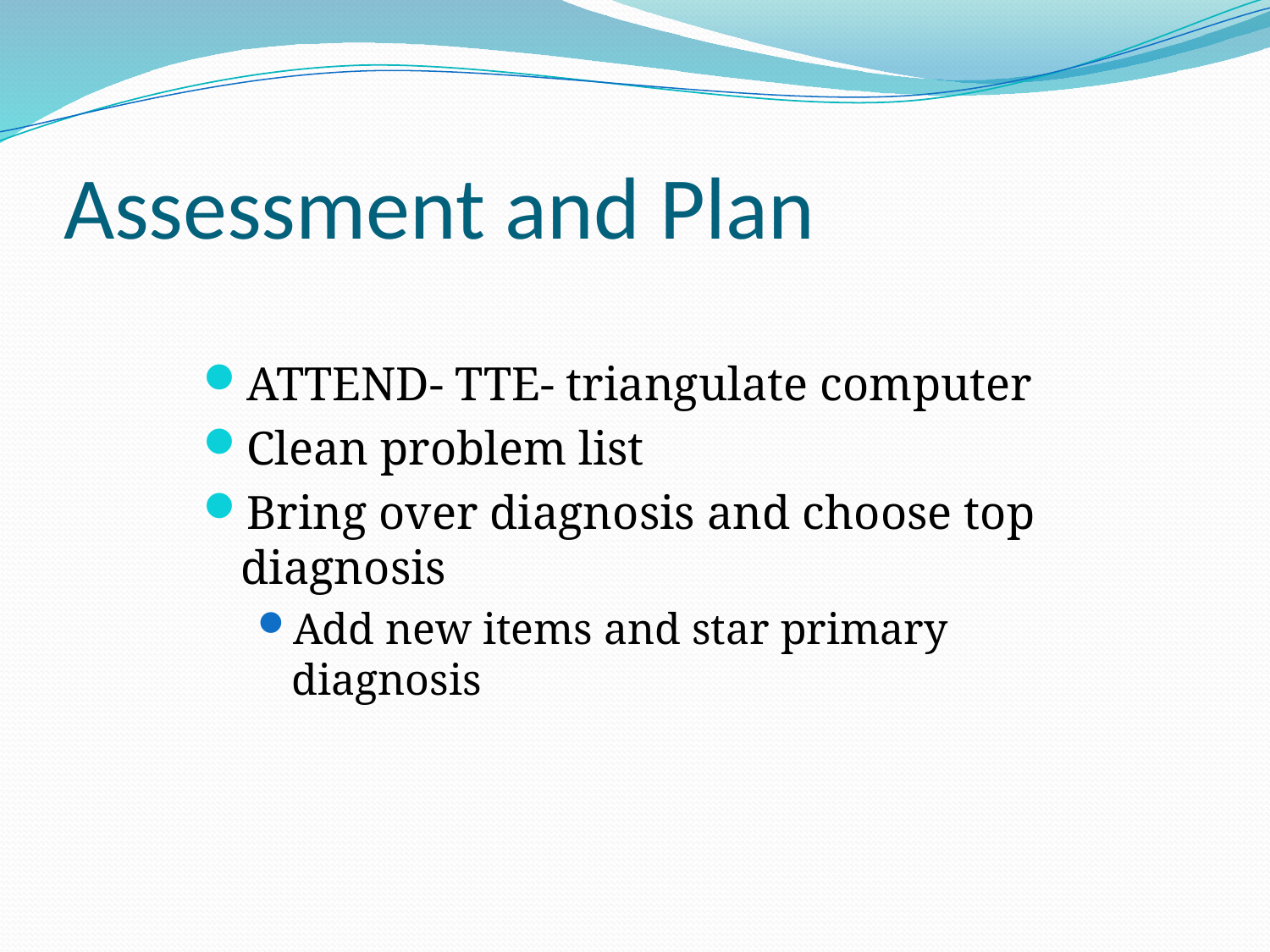

# Assessment and Plan
ATTEND- TTE- triangulate computer
Clean problem list
Bring over diagnosis and choose top diagnosis
Add new items and star primary diagnosis

## Slide 10
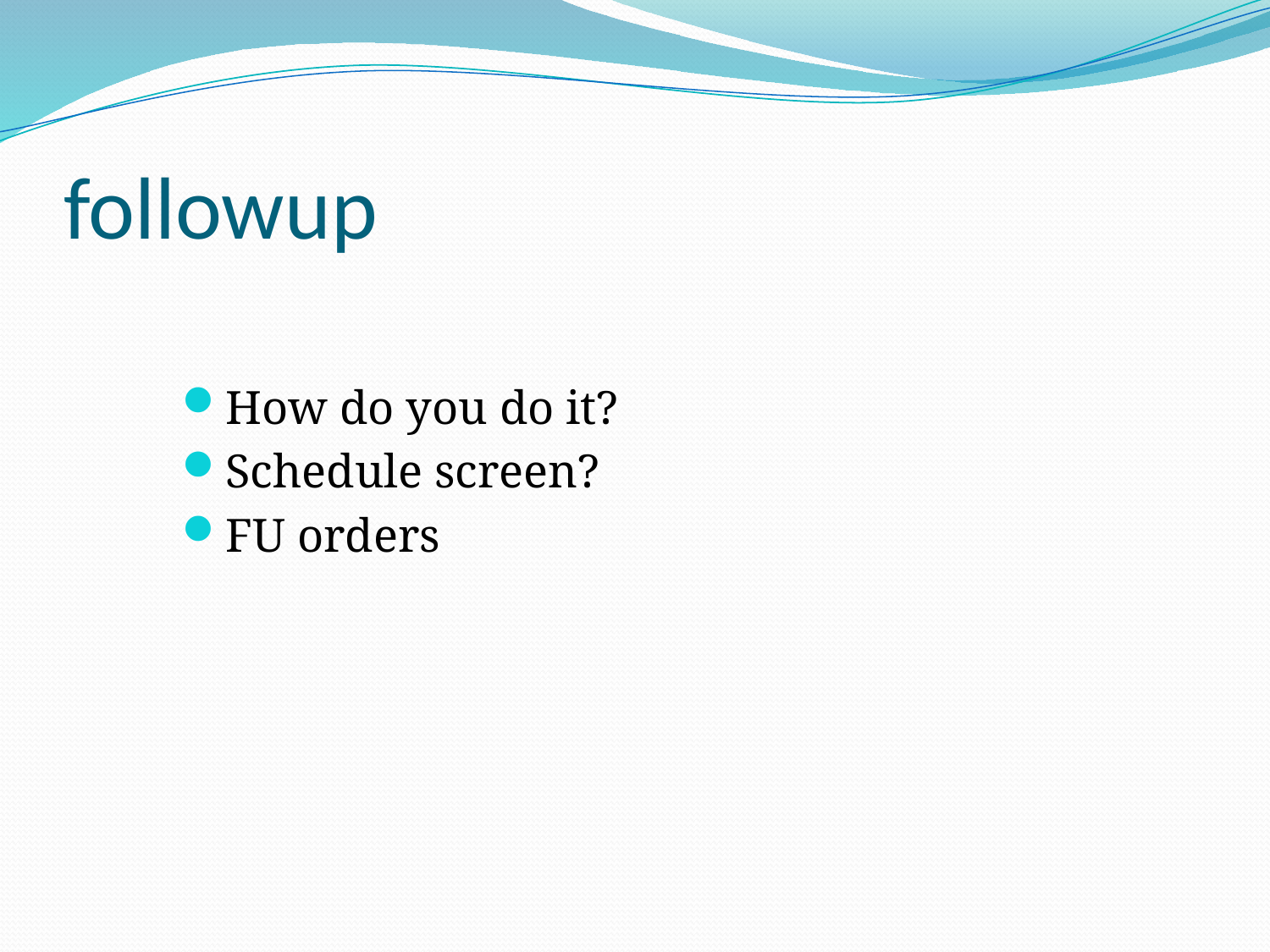

# followup
How do you do it?
Schedule screen?
FU orders

## Slide 11
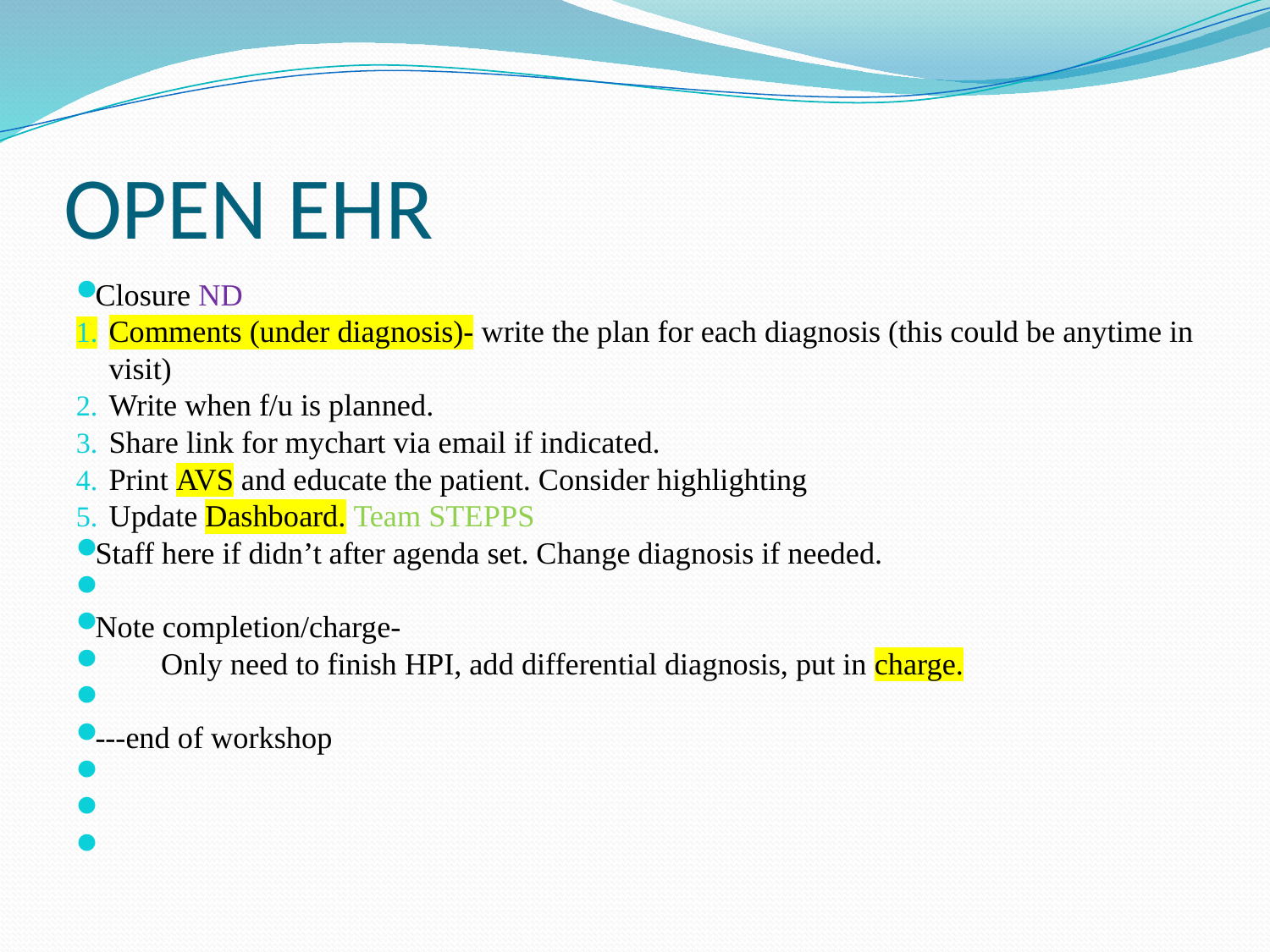

# OPEN EHR
Closure ND
Comments (under diagnosis)- write the plan for each diagnosis (this could be anytime in visit)
Write when f/u is planned.
Share link for mychart via email if indicated.
Print AVS and educate the patient. Consider highlighting
Update Dashboard. Team STEPPS
Staff here if didn’t after agenda set. Change diagnosis if needed.
Note completion/charge-
	Only need to finish HPI, add differential diagnosis, put in charge.
---end of workshop

## Slide 12
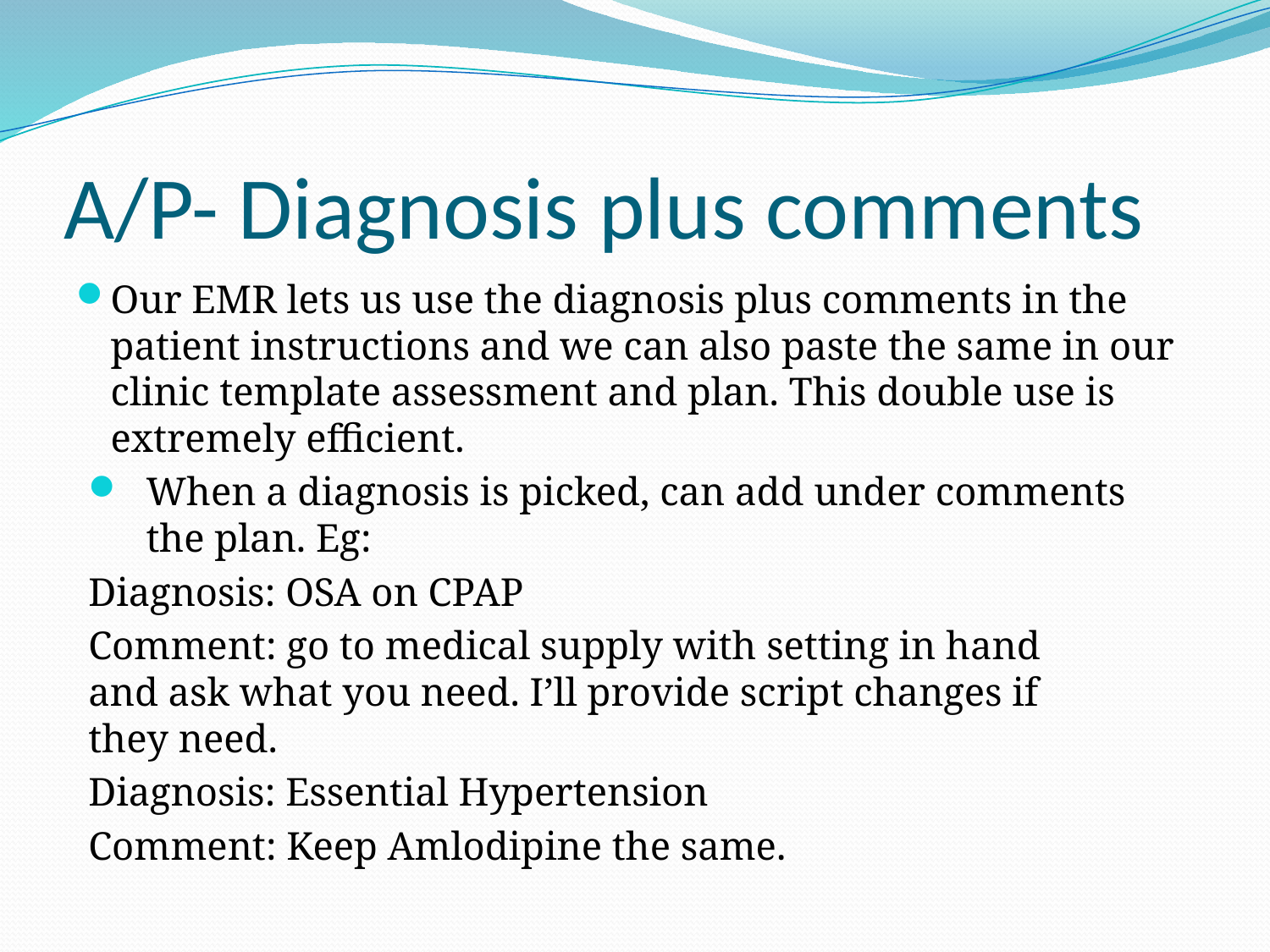

# A/P- Diagnosis plus comments
Our EMR lets us use the diagnosis plus comments in the patient instructions and we can also paste the same in our clinic template assessment and plan. This double use is extremely efficient.
When a diagnosis is picked, can add under comments the plan. Eg:
	Diagnosis: OSA on CPAP
	Comment: go to medical supply with setting in hand 	and ask what you need. I’ll provide script changes if 	they need.
	Diagnosis: Essential Hypertension
	Comment: Keep Amlodipine the same.

## Slide 13
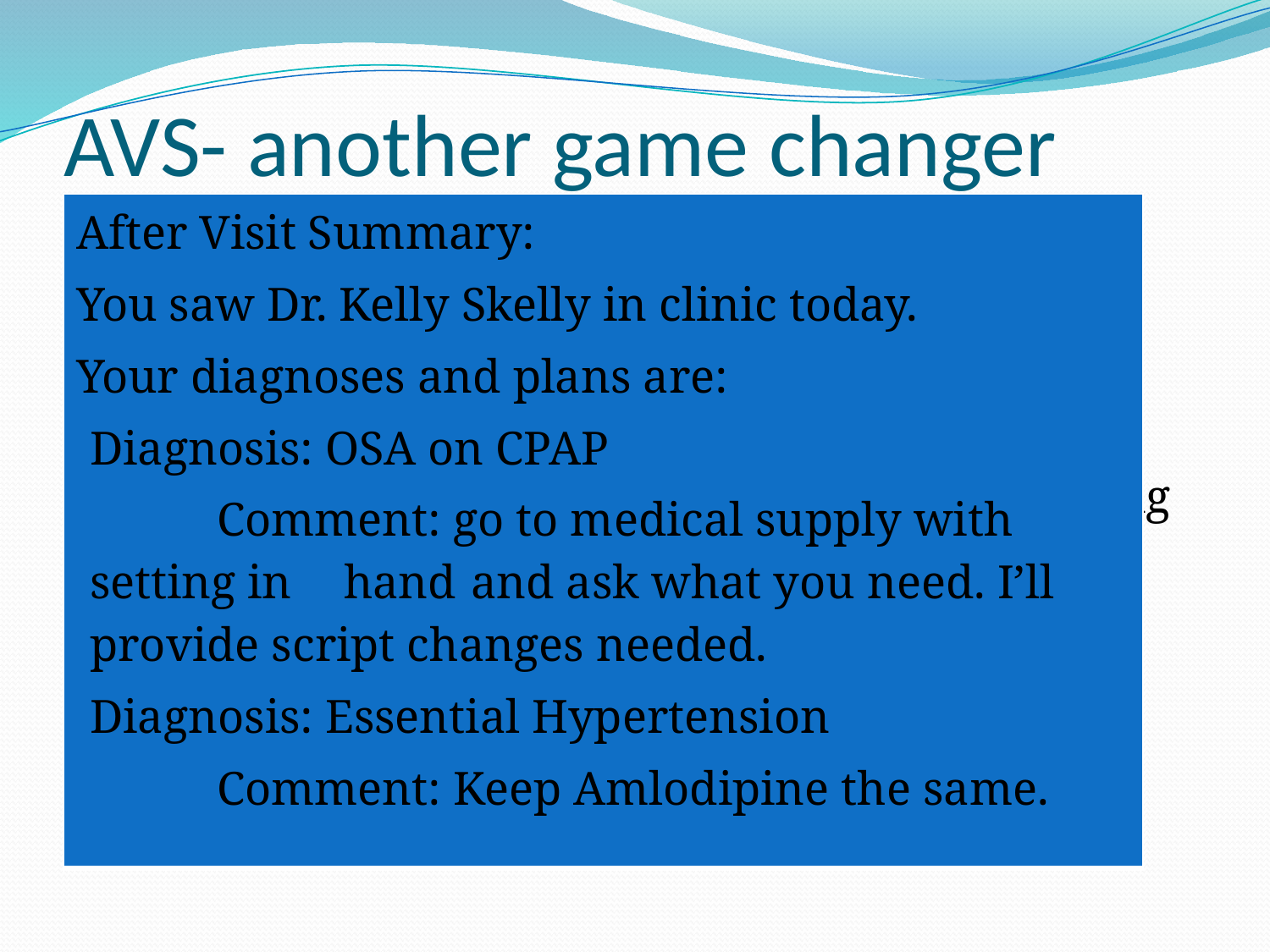

# AVS- another game changer
| After Visit Summary: You saw Dr. Kelly Skelly in clinic today. Your diagnoses and plans are: Diagnosis: OSA on CPAP Comment: go to medical supply with setting in hand and ask what you need. I’ll provide script changes needed. Diagnosis: Essential Hypertension Comment: Keep Amlodipine the same. |
| --- |
You saw Dr. Kelly Skelly in clinic today.
Your diagnoses and plans are:
Diagnosis: OSA on CPAP
	Comment: go to medical supply with setting in 	hand 	and ask what you need. I’ll provide script 	changes if they need.
Diagnosis: Essential Hypertension
	Comment: Keep Amlodipine the same.

## Slide 14
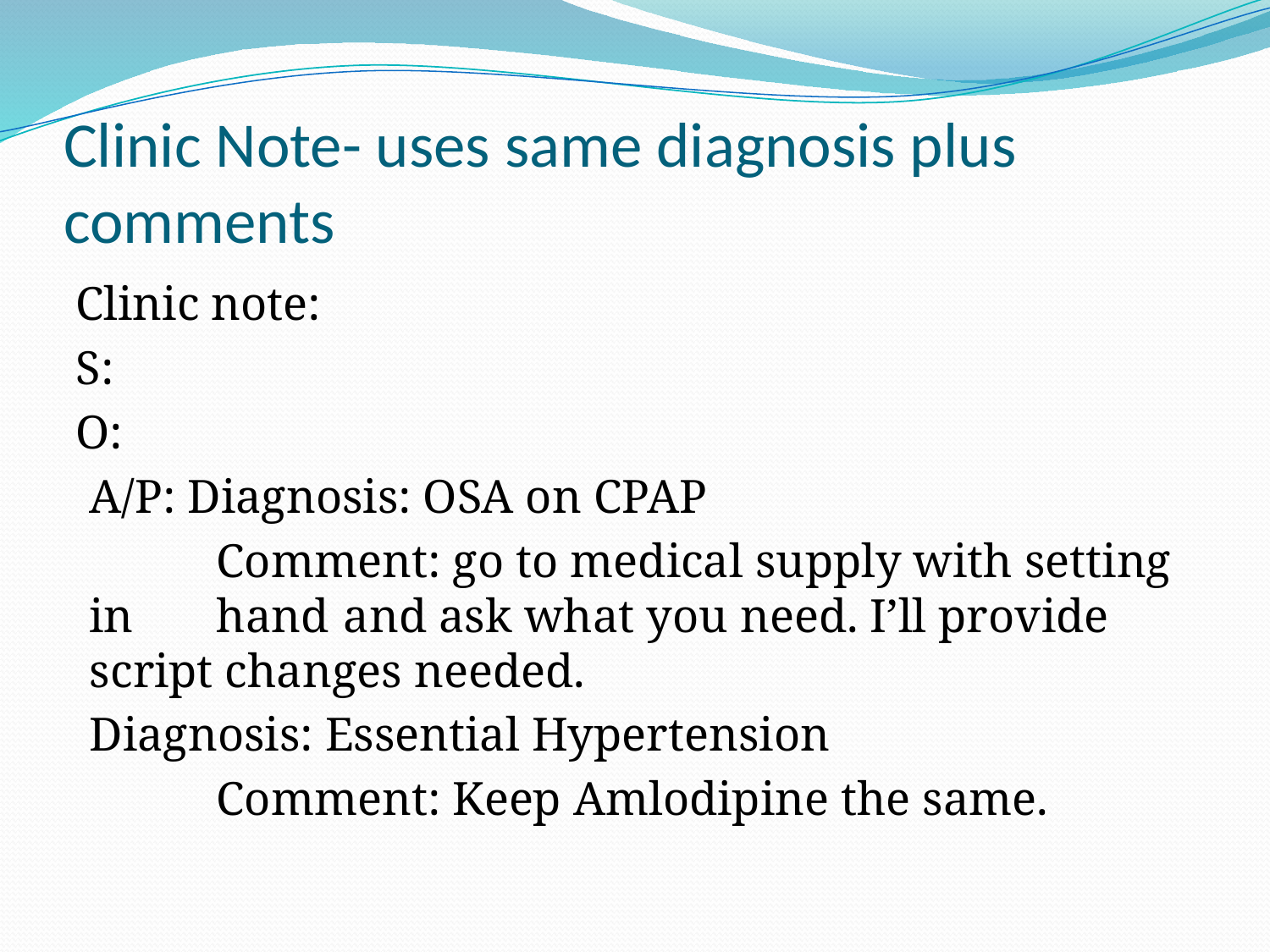

# Clinic Note- uses same diagnosis plus comments
Clinic note:
S:
O:
A/P: Diagnosis: OSA on CPAP
	Comment: go to medical supply with setting in 	hand 	and ask what you need. I’ll provide script changes needed.
Diagnosis: Essential Hypertension
	Comment: Keep Amlodipine the same.

## Slide 15
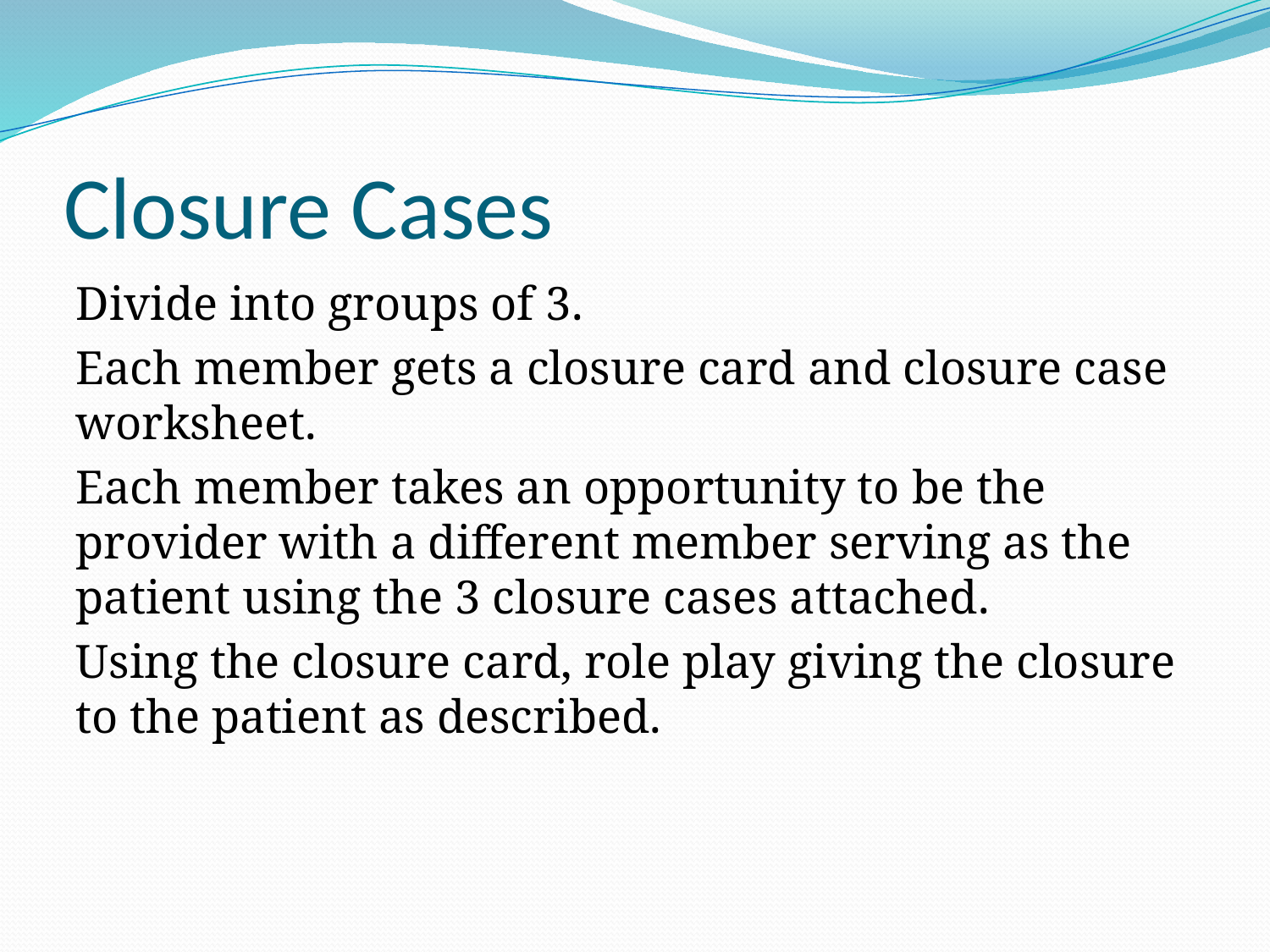

# Closure Cases
Divide into groups of 3.
Each member gets a closure card and closure case worksheet.
Each member takes an opportunity to be the provider with a different member serving as the patient using the 3 closure cases attached.
Using the closure card, role play giving the closure to the patient as described.

## Slide 16
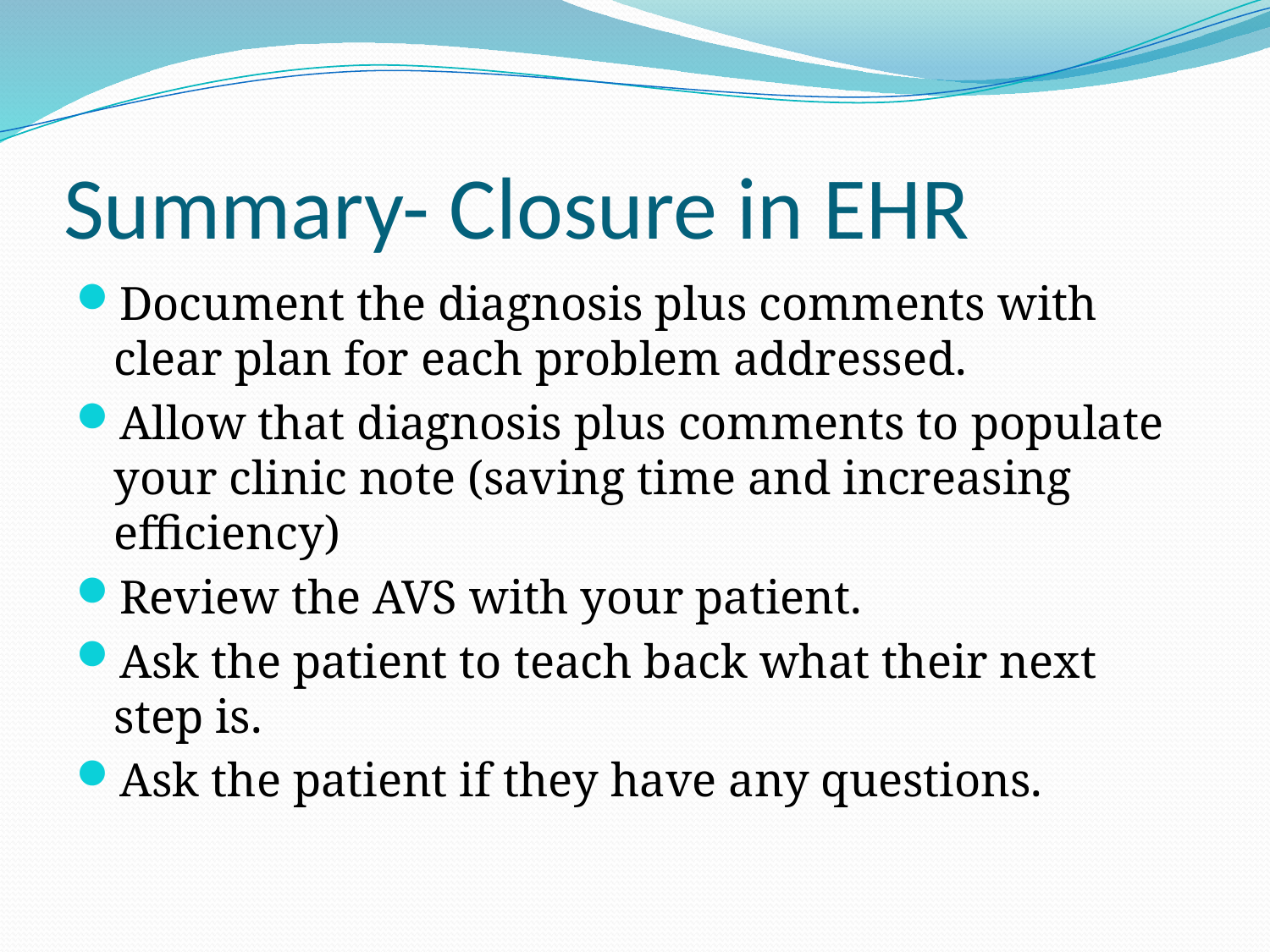

# Summary- Closure in EHR
Document the diagnosis plus comments with clear plan for each problem addressed.
Allow that diagnosis plus comments to populate your clinic note (saving time and increasing efficiency)
Review the AVS with your patient.
Ask the patient to teach back what their next step is.
Ask the patient if they have any questions.

## Slide 17
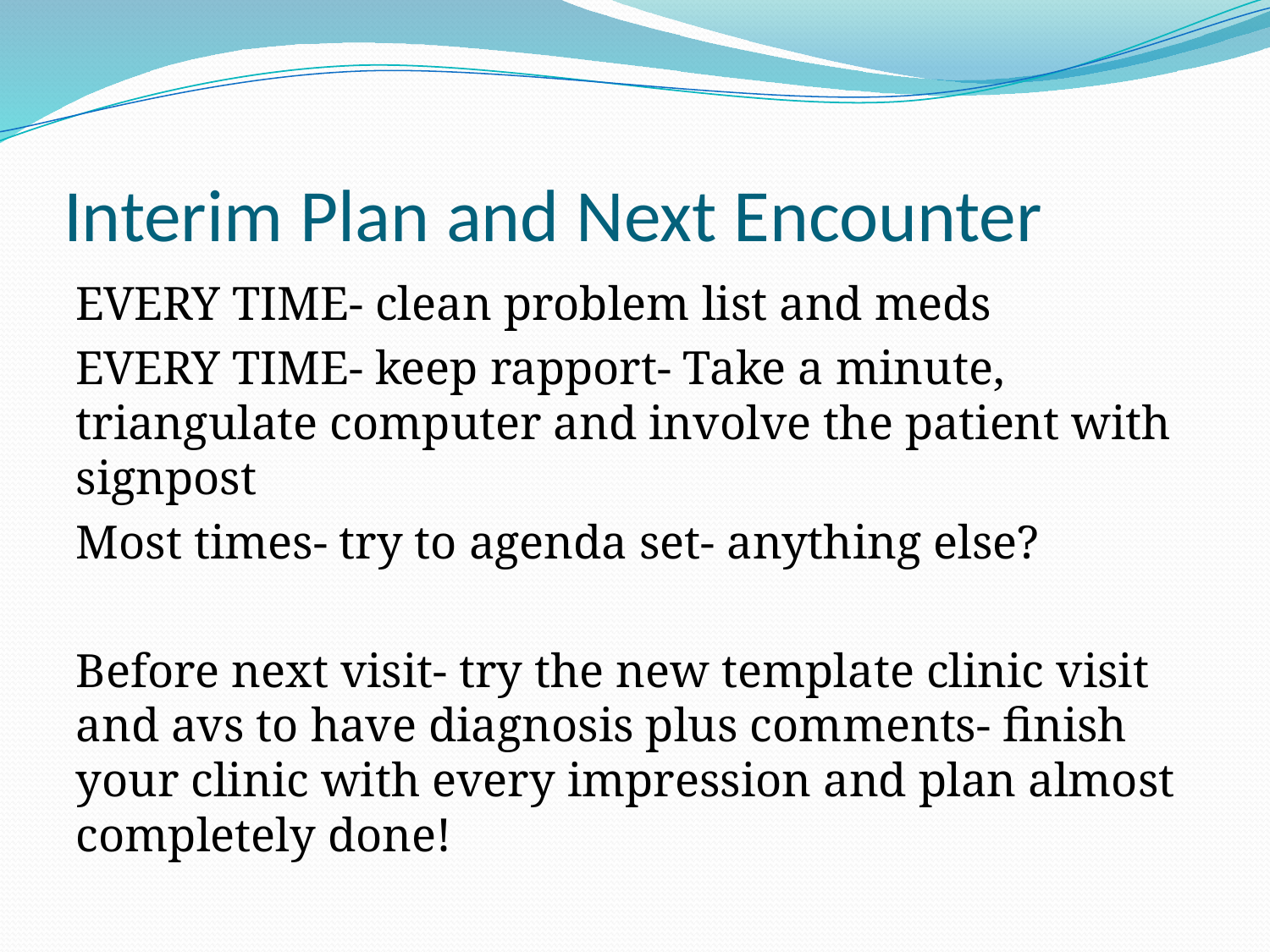

# Interim Plan and Next Encounter
EVERY TIME- clean problem list and meds
EVERY TIME- keep rapport- Take a minute, triangulate computer and involve the patient with signpost
Most times- try to agenda set- anything else?
Before next visit- try the new template clinic visit and avs to have diagnosis plus comments- finish your clinic with every impression and plan almost completely done!

## Slide 18
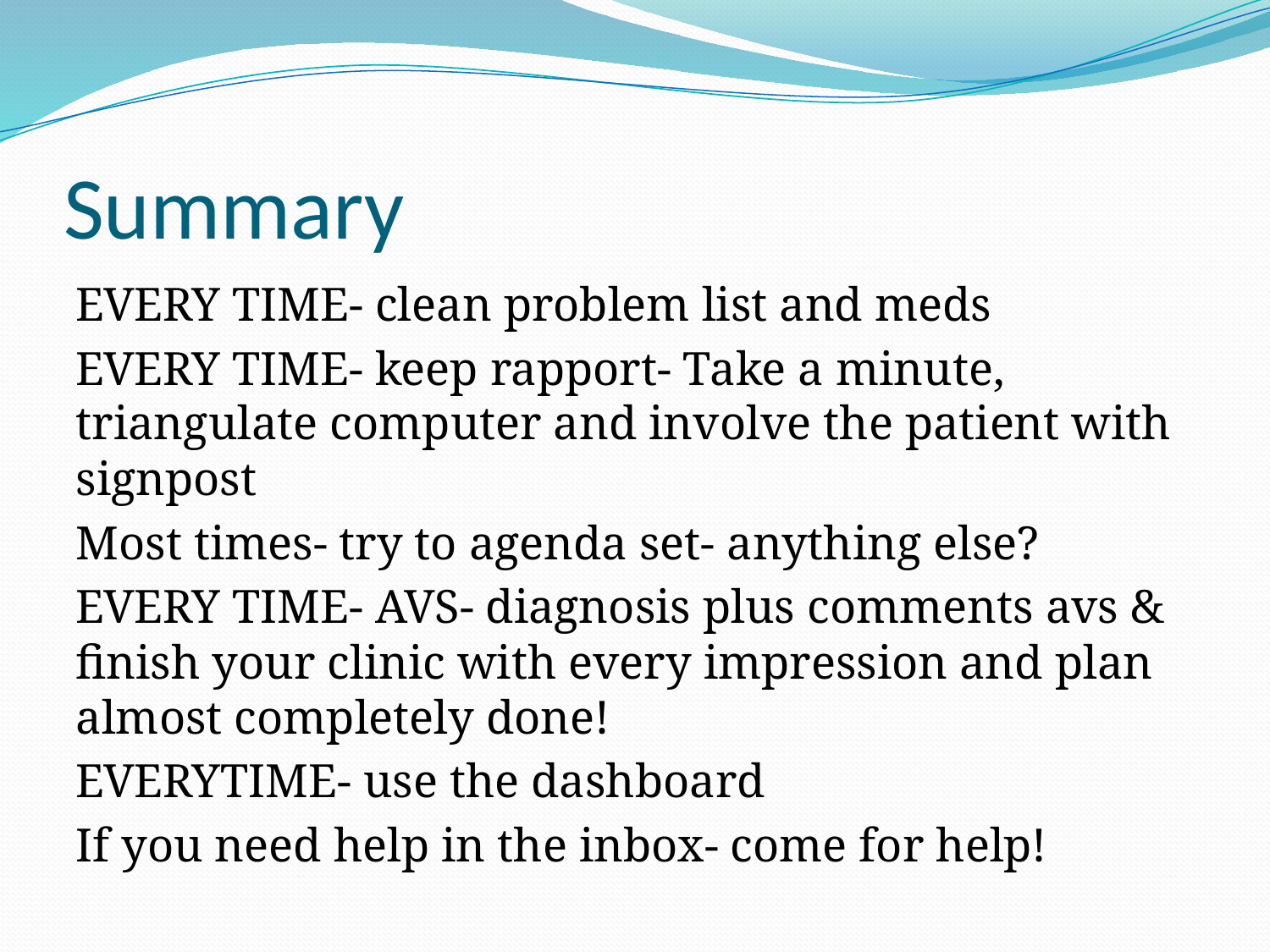

# Summary
EVERY TIME- clean problem list and meds
EVERY TIME- keep rapport- Take a minute, triangulate computer and involve the patient with signpost
Most times- try to agenda set- anything else?
EVERY TIME- AVS- diagnosis plus comments avs & finish your clinic with every impression and plan almost completely done!
EVERYTIME- use the dashboard
If you need help in the inbox- come for help!

## Slide 19
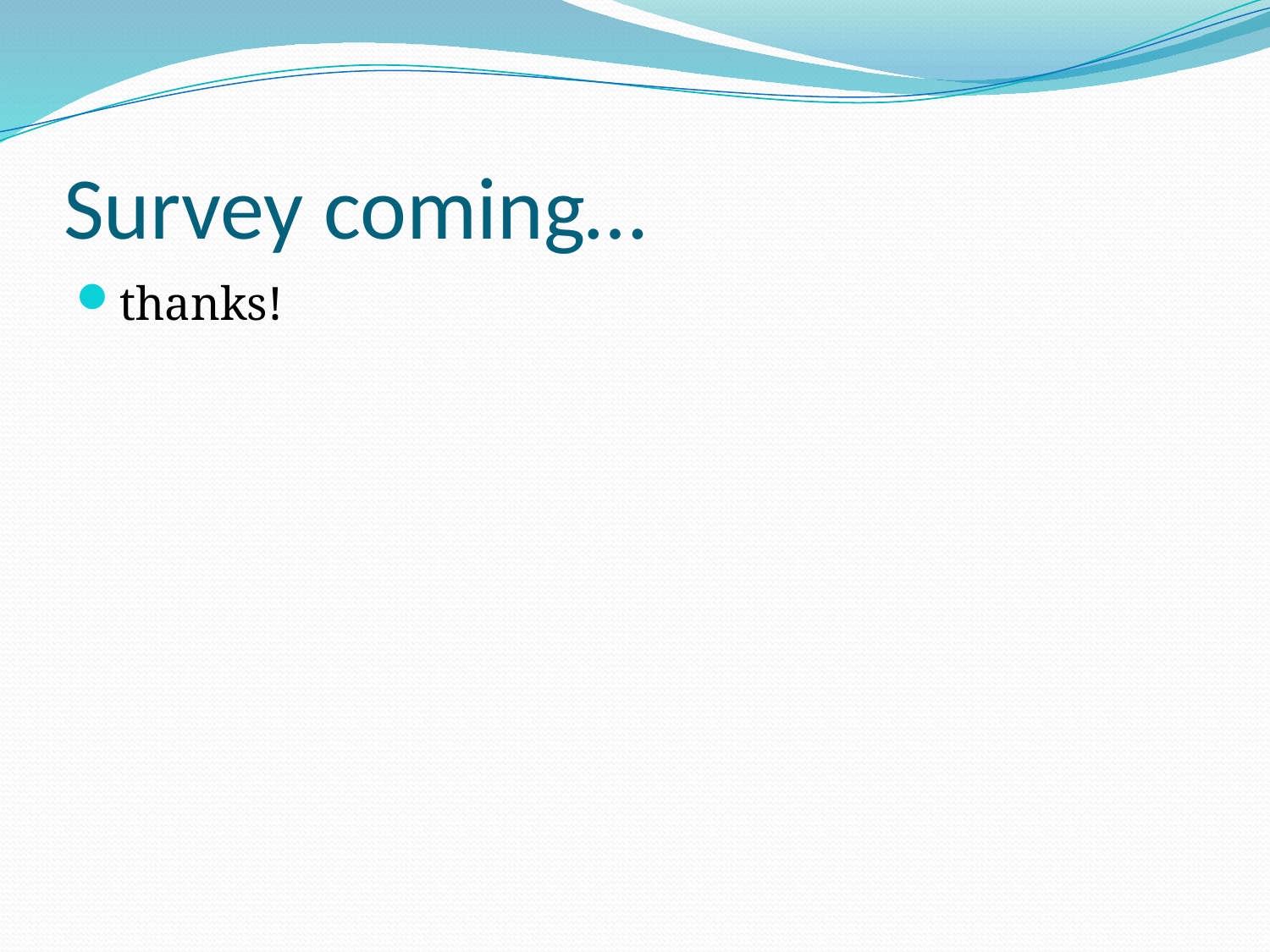

# Survey coming…
thanks!

## Slide 20
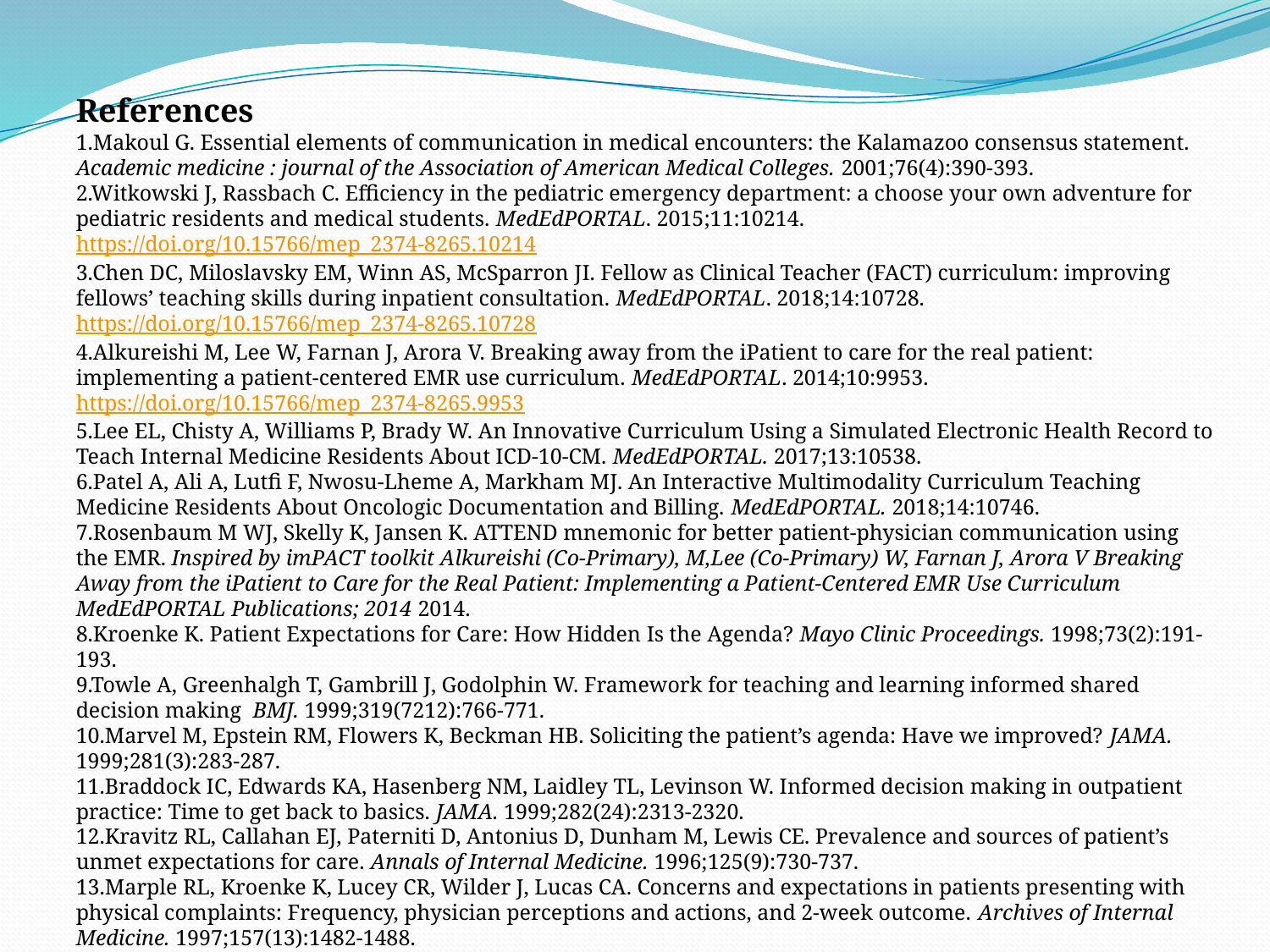

References
1.Makoul G. Essential elements of communication in medical encounters: the Kalamazoo consensus statement. Academic medicine : journal of the Association of American Medical Colleges. 2001;76(4):390-393.
2.Witkowski J, Rassbach C. Efficiency in the pediatric emergency department: a choose your own adventure for pediatric residents and medical students. MedEdPORTAL. 2015;11:10214. https://doi.org/10.15766/mep_2374-8265.10214
3.Chen DC, Miloslavsky EM, Winn AS, McSparron JI. Fellow as Clinical Teacher (FACT) curriculum: improving fellows’ teaching skills during inpatient consultation. MedEdPORTAL. 2018;14:10728. https://doi.org/10.15766/mep_2374-8265.10728
4.Alkureishi M, Lee W, Farnan J, Arora V. Breaking away from the iPatient to care for the real patient: implementing a patient-centered EMR use curriculum. MedEdPORTAL. 2014;10:9953. https://doi.org/10.15766/mep_2374-8265.9953
5.Lee EL, Chisty A, Williams P, Brady W. An Innovative Curriculum Using a Simulated Electronic Health Record to Teach Internal Medicine Residents About ICD-10-CM. MedEdPORTAL. 2017;13:10538.
6.Patel A, Ali A, Lutfi F, Nwosu-Lheme A, Markham MJ. An Interactive Multimodality Curriculum Teaching Medicine Residents About Oncologic Documentation and Billing. MedEdPORTAL. 2018;14:10746.
7.Rosenbaum M WJ, Skelly K, Jansen K. ATTEND mnemonic for better patient-physician communication using the EMR. Inspired by imPACT toolkit Alkureishi (Co-Primary), M,Lee (Co-Primary) W, Farnan J, Arora V Breaking Away from the iPatient to Care for the Real Patient: Implementing a Patient-Centered EMR Use Curriculum MedEdPORTAL Publications; 2014 2014.
8.Kroenke K. Patient Expectations for Care: How Hidden Is the Agenda? Mayo Clinic Proceedings. 1998;73(2):191-193.
9.Towle A, Greenhalgh T, Gambrill J, Godolphin W. Framework for teaching and learning informed shared decision making BMJ. 1999;319(7212):766-771.
10.Marvel M, Epstein RM, Flowers K, Beckman HB. Soliciting the patient’s agenda: Have we improved? JAMA. 1999;281(3):283-287.
11.Braddock IC, Edwards KA, Hasenberg NM, Laidley TL, Levinson W. Informed decision making in outpatient practice: Time to get back to basics. JAMA. 1999;282(24):2313-2320.
12.Kravitz RL, Callahan EJ, Paterniti D, Antonius D, Dunham M, Lewis CE. Prevalence and sources of patient’s unmet expectations for care. Annals of Internal Medicine. 1996;125(9):730-737.
13.Marple RL, Kroenke K, Lucey CR, Wilder J, Lucas CA. Concerns and expectations in patients presenting with physical complaints: Frequency, physician perceptions and actions, and 2-week outcome. Archives of Internal Medicine. 1997;157(13):1482-1488.
14.Mauksch L, Hillenburg L, Robins L. The Establishing Focus protocol: Training for collaborative agenda setting and time management in the medical interview. Vol 192001.
15.Østbye T, Yarnall KSH, Krause KM, Pollak KI, Gradison M, Michener JL. Is There Time for Management of Patients With Chronic Diseases in Primary Care? The Annals of Family Medicine. 2005;3(3):209-214.

## Slide 21
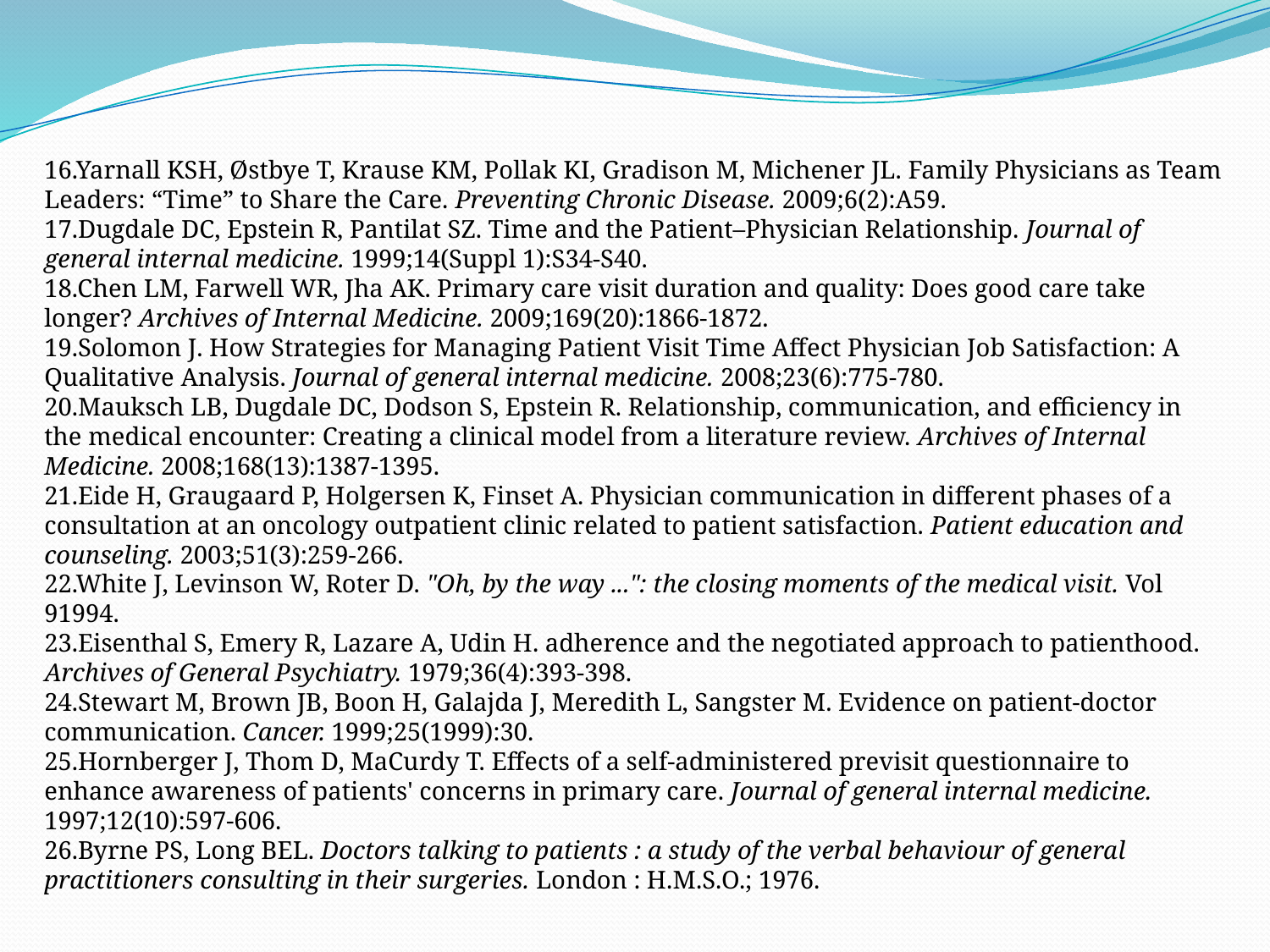

16.Yarnall KSH, Østbye T, Krause KM, Pollak KI, Gradison M, Michener JL. Family Physicians as Team Leaders: “Time” to Share the Care. Preventing Chronic Disease. 2009;6(2):A59.
17.Dugdale DC, Epstein R, Pantilat SZ. Time and the Patient–Physician Relationship. Journal of general internal medicine. 1999;14(Suppl 1):S34-S40.
18.Chen LM, Farwell WR, Jha AK. Primary care visit duration and quality: Does good care take longer? Archives of Internal Medicine. 2009;169(20):1866-1872.
19.Solomon J. How Strategies for Managing Patient Visit Time Affect Physician Job Satisfaction: A Qualitative Analysis. Journal of general internal medicine. 2008;23(6):775-780.
20.Mauksch LB, Dugdale DC, Dodson S, Epstein R. Relationship, communication, and efficiency in the medical encounter: Creating a clinical model from a literature review. Archives of Internal Medicine. 2008;168(13):1387-1395.
21.Eide H, Graugaard P, Holgersen K, Finset A. Physician communication in different phases of a consultation at an oncology outpatient clinic related to patient satisfaction. Patient education and counseling. 2003;51(3):259-266.
22.White J, Levinson W, Roter D. "Oh, by the way ...": the closing moments of the medical visit. Vol 91994.
23.Eisenthal S, Emery R, Lazare A, Udin H. adherence and the negotiated approach to patienthood. Archives of General Psychiatry. 1979;36(4):393-398.
24.Stewart M, Brown JB, Boon H, Galajda J, Meredith L, Sangster M. Evidence on patient-doctor communication. Cancer. 1999;25(1999):30.
25.Hornberger J, Thom D, MaCurdy T. Effects of a self-administered previsit questionnaire to enhance awareness of patients' concerns in primary care. Journal of general internal medicine. 1997;12(10):597-606.
26.Byrne PS, Long BEL. Doctors talking to patients : a study of the verbal behaviour of general practitioners consulting in their surgeries. London : H.M.S.O.; 1976.
