# Supplementary material for: A Curriculum for Teaching Clinical Efficiency Focusing on Specific Communication Skills While Maximizing the Electronic Health Record
Source: MedEdPORTAL. 2020 Oct 29;16:10989. doi: 10.15766/mep_2374-8265.10989 (PMC7597939; doi:10.15766/mep_2374-8265.10989)
Supplement: Supplementary file 1 — Efficiency Preworkshop Needs Assessment Survey.docxWorkshop 1 - Setting up the Template and Working in EHR.pptxSample Clinic Note and AVS Template.docxWorkshop 2 - Preclinic Preparation and Rapport Building.pptxEfficiency ATTEND Practice Card.docxWorkshop 3 - Agenda Setting and Relationship Maintenance.pptxEfficiency Agenda Setting Practice.docxWorkshop 4 - Visit Closure.pptxEfficiency Closure Card and Cases.docxEfficiency Postworkshop Evaluation.docx [file mep_2374-8265.10989-s001.zip › I. Efficiency Closure Card and Cases.docx]

Appendix I Closure Card and Cases

**Signal Closure**

- - In summary….
  - So to go over what we talked about…
  - Based on our encounter today…
  - Before the next time…
  - The next steps are…

**Checklist for Closing a Visit**

- Signal closure
- Summarize the visit
- Review the Plan
- Check understanding
- Next visit and interim contact
- Demonstrate caring and offer reassurance
- Logistics and immediate next steps

**Closure Cases:**

Doctor 1: Your patient is a 52 yo woman who presented for an annual physical exam. You discussed breast and colon cancer screening and have negotiated to do a mammogram and a colonoscopy. She is also overweight and you both agreed that she could exercise one more time per week. You ordered a screening lipid panel as well.

Patient 1: You are a 52 yo woman who presented for an annual physical exam. You discussed breast and colon cancer screening with your doctor and are agreeable to having a mammogram but still have questions about the colonoscopy prep. You are going to try to exercise one more time pre week to work on your weight. You know the doctor said something about a lab, but don’t remember what it was or where the lab is located. You also want to know when you will get your lab results and the results of your other tests.

Doctor 2: Your patient is a 35 yo woman who is well known to you. She is following up for menorrhagia which was causing mild anemia and is much improved after placement of a mirena IUD. She is married and does not desire children now, but may decide otherwise. She is healthy and is taking iron supplements for her anemia. You would like her to have a repeat hemoglobin tested in 6 weeks.

Patient 2: You are a 35 yo woman who is following up for menorrhagia and anemia. Your menorrhagia is improved after you and your doctor decided on Mirena IUD placement. You are taking iron, but don’t like it because it is causing constipation, but you haven’t brought this up with your doctor yet. You are also wondering what you should do if you and your husband decide to have another child.

Doctor 3: Your patient is a 60 yo man with hypertension. His blood pressure is uncontrolled, so in addition to his chlorthalidone, you have decided together to start another medication, and you sent a prescription to his pharmacy using e-prescribing. You would also like to check his potassium in the next 7-14 days and will order labs to be drawn without a provider encounter.

Patient 3: You are a 60 yo man with hypertension which has not been at goal, and you and your doctor decided to start a new medication. You are wondering where the prescription is because you are used to having a paper to take to the pharmacy. You are also wondering when you will get your lab slip and how to call your doctor if you have a problem with the new medication.
