# Supplementary material for: A Curriculum for Teaching Clinical Efficiency Focusing on Specific Communication Skills While Maximizing the Electronic Health Record
Source: MedEdPORTAL. 2020 Oct 29;16:10989. doi: 10.15766/mep_2374-8265.10989 (PMC7597939; doi:10.15766/mep_2374-8265.10989)
Supplement: Supplementary file 1 — Efficiency Preworkshop Needs Assessment Survey.docxWorkshop 1 - Setting up the Template and Working in EHR.pptxSample Clinic Note and AVS Template.docxWorkshop 2 - Preclinic Preparation and Rapport Building.pptxEfficiency ATTEND Practice Card.docxWorkshop 3 - Agenda Setting and Relationship Maintenance.pptxEfficiency Agenda Setting Practice.docxWorkshop 4 - Visit Closure.pptxEfficiency Closure Card and Cases.docxEfficiency Postworkshop Evaluation.docx [file mep_2374-8265.10989-s001.zip › J. Efficiency Postworkshop Evaluation.docx]

| Rate: 1 = Not at all; 2 = Slightly; 3 = Somewhat; 4 = Very; 5 = Extremely | Your Score |
| --- | --- |
| Value/usefulness of this session to you |  |
| Your level of interest in this topic |  |
| Quality of presentation |  |
| How likely you are to apply what you learned today |  |
| Your OVERALL rating for this session |  |

**Pre and Post Test Questions**

(1: don’t agree, 5: strongly agree)

**Pre-clinic preparation**

 -- I feel prepared for every clinic session

 1 2 3 4 5

-- I have my own agenda established for each patient visit prior to the visit

 1 2 3 4 5

-- I have reviewed and updated the problem list prior to every patient visit

1 2 3 4 5

**Rapport building**

--I have good understanding regarding how to build rapport and maintain relationships with patients during clinic visits

1 2 3 4 5

--I am comfortable in practicing behaviors to build rapport and maintain relationships with patients during clinic visits

1 2 3 4 5

-- I am concerned that I unintentionally use nonverbal cues in clinical encounters that may be misinterpreted by my patients.

1 2 3 4 5

**Opening discuss and information gathering**

--I often feel frustrated when trying to get all patient concerns during a patient visit.

1 2 3 4 5

--I always try to allow my patient to complete his/her opening statement

1 2 3 4 5

**Collaborative agenda setting and reaching agreement**

--I am confident about setting an agenda with the patient

1 2 3 4 5

--I have a consistent pattern I use to set the agenda with the patient.

1 2 3 4 5

--I always make sure that the patient and I are agree about our plan through the visit

1 2 3 4 5

**Closure**

--I am frustrated when my patients bring up concerns at the end of a visit.

1 2 3 4 5

--I feel rushed at the end of visits with patients.

1 2 3 4 5

--I have a consistent pattern I use when closing a visit with a patient.

1 2 3 4 5

**Overall,**

--This Workshop will be helpful in improving my clinic efficiency

1 2 3 4 5

--I will likely to continue using some of the strategies in my future practice

1 2 3 4 5

--Do you think we should continue to provide this workshop in the future?

1 2 3 4 5

**AFTER SURVEY (completed in Qualtrics)**

Do you think you are organized other aspects in your life? Yes/no

Do you make lists yes/no

Do you agenda set in clinic? Yes/no if so, What percentage of the clinic visits do you think you agenda set?- Use bar for percentage during 2 week period

Does it help to agenda set? Yes/no

How?

Did agenda setting slow down your visit? Yes/no

How?

What concerns do you have about agenda setting-(**after linked to before**)-did they change?

Did the intervention to promote agenda setting help? Yes/no How?

**One month later**

Do you think you are organized other aspects in your life? Yes/no

Do you make lists yes/no

Do you agenda set in clinic? Yes/no if so, What percentage of the clinic visits do you think you agenda set?- Use bar for percentage at this time in a two weeks of clinic

Does it help to agenda set? Yes/no

How?

Did agenda setting slow down your visit? Yes/no

How?

What concerns do you have about agenda setting-(**after linked to before**)-did they change?

Did the intervention to promote agenda setting help? Yes/no, How?
