# Supplementary material for: A Curriculum for Teaching Clinical Efficiency Focusing on Specific Communication Skills While Maximizing the Electronic Health Record
Source: MedEdPORTAL. 2020 Oct 29;16:10989. doi: 10.15766/mep_2374-8265.10989 (PMC7597939; doi:10.15766/mep_2374-8265.10989)
Supplement: Supplementary file 1 — Efficiency Preworkshop Needs Assessment Survey.docxWorkshop 1 - Setting up the Template and Working in EHR.pptxSample Clinic Note and AVS Template.docxWorkshop 2 - Preclinic Preparation and Rapport Building.pptxEfficiency ATTEND Practice Card.docxWorkshop 3 - Agenda Setting and Relationship Maintenance.pptxEfficiency Agenda Setting Practice.docxWorkshop 4 - Visit Closure.pptxEfficiency Closure Card and Cases.docxEfficiency Postworkshop Evaluation.docx [file mep_2374-8265.10989-s001.zip › _Educational_Summary_Report_10989.pdf]

MedEdPORTAL® The AAMC Journal of Teaching and Learning Resources

Original Publication Open Access

A Curriculum for Teaching Clinical Efficiency Focusing on Specific Communication Skills While Maximizing the Electronic Health Record

Kelly Skelly, MD*, Wendy Shen, MD, Jason Wilbur, MD, Kate Thoma, MD, Jill Endres, MD, Alison Lynch, MD, Anne Gaglioti, MD, Marcy Rosenbaum, PhD

*Corresponding author: kelly-skelly@uiowa.edu

Abstract

Introduction: All physicians must learn comprehensive patient care delivery within the electronic health record (EHR). No studies have considered EHR communication training with an emphasis on clinical efficiency. This curriculum provides a method of teaching clinic efficiency while practicing effective patient communication in any EHR clinical situation. The target audience is resident physicians, fellow physicians, faculty physicians, and physician extenders practicing in a primary care setting where the EHR is present. **Methods:** This curriculum of four separate workshops provides a structured EHR approach while addressing communication strategies for preclinical preparation, rapport building, encounter initiation, agenda setting, and visit closure. The curriculum contains interactive presentations, tools, and an evaluation survey. Presenting efficiency issues with the EHR using the ATTEND mnemonic and agenda setting allows documentation while practicing communication techniques that maximize efficiency. **Results:** Postworkshop surveys revealed that participants felt the workshops were helpful (84%). One measurement of efficiency revealed improvement through decreased number of days to note completion after workshop participation. At the Program Directors Workshop, curriculum value was demonstrated by high attendance, with 94% feeling the workshops provided easily utilizable strategies. **Discussion:** The curriculum utilized only the EPIC EHR but would be generalizable. Future directions could include measurement of effective communication and visit efficiency through direct observation and expanded EHR timing data.

Keywords
Communication, Efficiency, EHR, Electronic Health Record, Agenda Setting, ATTEND, EMR, Electronic Medical Record, Communication Skills

Educational Objectives

By the end of this activity, learners will be able to:

1. Describe specific needs for provider efficiency education for maximizing use of the electronic health record (EHR).
2. Apply effective communication skills via an approach utilizing preclinic preparation, agenda setting, and visit closure within the EHR to be more efficient.
3. Demonstrate a new approach to using certain communication skills such as establishing rapport with the ATTEND method and agenda setting while utilizing the EHR to allow communication to increase clinic efficiency.

Citation:
Skelly K, Shen W, Wilbur J, et al. A curriculum for teaching clinical efficiency focusing on specific communication skills while maximizing the electronic health record. *MedEdPORTAL*. 2020;16:10989.
https://doi.org/10.15766/mep_2374-8265.10989

Introduction

Faculty and residents must provide comprehensive patient care delivery where the electronic health record (EHR) is part of the clinical setting. While physician providers are introduced to their EHR, there is no curriculum currently utilized in residency education offering a comprehensive approach to EHRs. Most resident training occurs in the setting of work-based learning while providing patient care. Effective use of communication skills during patient encounters can improve clinic efficiency.[1] Reviewing five models of doctor-patient communication strategies, the Kalamazoo Consensus Statement outlines a process emphasizing efficient clinical care while providing good communication.[1] This information is routinely provided to resident learners, but there is little didactic training on how to utilize these communication skills while working within the EHR. Without addressing communication skills within the EHR, there is increased risk for poor communication and less efficient or effective visits. In addition to the need for navigating the

Copyright © 2020 Skelly et al. This is an open-access publication distributed under the terms of the Creative Commons Attribution license.

EHR while still providing effective communication, physicians need efficient clinic time to be able to see the patients and provide safe, effective medical care. Through a focus on specific communication skills around the EHR, physicians can increase clinical efficiency while providing medical care.

Unlike other *MedEdPORTAL* efficiency workshops for specific situations, such as in the emergency room or working with consultants,[2,3] this workshop provides a scripted approach to teach efficiency while practicing good patient communication in any clinical situation with the EHR. Other *MedEdPORTAL* EHR resources include curricula on patient-centered care[4,5] and teaching oncologic documentation and billing[6] without addressing efficiency. The aim of this workshop is to offer a structured comprehensive approach to using the EHR while addressing key communication strategies as well as clinic efficiency. The target audience for this workshop is resident physicians, fellow physicians, faculty physicians, and physician extenders who practice in a primary care setting where the EHR is present.

Methods

Workshop Development

We began with a needs assessment from the University of Iowa Department of Family Medicine faculty and residents (Appendix A), asking about the need for learning efficiency in clinic.

Process

Our initial sessions were held at the University of Iowa Family Medicine Residency Program during four separate noon conference sessions, each of which lasted 45 minutes. Faculty facilitators were academic family medicine faculty physicians who researched and created the curriculum. Participants included 21 residents and 13 faculty physicians but varied from session to session, as we did not have the same participants at each workshop. Recruitment was done using word of mouth and email. Each workshop ended with an assignment that would be utilized to start the subsequent workshop though participants were not required to attend every workshop. Materials needed included a classroom with computer and projector to allow PowerPoint as well as space for small-group discussion and printed materials. If participants had opportunity to bring laptops allowing access to their EHR, it was helpful but not necessary. Below is an overview of each 45-minute session.

Workshop 1: Setting up the Template/Working in the EHR (Appendix B)

The session introduction (10 minutes) began with a discussion of the challenges facing clinic efficiency. Via PowerPoint, we presented a brief rationale for working within the EHR utilizing Makoul's essential elements of clinic practice, which emphasized good communication and included preclinical preparation, rapport building, encounter initiation, agenda setting, and visit closure (10 minutes).[1] We invited discussion about how people prepared for clinic (5 minutes). Utilizing the PowerPoint screenshot of the EHR, learners were given instruction on how to optimize the EHR navigators. Specific details on how to do preclinic preparation by creating a template bringing in past clinic note details, as well as past medical history, family history, and social history for each patient, were reviewed using the EHR snapshot. This gave each provider the opportunity to create a template with this same basic information from the EHR (Appendix C). Teamwork was emphasized with recommendations to work with the EHR dashboard for good communication (15 minutes). Each workshop ended with questions and answers as well as an assignment to practice the skill in real time with real patients in the EHR prior to next workshop. This workshop assignment was to create the template and try using it. Each subsequent workshop began with a discussion on the success or failures of the skill practice from the previous workshop.

Workshop 2: Preclinic Preparation and Rapport Building (Appendix D)

The session began with a discussion about how participants prepared for clinic, what worked and did not work with the template, and getting the EHR ready (5 minutes). Screenshots of the EHR were shared, with recommendations about how to change the EHR to make it more efficient (5 minutes). Learners were introduced to the ATTEND mnemonic as a tool (Appendix E) to allow better physician communication while using the EHR (5 minutes). The ATTEND steps were explained to the attendees: (A) acquaint themselves with the EHR, (T) take time to establish rapport prior to EHR use, (T) triangulate the computer so the patient can see, (E) use the EHR to engage and educate the patient, (N) no more screen—disengage from the EHR when discussing sensitive information and emotions, and (D) do not forget to log out when leaving the room.

Literature was reviewed for benefits of rapport and good communication. Active listening and use of time were reviewed to highlight the benefits of timing and good communication, with the recommendation that learners focus on overall patient rapport as they took time to practice this tool and utilize the EHR (15 minutes). Participants divided into pairs with the opportunity to role-play the ATTEND process within the EHR and share how they established rapport (10 minutes). The workshop ended with questions and answers as well as an assignment to practice

Copyright © 2020 Skelly et al. This is an open-access publication distributed under the terms of the Creative Commons Attribution license.

rapport and active listening skills in the HER in real time with real patients because we planned to begin the next workshop with a discussion on the success or failures of this skill.

Workshop 3: Agenda Setting and Relationship Maintenance (Appendix F)
The session started with discussion of what went well and what did not regarding the ATTEND method with using the EHR while keeping rapport with patients. Participants created a group list about what took time in getting a patient history (5 minutes). Agenda setting and screening were reviewed along with a specific method of agenda setting in the EHR (5 minutes). Information on the success of agenda setting was provided to learners highlighting the benefits of avoiding the "by the way" phenomenon and increasing patient motivation and involvement,[7,8] as well as emphasizing how interruptions are common and do not meet patient expectations.[9-12] Participants were then provided with a role-play activity to practice agenda setting as described in the Establishing Focus Protocol[13] to increase clinic visit efficiency while emphasizing good communication (15 minutes). One participant took the role of the provider while another was the patient, with the patient giving specific feedback about the experience to the provider (Appendix G). Different ways to utilize the EHR by putting the agenda list into the history of present illness during the visit were discussed (5 minutes). The workshop ended with questions and answers as well as an assignment to practice agenda setting while continuing to use ATTEND and active listening skills while in clinic because the next workshop would begin with a discussion on the success or failures of this skill.

Workshop 4: Visit Closure (Appendix H)
The session began with discussion of challenges and successes thus far (5 minutes). Key points from previous workshops were reviewed, including the recommended efficient EHR templates, ATTEND communication, and agenda setting (5 minutes). Visit closure was emphasized and reviewed within the EHR, where one method of communicating the discharge plan could populate both clinical visit notes and the after-visit summary (5 minutes). In order to emphasize how good communication should be completed with signaling closure, summarization, and a check for understanding about the discharge plan, participants divided into groups of three to perform the closure case activity. Attendees received a closure card (Appendix I) and a closure case worksheet. Each attendee had an opportunity to be the provider with a different attendee serving as the patient as they worked through the three different closure cases (Appendix I) with role-play (10 minutes). At this workshop, participants were again given the opportunity to set up after-visit summary templates for use in the EHR to emphasize after-visit plans (5 minutes). Participants also had the chance to discuss future needs to impact efficiency within the EHR.

Evaluation
Workshop evaluations were completed after each individual workshop and after the entire workshop series (Appendix J). Time to note completion within the EHR was compared utilizing pre- and postworkshop attendee note-completion times collected for all resident clinic notes for 3 months before and 3 months after the workshop series was presented. This was computed for all residents independent of whether they attended the workshops or not and was calculated based on the times a note was opened and then signed final. Institutional review board approval was obtained prior to gathering these data (University of Iowa IRB-01 Biomedical, #201509747).

We evaluated whether this curriculum was applicable outside the University of Iowa Family Medicine Residency Program when we presented this curriculum or its results at both the Society for Teachers of Family Medicine Annual Spring Meeting[14] and the American Academy of Family Physicians' Program Directors Workshop (PDW).[15] At these meetings, the curriculum was condensed, with an overview of all workshops where the attendees did not fully complete every activity they could utilize. Participants completed evaluation of the session.

Results

Quantitative Data
A preworkshop survey/needs assessment ($n = 22$) revealed that over 90% (20 of 22) of residents and faculty recognized deficiencies in their clinic efficiency skills (Figure). At the PDW, the clear value of the topic was demonstrated with high attendance, where 86 faculty learners participated and were given this structured approach to efficient communication with the EHR. The evaluation and feedback survey results found that 89% of participants felt the presentation was clear with effective teaching methods, 94% felt the workshop provided practical knowledge and strategies that could immediately be applied to a residency program, and 98% felt the content could be easily utilized.

Qualitative Data
Evaluation after presentation of the four workshops utilizing a survey of faculty and residents who participated in at least one workshop revealed that participants valued the workshops. Comments showed that most participants felt the workshops were very helpful (84%), with the majority feeling that agenda setting was the most helpful aspect. The faculty participants felt that poor agenda setting was one of the main reasons

Copyright © 2020 Skelly et al. This is an open-access publication distributed under the terms of the Creative Commons Attribution license.

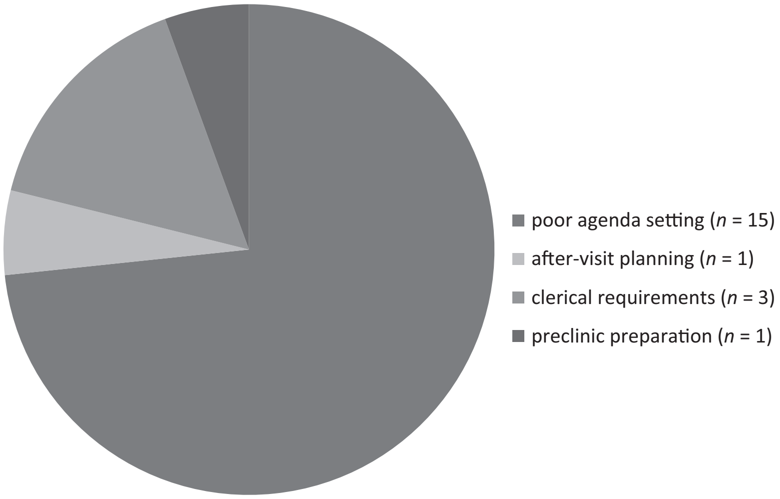

Figure. What slows clinic the most, according to 20 of 22 preworkshop survey/needs assessment respondents.

that slowed residents most during their clinics. Utilizing the workshops' multiple interactive presentations, tools, and survey for measuring satisfaction, learners felt they could teach a standardized workflow addressing issues of practicing agenda setting, signposting the EHR, and providing visit closure and effective written communication.

Additionally, pre- and postworkshop for all residents, independent of whether they attended the workshops or not, we measured and compared time to note completion within the EHR as one measurement of efficiency. We found that the number of days decreased after the workshop series (Table).

Discussion

This curriculum provides a framework for educators to utilize a stepwise approach to communication strategies in an efficient clinic visit within the EHR. While efficiency can be defined in multiple ways, one definition describes maximized patient care with minimal use of resources, including physician, health care team, and patient time.[16] Focusing on provider time, this framework could be applicable within any type of outpatient clinic setting utilizing the EHR. While the amount of information presented is a challenge, by breaking it into separate workshops with time to practice and utilize what was learned in one workshop prior to the next, the opportunity to make the information applicable is present. The purpose is not to be a comprehensive review of best patient communication methods but instead to utilize one recognized expert method to explore this issue. Our unique way of teaching this proven communication method within the EHR highlights communication while emphasizing efficiency. Additionally, the curriculum uses both agenda setting and the ATTEND method to focus on efficiency within the EHR.[13]

Table. Pre- vs. Posttraining Results ($N = 22$): Time to Note Completion

Pretraining Date	No. of Days	Posttraining Date	No. of Days
October	5.2	January	4.5
November	3.7	February	3.7
December	4.2	March	3.8
Average	4.4	Average	4.0

The workshop series is limited by utilizing only the EPIC EHR, but it could be generalized to any EHR after testing for the ability to replicate this process. Because of the prevalence of EPIC, the curriculum is currently applicable to many learners. While scheduling the workshops could provide challenges for participants to successfully implement the learning series, it can be done in multiple settings or subdivided as time allows. Additionally, variable learner skill levels and interest provide opportunities to adjust the material presented.

Several limitations are present in the data from the initial needs assessment and the evaluations. First, they were all surveys with relatively small sample sizes. Additionally, in the initial residency series, the same people did not attend all workshops, and we did not track which people attended how many of the workshops to evaluate the impact of number attended. We did not measure the communication skills or degree of efficiency at baseline for the participants. The series also may not be as beneficial for those who already have good communication skills and are already efficient with both patient care and the EHR. Clearly, there are future opportunities to improve survey data collection and evaluation to clarify the impact of the workshops.

Copyright © 2020 Skelly et al. This is an open-access publication distributed under the terms of the Creative Commons Attribution license.

Only one measurement of efficiency was evaluated after this workshop series: the time to note completion in the EHR. Time to note completion was tracked in the EHR only for the 3 months prior to the workshop series and the 3 months after the series in all resident physicians independent of whether they attended any of the workshops, making the intervention less measurable. While time to note completion suggested improvement in efficiency, the measurement was only a gross one that did not account for other things impacting note-completion time. We also collected the time spent for only 3 months and so cannot determine if these initial postsession gains were sustained.

While efficiency may have modest improvement and learners appear to like the curriculum, the measurements of the workshop do not address potential downsides of the intervention. The note-completion time is only one measurement and does not evaluate time spent with the patient, time spent reviewing the chart, and additional factors that impact overall efficiency while directly affecting the patient encounter. Determining patient satisfaction related to efficiency, time spent with the patient before and after the intervention, and even physician satisfaction could be a better measurement than just time to note completion. There is an opportunity to study this further with future workshops and additional linking to attendees as well as further elimination of confounding variables in EHR timing.

Despite the limitations, the positive subjective response in the participant evaluations from the PDW suggests possible opportunities for implementing this curriculum. Future directions could include measurement of effective communication, patient and provider satisfaction, and visit efficiency through direct observation, satisfaction surveys, and EHR timing data.

This innovative workshop curriculum provides an educational approach to addressing efficiency in the EHR without losing sight of good patient communication in residency education. It offers a hands-on interactive learning experience that can be translated directly into any EHR with ease.

Appendices

A. Efficiency Preworkshop Needs Assessment Survey.docx

B. Workshop 1 - Setting up the Template and Working in EHR.pptx

C. Sample Clinic Note and AVS Template.docx

D. Workshop 2 - Preclinic Preparation and Rapport Building.pptx

E. Efficiency ATTEND Practice Card.docx

F. Workshop 3 - Agenda Setting and Relationship Maintenance.pptx

G. Efficiency Agenda Setting Practice.docx

H. Workshop 4 - Visit Closure.pptx

I. Efficiency Closure Card and Cases.docx

J. Efficiency Postworkshop Evaluation.docx

All appendices are peer reviewed as integral parts of the Original Publication.

Kelly Skelly, MD: Associate Professor, Department of Family Medicine, University of Iowa Roy J. and Lucille A. Carver College of Medicine

Wendy Shen, MD: Associate Professor, Department of Family Medicine, University of Iowa Roy J. and Lucille A. Carver College of Medicine; ORCID: https://orcid.org/0000-0001-6545-8280

Jason Wilbur, MD: Professor, Department of Family Medicine, University of Iowa Roy J. and Lucille A. Carver College of Medicine; ORCID: https://orcid.org/0000-0002-5004-9115

Kate Thoma, MD: Associate Professor, Department of Family Medicine, University of Iowa Roy J. and Lucille A. Carver College of Medicine; ORCID: https://orcid.org/0000-0002-2963-8839

Jill Endres, MD: Professor, Department of Family Medicine, University of Iowa Roy J. and Lucille A. Carver College of Medicine; ORCID: https://orcid.org/0000-0002-1136-3400

Alison Lynch, MD: Professor, Department of Family Medicine, University of Iowa Roy J. and Lucille A. Carver College of Medicine; ORCID: https://orcid.org/0000-0002-0966-6605

Anne Gaglioti, MD: Associate Professor, Department of Family Medicine, Morehouse School of Medicine; ORCID: https://orcid.org/0000-0001-5843-8476

Marcy Rosenbaum, PhD: Professor, Department of Family Medicine, University of Iowa Roy J. and Lucille A. Carver College of Medicine; ORCID: https://orcid.org/0000-0002-8000-5711

Disclosures
None to report.

Funding/Support
None to report.

Ethical Approval
The University of Iowa Institutional Review Board approved this study.

References

1. Makoul G. Essential elements of communication in medical encounters: the Kalamazoo consensus statement. *Acad Med*. 2001;76(4):390-393. https://doi.org/10.1097/00001888-200104000-00021

Copyright © 2020 Skelly et al. This is an open-access publication distributed under the terms of the Creative Commons Attribution license.

2. Chen DC , Miloslavsky EM, Winn AS, McSparron JI. Fellow as Clinical Teacher (FACT) curriculum: improving fellows' teaching skills during inpatient consultation. *MedEdPORTAL*. 2018;14:10728. https://doi.org/10.15766/mep_2374-8265.10728
3. Witkowski J, Rassbach C. Efficiency in the pediatric emergency department: a choose your own adventure for pediatric residents and medical students. *MedEdPORTAL*. 2015;11:10214. https://doi.org/10.15766/mep_2374-8265.10214
4. Lee EL, Chisty A, Williams P, Brady W. An innovative curriculum using a simulated electronic health record to teach internal medicine residents about ICD–10-CM. *MedEdPORTAL*. 2017; 13:10538. https://doi.org/10.15766/mep_2374-8265.10538
5. Alkureishi M, Lee WW, Farnan J, Arora V. Breaking away from the iPatient to care for the real patient: implementing a patient-centered EMR use curriculum. *MedEdPORTAL*. 2014;10:9953. https://doi.org/10.15766/mep_2374-8265.9953
6. Patel A, Ali A, Lutfi F, Nwosu-Iheme A, Markham MJ. An interactive multimodality curriculum teaching medicine residents about oncologic documentation and billing. *MedEdPORTAL*. 2018;14:10746. https://doi.org/10.15766/mep_2374-8265.10746
7. Kroenke K. Patient expectations for care: how hidden is the agenda? *Mayo Clin Proc*. 1998;73(2):191-193. https://doi.org/10.1016/s0025-6196(11)63654-9
8. Towle A, Godolphin W. Framework for teaching and learning informed shared decision making. *BMJ*. 1999;319(7212): 766-769. https://doi.org/10.1136/bmj.319.7212.766
9. Marvel MK, Epstein RM, Flowers K, Beckman HB. Soliciting the patient's agenda: have we improved? *JAMA*. 1999;281(3):283-287. https://doi.org/10.1001/jama.281.3.283
10. Braddock CH III, Edwards KA, Hasenberg NM, Laidley TL, Levinson W. Informed decision making in outpatient practice: time to get back to basics. *JAMA*. 1999;282(24):2313-2320. https://doi.org/10.1001/jama.282.24.2313
11. Marple RL, Kroenke K, Lucey CR, Wilder J, Lucas CA. Concerns and expectations in patients presenting with physical complaints: frequency, physician perceptions and actions, and 2-week outcome. *Arch Intern Med*. 1997;157(13):1482-1488. https://doi.org/10.1001/archinte.1997.00440340122012
12. Kravitz RL, Callahan EJ, Paterniti D, Antonius D, Dunham M, Lewis CE. Prevalence and sources of patients' unmet expectations for care. *Ann Intern Med*. 1996;125(9):730-737. https://doi.org/10.7326/0003-4819-125-9-199611010-00004
13. Mauksch LB, Hillenburg L, Robins L. The Establishing Focus Protocol: training for collaborative agenda setting and time management in the medical interview. *Fam Syst Health*. 2001;19(2):147-157. https://doi.org/10.1037/h0089539
14. Skelly K, Shen W, Rosenbaum M, Wilbur J. Teaching residents clinical efficiency while using the electronic medical record and the ATTEND mnemonic. Presented at: Society of Teachers of Family Medicine Annual Spring Meeting; May 4, 2016; Minneapolis, MN.
15. Skelly K, Shen W, Wilbur J. Teaching residents clinical efficiency while using the electronic medical record and ATTEND mnemonic. Workshop presented at: AAFP PDW and RPS Residency Education Symposium; March 25, 2017; Kansas City, MO.
16. Cylus J, Papanicolas I, Smith PC, eds. *Health System Efficiency: How to Make Measurement Matter for Policy and Management*. European Observatory on Health Systems and Policies; 2016. Health Policy Series no. 46.

Received: November 7, 2019
Accepted: May 10, 2020
Published: October 29, 2020

Copyright © 2020 Skelly et al. This is an open-access publication distributed under the terms of the Creative Commons Attribution license.
